# (ADP-ribosyl)hydrolases: Structural Basis for Differential Substrate Recognition and Inhibition

**DOI:** 10.1016/j.chembiol.2018.11.001

**Published:** 2018-12-20

**Authors:** Johannes Gregor Matthias Rack, Antonio Ariza, Bryon S. Drown, Callum Henfrey, Edward Bartlett, Tomohiro Shirai, Paul J. Hergenrother, Ivan Ahel

**Affiliations:** 1 Sir William Dunn School of Pathology, Oxford University, South Parks Road, Oxford OX1 3RE, UK; 2 University of Illinois, Department of Chemistry, Urbana, IL 61801, USA; 3 Kyoto Institute of Technology, Matsugasaki Hashikamicho, Sakyo Ward, Kyoto, Japan

**Keywords:** ADPRH, ADPRHL2, DNA damage, ADP-ribosylation, PARP, PARG, metalloenzyme

## Abstract

Protein ADP-ribosylation is a highly dynamic post-translational modification. The rapid turnover is achieved, among others, by ADP-(ribosyl)hydrolases (ARHs), an ancient family of enzymes that reverses this modification. Recently ARHs came into focus due to their role as regulators of cellular stresses and tumor suppressors. Here we present a comprehensive structural analysis of the enzymatically active family members ARH1 and ARH3. These two enzymes have very distinct substrate requirements. Our data show that binding of the adenosine ribose moiety is highly diverged between the two enzymes, whereas the active sites harboring the distal ribose closely resemble each other. Despite this apparent similarity, we elucidate the structural basis for the selective inhibition of ARH3 by the ADP-ribose analogues ADP-HPD and arginine-ADP-ribose. Together, our biochemical and structural work provides important insights into the mode of enzyme-ligand interaction, helps to understand differences in their catalytic behavior, and provides useful tools for targeted drug design.

## Highlights

• ARH1 and ARH3 have high structural similarity, but different modes of ligand binding
• Structures unveil reason for the observed selectivity of α-1″-linked substrates
• ARH3, but not ARH1, is susceptible to product (analogue) inhibition
• Displacement of a catalytic Mg^2+^ ion leads to inhibition (e.g., by ADP-HPM or CaCl_2_)

## Graphical Abstract

**Figure:**
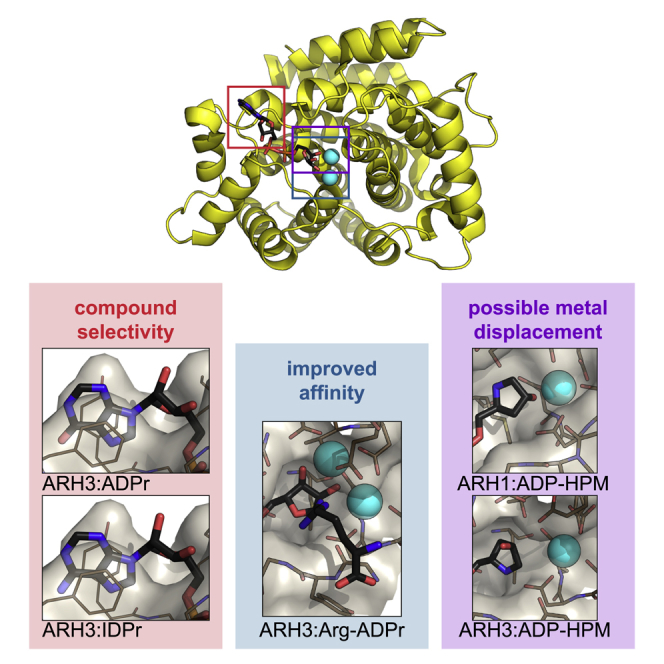

## Teaser

Rack et al. describe the atomic resolution structures of the (ADP-ribosyl)hydrolases ARH1 and ARH3 in their ligand and inhibitor bond forms. The structures provide a molecular basis for their differential inhibition by these inhibitors and may aid ongoing efforts to elucidate the physiological function of ARH1/3.

## Introduction

**Figure 1:**
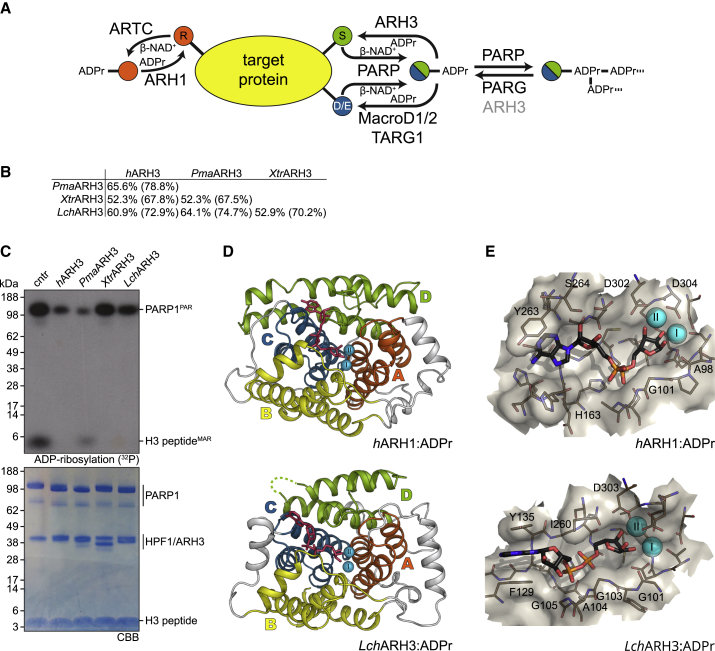
Functional and Structural Overview of ARH1 and ARH3 (A) Scheme of vertebrate ADP-ribosylation reactions. The modification of a target protein can occur as MARylation on arginine residues (orange) catalyzed by ARTCs, as well as MARylation and PARylation on glutamate/aspartate (blue) and serine (green) residues catalyzed by PARPs. Arginine de-modification is catalyzed by ARH1, PARylation is removed by PARG and to a lesser extend ARH3, MARylation on glutamate/aspartate residues is hydrolyzed by macrodomain proteins, whereas the terminal modification on serine residues is removed by ARH3. (B) Pairwise sequence identity comparison of selected ARH3 proteins. Sequence identity and similarity (in parentheses) are provided. (C) (ADP-ribosyl)hydrolase activity assessment of selected ARH3 orthologues. All ARH3s efficiently remove MARylation from the histone H3 peptide (aa 1-20) and degrade PARP1 generated PARylation to a variable extent. (D) Ribbon representation of *h*ARH1 and *Lch*ARH3 in complex with ADPr (red) showing quasidomains A (orange), B (yellow), C (blue), and D (green) as well as the coordinated Mg^2+^ ions (cyan). (E) Liquorice-surface representation of *h*ARH1 and *Lch*ARH3 (brown) in complex with ADPr (black). Residues important for the interaction are highlighted (see Figures S1–S3 for further details). See also Figures S1–S3.

ADP-ribosylation is a dynamic post-translational modification involved in the regulation of a wide variety of cellular processes, including DNA damage response (DDR), aging, immunity, bacterial metabolism, and many others (Fehr et al., 2017, Gupte et al., 2017, Palazzo et al., 2017). It is established by the stereospecific transfer of ADP-ribose (ADPr) from β-NAD^+^ onto a target residue, which results in the formation of an α-anomeric ADP-ribosylated amino acid and the release of nicotinamide (Sung, 2015). In eukaryotes, this reaction is catalyzed by two distinct families of ADP-ribosyltransferases (ARTs) classified by their catalytic motifs as well as their relationship to bacterial exotoxins: (1) cholera toxin-like ARTs (ARTC) containing an R-S-E motif and (2) diphtheria toxin-like ARTs (PARPs, also called ARTDs) containing an H-Y-E motif (Hottiger et al., 2010, Palazzo et al., 2017). Modification of residues containing acidic (glutamate/aspartate), basic (arginine/lysine), hydroxyl (serine), and thiol (cysteine) moieties have been described (Cohen and Chang, 2018, Leidecker et al., 2016, Vyas et al., 2014). Specificity for these residues is partially the result of distinct structural arrangements in the ART catalytic domain, with ARTCs usually catalyzing the transfer onto arginine residues and PARPs usually modifying acidic residues (Crawford et al., 2018, Laing et al., 2011). Recently, we have shown that for PARPs this canonical activity can be altered in the mammalian DDR through the formation of a complex of PARP1 or PARP2 with histone PARylation factor 1 (HPF1), which leads to a preference for serine modification in many proteins involved in the maintenance of genomic stability (Bonfiglio et al., 2017, Gibbs-Seymour et al., 2016). In addition, a subset of PARPs can transfer further ADPr units onto the initial modification, forming polymers of ADPr units, known as poly(ADP-ribosyl) (PAR) modification (Figure 1A) (D'Amours et al., 1999). Reversal of the bulk of PAR modification is mediated by poly(ADP-ribosyl)glycohydrolase (PARG) converting the polymer chain to a mono(ADP-ribosyl) (MAR) modification (Lin et al., 1997, Slade et al., 2011). PARG is unable to efficiently cleave the protein-linked ADPr moiety (Slade et al., 2011), which requires a number of other hydrolases. For example, linkages to glutamate/aspartate are hydrolyzed by macrodomain proteins (Jankevicius et al., 2013, Rosenthal et al., 2013, Sharifi et al., 2013), whereas linkages to arginine and serine are cleaved by ARH1 and ARH3, respectively, two members of the structurally unrelated (ADP-ribosyl)hydrolases (ARHs) family (Fontana et al., 2017, Moss et al., 1988) (Figure 1A). In addition, ARH3 can cleave PAR chains, 1″-*O*-acetyl-ADPr and ADPr at the phosphorylated DNA ends, although these activities have not been confirmed *in vivo* so far (Mueller-Dieckmann et al., 2006, Munnur and Ahel, 2017, Ono et al., 2006).

Macrodomains are structurally well characterized and several structures are available of their apo- and ligand-bound forms, including at least one representative of each macrodomain class found in humans (MacroH2A-like, MacroD-type, ALC-like and PARG-like) (reviewed in Barkauskaite et al., 2015, Rack et al., 2016). In contrast, eukaryotic ARH family members are poorly understood and thus far only apo structures for ARH3 are available in the literature (Mueller-Dieckmann et al., 2006, Mueller-Dieckmann et al., 2008), and one unpublished ADP-bound structure of ARH1 is deposited in the Protein Databank (PDB: 3HFW).

Although ARHs catalyze reactions that appear to be unique in higher organisms, very little is known about their physiological function. So far we know that ARH1 knockout mice present an increased rate of tumorigenesis, as well as a higher susceptibility to bacterial infections involving ARTC-type exotoxins (Kato et al., 2007, Kato et al., 2011). ARH3 plays a role in neurodegenerative disorders, the oxidative stress response and DDR (Ghosh et al., 2018, Mashimo et al., 2013, Palazzo et al., 2018). Mouse embryonic fibroblasts derived from ARH3^−/−^ mice are susceptible to hydrogen peroxide-induced cell death, while human cells deficient in ARH3 exhibit increased levels of serine MARylation (Mashimo et al., 2013, Palazzo et al., 2018). Little is known about the third family member, ARH2, but recent reports showed that it is primarily expressed in heart tissue and appears to be involved in the regulation of heart chamber outgrowth (Smith et al., 2016).

Here we present structures of both ARH1 and ARH3 in their ligand-bound forms and provide evidence for their different modes of substrate recognition as well as selective inhibition by ADPr analogues. We demonstrate that ARH3, but not ARH1, is inhibited by ADP-HPD and elucidate the molecular basis for this difference. Together, our data provide the important insights into the mode of substrate recognition and reveal features important for substrate selectivity of (ADP-ribosyl)hydrolases.

## Results

In order to unveil details about the selective modes of ligand recognition and catalytic mechanisms of ARH family members, we aimed to produce the crystal structures of both ARH1 and ARH3 with their endogenous ligand. As demonstrated earlier, production of crystals of human ARH3 (*h*ARH3) containing ADPr proved to be very challenging (Kernstock et al., 2006, Mueller-Dieckmann et al., 2006). Therefore, we used a combination of sequence analysis, protein crystallizability prediction (XtalPred, http://ffas.burnham.org/XtalPred-cgi/xtal.pl) (Slabinski et al., 2007) and biochemical analysis to identify suitable orthologous targets. We selected ARH3 orthologues with high similarity to the human protein from the bird great tit (*Parus major*; *Pma*ARH3), tropical clawed frog (*Xenopus tropicalis*; *Xtr*ARH3) and fish gombessa (West Indian Ocean coelacanth, *Latimeria chalumnae*; *Lch*ARH3) (Figure 1B) and screened the properties of these enzymes. All three proteins efficiently removed serine MARylation, with somewhat varying activities against PAR (Figure 1C). We then solved the crystal structures of apo- and ADPr-bound *Lch*ARH3, as well as the structure of the *h*ARH1:ADPr complex (Table 1). *Lch*ARH3 and *h*ARH1 share low sequence conservation (20.1% identity, 33.4% similarity) and an overall structural root mean square deviation (RMSD) of 3.2 Å over 160 C^α^. Despite this divergence, models of both enzymes follow the earlier observed fold of a tightly packed, mainly α-orthogonal bundle separated into four quasidomains (A–D) with a binuclear Mg^2+^ center (Figures 1D and 1E). Initial crystals of *Lch*ARH3 had only one Mg^2+^ ion bound (isostructural to Mg_I_ in ARH1, Figure S1A). Later crystals were grown in the presence of MgCl_2_, which promoted coordination of a second Mg^2+^ ion in the *Lch*ARH3:ADPr structure, but not the apo form. The structures are complete with the exception of a loop region between residues Gly210 and Lys228 for which no electron density could be observed. Comparison of our models with the closest relatives in the PDB showed little divergence from previously solved structures (Table S1).

**Table 1:** Data Collection, Phasing, and Refinement Statistics

	*h*ARH1: ADPr	*h*ARH1: ADP-HPM	*Lch*ARH3 apo	*Lch*ARH3: ADPr	*Lch*ARH3: ADPr	*Lch*ARH3: ADP-HPD	*Lch*ARH3: ADP-HPM	*Lch*ARH3: IDPr	*Lch*ARH3 Arg-ADPr	*h*PARG: ADP-HPM
PDB accession code	6G28	6G2A	6G1P	6G1Q	6HGZ	6HH3	6HH5	6HOZ	6HH4	6HH6

**Data Collection**										

Synchrotron/beamline	DLS/I03	DLS/I04	DLS/I04-1	DLS/I04	DLS/I24	DLS/I24	DLS/I24	DLS/I03	DLS/I24	DLS/I04-1
Wavelength (Å)	0.9760	1.0036	0.9159	0.9795	0.9686	0.9686	0.9686	0.9762	0.9686	0.9174
Space group	*P*2_1_ 2_1_ 2_1_	*C*2	*P*2_1_ 2_1_ 2_1_	*P*2_1_ 2_1_ 2_1_	*P*2_1_ 2_1_ 2_1_	*P*2_1_ 2_1_ 2_1_	*P*2_1_ 2_1_ 2_1_	*P*2_1_ 2_1_ 2_1_	*P*2_1_ 2_1_ 2_1_	*P*2_1_ 2_1_ 2_1_
a (Å)	64.10	98.53	64.96	66.90	66.79	66.94	66.86	66.85	66.12	67.33
b (Å)	66.69	42.94	99.88	97.46	97.64	98.27	98.17	96.95	96.69	90.88
c (Å)	83.42	89.23	107.63	105.50	106.45	105.58	105.49	107.52	105.87	95.28
α (°)	90.00	90.00	90.00	90.00	90.00	90.00	90.00	90.00	90.00	90.00
β (°)	90.00	118.93	90.00	90.00	90.00	90.00	90.00	90.00	90.00	90.00
γ (°)	90.00	90.00	90.00	90.00	90.00	90.00	90.00	90.00	90.00	90.00
Content of AU	1	1	2	2	2	2	2	2	2	1
Resolution (Å)^a^	66.69–1.23	78.09–1.80	99.88–1.55	56.16–2.10	71.95–1.86	44.55–1.82	44.50–1.95	96.95–1.77	71.39–1.66	54.09–1.85
	(1.25–1.23)	(1.84–1.80)	(1.58–1.55)	(2.16–2.10)	(2.01–1.86)	(1.87–1.82)	(2.00–1.95)	(1.82–1.77)	(1.70–1.66)	(1.89–1.85)
R_sym_ (%)^a^^,^^b^	5.9 (26.2)	7.0 (48.8)	5.1 (65.6)	8.5 (90.3)	9.2 (135.5)	10.1 (224.2)	10.5 (183.7)	12.0 (189.6)	8.9 (152.4)	22.1 (26.7)
I/σ(I)	21.7 (5.5)	12.8 (2.7)	22.8 (2.6)	13.8 (2.4)	14.4 (1.4)	11.2 (1.1)	8.3 (0.9)	10.9 (1.2)	9.9 (1.1)	10.0 (1.3)
Completeness (%)^a^	99.6 (97.1)	99.4 (99.5)	100.0 (99.4)	100.0 (100.0)	94.9 (87.7)	100.0 (100.0)	99.8 (99.3)	100.0 (100.0)	100.0 (100.0)	99.8 (99.2)
Redundancy^a^	12.2 (9.3)	6.6 (6.7)	10.9 (6.7)	13.1 (13.6)	9.0 (8.3)	12.7 (12.7)	8.5 (8.6)	12.5 (10.2)	8.3 (8.4)	13.3 (12.3)
CC_1/2_ (%)^a^	99.8 (98.1)	99.9 (88.6)	99.9 (77.5)	99.9 (83.6)	94.9 (59.7)	99.8 (87.5)	99.8 (81.4)	99.9 (74.5)	99.7 (67.6)	99.6 (59.7)
Unique reflections^a^	103868 (4932)	30,388 (1774)	102142 (4969)	41,057 (3339)	36,681 (1834)	63,174 (4589)	51,249 (3716)	68,856 (5010)	82,067 (5982)	50,546 (3055)

**Refinement**										

R_cryst_ (%)^c^	14.5	18.1	14.5	21.8	17.5	17.9	18.6	15.8	16.9	16.6
R_free_ (%)^d^	15.7	22.0	16.3	25.8	21.0	21.0	21.4	18.9	19.0	20.0
RMSD bond length (Å)	0.010	0.011	0.010	0.012	0.012	0.009	0.011	0.012	0.012	0.012
RMSD bond angle (°)	1.53	1.46	1.55	1.56	1.49	1.42	1.46	1.51	1.53	1.58

**Number of Atoms and Average B Factor** (Å^2^)^e^										

Protein	2919 [19.4]	2824 [31.0]	5387 [22.3]	5120 [62.7]	5103 [32.4]	5135 [47.8]	5107 [61.2]	5172 [31.0]	5188 [32.1]	4201 [25.4]
Water	356 [32.8]	133 [35.7]	592 [36.9]	70 [59.5]	278 [36.1]	259 [51.8]	257 [56.8]	421 [41.6]	529 [45.2]	507 [35.1]
Magnesium ion	2 [11.5]	2 [21.4]	2 [17.2]	2 [55.4]	4 [30.4]	4 [52.5]	2 [53.4]	4 [29.1]	4 [31.2]	–
ADP-ribose	36 [14.7]	–	–	72 [65.6]	72 [33.0]	–	–	–	–	–
ADP-HPD	–	–	–	–	–	–	68 [61.5]	–	–	34 [16.8]
ADP-HPM	–	34 [25.1]	–	–	–	70 [51.8]	–	–	–	–
IDPr^f^	–	–	–	–	–	–	–	144 [28.9]	–	–
Arg-ADP-ribose	–	–	–	–	–	–	–	–	94 [37.7]	–
Acetate ion	–	12 [41.7]	28 [55.4]	–	–	–	–	4 [53.9]	8 [61.1]	–
Chloride ion	3 [37.9]	1 [47.2]	–	–	–	–	–	–	–	–
Citrate ion	–	–	26 [22.2]	–	–	–	–	–	–	–
Sulfate ion	–	–	–	–	–	–	–	–	–	20 [68.2]
Glycerol	–	–	48 [46.7]	–	12 [61.9]	12 [77.4]	12 [88.4]	60 [58.5]	12 [49.1]	–

**Ramachandran Plot**										

Favored (%)	98.5	98.0	98.5	97.4	98.4	98.1	97.5	97.8	97.9	95.86
Allowed (%)	1.5	2.0	1.2	2.2	1.3	1.6	2.1	1.9	1.8	3.73
Disallowed (%)	0.0	0.0	0.3	0.5	0.3	0.3	0.5	0.3	0.3	0.4

See also Table S2.

a Data for the highest resolution shell are given in parentheses.

b R_sym_ = Σ|/-</>|/Σ/, where/is measured density for reflections with indices *hkl*.

c R_cryst_ = Σ||Fobs| - |Fcalc||/Σ|Fobs|.

d R_free_ has the same formula as R_cryst_, except that calculation was made with the structure factors from the test set.

e Data for the average B factor are given in brackets.

f Note, IDPr is modeled as a 1:1 dual conformer of the α- and β-anomer.

### ADPr Coordination

The binding of the reaction product (ADPr) by *h*ARH1 and *Lch*ARH3 has two distinct aspects: The recognition of the adenosine-proximal ribose group differs greatly between the two enzymes, whereas the pyrophosphate-distal ribose recognition is highly similar. Coordination of ADPr in *Lch*ARH3 is primarily achieved via contacts with quasidomains B and C (Figures 1D, 1E, S1F, and S2A). The adenosine moiety is held between Phe129 and Tyr135 by π-stacking interactions as well as polar contacts with Gly133, Ser134, and Tyr135, while the pyrophosphate makes extensive contact to the protein backbone (Gly105, Val106, and Gly138) and side chains (Ser134 and His168). This arrangement positions the 2′ and 3′ OH groups of the proximal ribose to face out of the ligand binding cleft and toward the aqueous environment with only a single protein contact (Gly105 with 3′ OH; Figures 1E and S1F). Comparison with the native structure shows that Gly103/Ala104/Gly105, which are part of loop 4 transitioning into helix α5, move out of the active site to accommodate the proximal ribose. In the native structure the closed conformation is stabilized by a co-crystallized citrate molecule, which interacts with Gly103 via its hydroxyl group and mimics the P^β^ contacts of ADPr to His167 and Gly138 via one of its carboxyl moieties (Figure S1B). In the *h*ARH1:ADPr complex, the interactions of the adenosine and proximal ribose occur primarily through quasidomain D due to a relative movement of the adenosine moiety by 8.4 Å and a rotation of ∼105° (Figures1D, 1E, S1E, and S2B). The adenosine group lies against the protein surface and interacts directly with Ser124, Gly127, and Ser270, while the interaction with Thr167 is bridged via a water molecule (w570). The environment-facing site is shielded by π stacking with Tyr263. The latter is part of a rigid adenosine binding loop (Ser262 to Gly271) which is stabilized by the presence of two structural water molecules (w577 and w618) and coordinates the proximal ribose via the 2′ and 3′ OH. The pyrophosphate coordination is comparable in its arrangement with ARH3 and involves interactions with the buried residues Gly130 and His163, which are absolutely conserved in both ARH1 and ARH3 (Figure S3), as well as a short non-conserved surface motif (Gly101/Ala102/Ser103). In contrast to ARH3, the latter are not part of a loop, but form the first turn of helix α8.

The different modes of interaction of the proximal ribose units in ARH1 and ARH3 have an important functional implication: exposure of the ribose to the aqueous environment in ARH3 allows the accommodation of consecutive ADPr units, which suggests an ability to accommodate both *endo*-chain linkages and protein-linked chains, whereas the tight coordination of the 2′ OH in ARH1 makes such a continuation less favorable.

### Magnesium Coordination and Active Site

**Figure 2:**
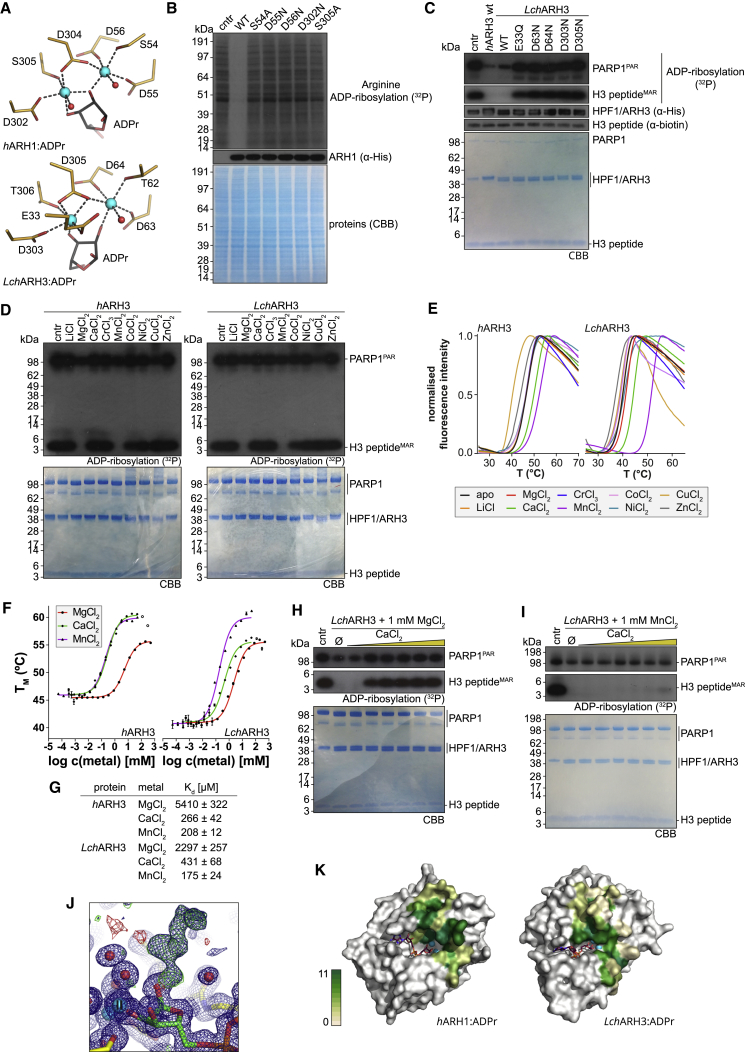
Ligand Coordination in *h*ARH1 and *Lch*ARH3 (A) Liquorice representation of the magnesium coordination within the *h*ARH1:ADPr and *Lch*ARH3:ADPr complexes. (B) Mutation analysis of metal coordinating residues of *h*ARH1. Cellular extracts from HeLa cells were arginine ADP-ribosylated by recombinant *m*ARTC2.2. The reactions were then supplemented with WT and mutant *h*ARH1 as indicated (see Figure S3 for further details). (C) Mutation analysis of metal coordinating residues of *Lch*ARH3. Histone H3 peptide (aa 1-20) was serine ADP-ribosylated by PARP1 in presence of HPF1. The reaction was stopped with olaparib and supplemented with WT and mutant *Lch*ARH3 as indicated (see Figure S3 for further details). (D) Analysis of the effect of metal substitution on ARH3 activity. Mg_II_ was stripped form *h*ARH3 and *Lch*ARH3 as described in STAR Methods and activity assay performed in presence of 100 μM of the indicated metal salt. (E) Assessment of the effect of metal substitution on ARH3 stability by differential scanning fluorimetry (DSF). Mg_II_-depleted *h*ARH3 and *Lch*ARH3 (described in STAR Methods) were supplemented with 100 μM of the indicated metal salt prior to the start of the assay. Curves represent normalized averages of triplicate (*h*ARH3) or sextuplicate (*Lch*ARH3) measurements (see Table 2 for further details). (F) K_d_ values were determined by DSF approach. Sixteen metal salt dilutions were distributed around the estimated K_d_ value. Melting temperature data were fitted using a single-site ligand-binding model. Note, the two highest calcium concentrations (open diamonds) were excluded from the fit as they consistently showed signs of increased protein instability. The represented data are derived from triplicate measurements ± SD. (G) Calculated K_d_ values of the data shown in (F). (H) Inhibition of *Lch*ARh3 by CaCl_2_ in presence of 1 mM MgCl_2_. The reactions were supplemented with increasing amounts of CaCl_2_ (1, 100, 250, 500, 1,000, and 2,000 μM) as indicated. (I) Inhibition of *Lch*ARh3 by CaCl_2_ in presence of 1 mM MnCl_2_. The reactions were supplemented with increasing amounts of CaCl_2_ (1, 100, 250, 500, 1,000, and 2,000 μM) as indicated. (J) Close up of the coordination of the distal ribose (green) of the *h*ARH1:ADPr structure. The electron-density map shows 2F_o_-F_c_ (blue) contoured at 1 σ and F_o_-F_c_ (green/red) contoured at ±3 σ. The positive density protruding from the 1″C could not be fitted using any known components of the crystallization solution, and therefore the ADPr moiety was fitted as the only known part of the ligand compound. (K) Surface representation of *h*ARH1 and *Lch*ARH3. The potential substrate binding surfaces are highlighted according to physicochemical conservation (with 0–10 for property conservation and 11 for absolute residue conservation) (Livingstone and Barton, 1993). The bound ADPr (purple) and Mg^2+^ ions (cyan) are given for orientation (see Figure S5 for further details). See also Figures S1–S3 and S5 and Table S2.

**Figure 3:**
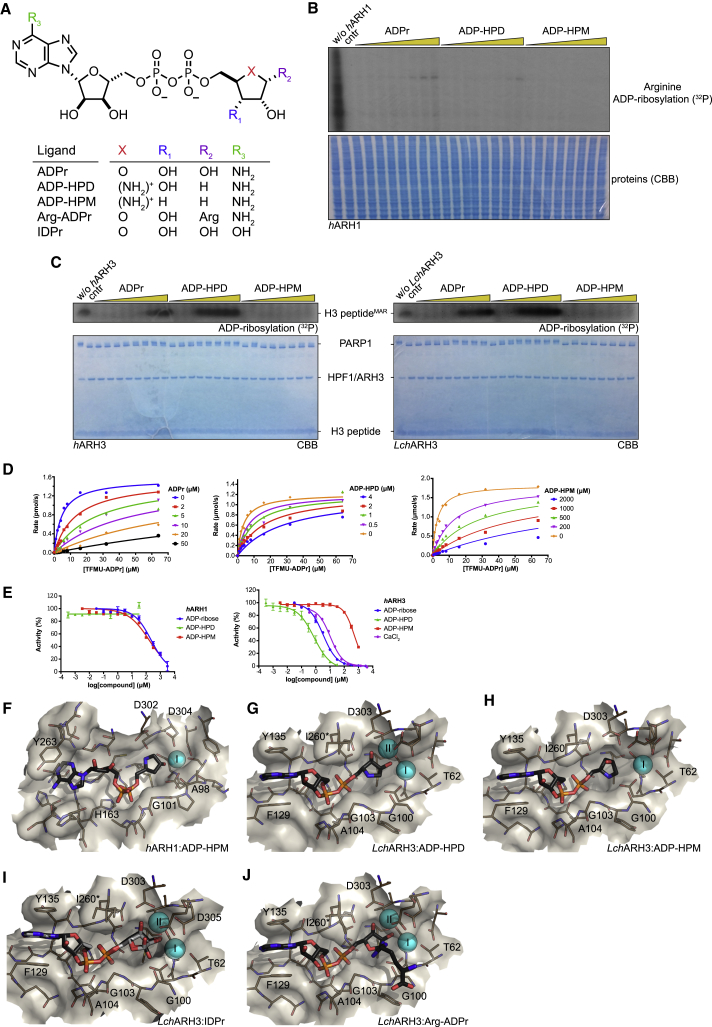
Interaction of ARH1 and ARH3 with ADPr Analogues (A) Chemical structures of ADPr and its analogues ADP-HPD, ADP-HPM, IDPr, and Arg-ADPr. Note, for ease of representation the minor tautomer of IDPr is shown. (B) Analysis of *h*ARH1 inhibition by ADPr, ADP-HPD, and ADP-HPM. The reactions were supplemented with WT *h*ARH1 and increasing amounts of compound (1, 5, 10, 50, 100, 250, 500, and 1,000 μM) as indicated. (C) Analysis of *h*ARH3 (left) and *Lch*ARH3 (right) inhibition by ADPr, ADP-HPD, and ADP-HPM. The reactions were supplemented with WT ARH3 and increasing amounts of compound (1, 5, 10, 50, 100, 250, 500, and 1,000 μM) as indicated (see Figure S4B for further details). (D) Kinetics of inhibition of *h*ARH3 by ADPr, ADP-HPD, and ADP-HPM. The represented data are derived from triplicate measurements ± SD. (E) Inhibition of *h*ARH1 and *h*ARH3 by ADPr, ADP-HPD, and ADP-HPM, as well as *h*ARH3 by CaCl_2_ using 20 μM TFMU-ADPr as substrate. The represented data are derived from triplicate measurements ± SD. (F–J) Liquorice-surface representation of the *h*ARH1 and *Lch*ARH3 binding cleft in presence of ADP-HPM (F + H), ADP-HPD (G), IDPr (I), or Arg-ADPr (J). Residues important for the interaction are highlighted and observed dual conformers indicated by an asterisk (see Figures S2 and S3 for further details). See also Figures S2 and S3.

The active sites of *h*ARH1 and *Lch*ARH3 are structurally very similar and contain residues for the coordination of two Mg^2+^ ions. Coordination of Mg_I_ occurs between quasidomains A and B, whereas Mg_II_ is coordinated solely by residues from quasidomain A (Figure 1D). The coordination motifs are only slightly diverged, but nevertheless distinct, between ARH1 (E … **SDD**T … *D*S***D****S*) and ARH3 (*E* … [**T**/**S**]**DD**T … *D*T***D****T*) (residues coordinating Mg_I_ are given in **bold** and Mg_II_ in *italic*) (Figures S3 and 3A). The motif aspartates are required to compensate the charge of Mg^2+^ ions and allow the tight packing of the ARH binuclear centers (average Mg-Mg distance of all available ARH structures: 3.66 ± 0.18 Å) (Yang et al., 2008). All observed Mg^2+^ ions have octahedral coordination involving the indicated residues, water molecules, and, in the case of the ADPr-containing structures, distal ribose hydroxyl moieties (Figure 2A, Table S2). Comparison of the magnesium coordination with other solved ARH3 structures revealed variation in the first coordination sphere of Mg_II_ with one ligand site either occupied by the carboxylic acid oxygen of an absolutely conserved glutamate residue (Glu33 in *Lch*ARH3) or a water molecule (Figure S1C). The absence of Mg_II_ from the *Lch*ARH3 apo and initial ADPr structure indicates either a weak coordination of the Mg^2+^ ion or its catalytic dispensability. Earlier studies suggested that both ARH1 and ARH3 require two Mg^2+^ ions for catalytic activity (Mueller-Dieckmann et al., 2006, Takada et al., 1993). In order to test this hypothesis, we performed de-MARylation assays with wild-type (WT) and mutants of *h*ARH1 and *Lch*ARH3 (Figures 2B and 2C). The activity of both enzymes is abolished upon mutation of even a single Mg^2+^ coordinating residue, hence strongly suggesting that the coordination of two metal ions is necessary for catalysis. To further investigate the metal-dependence of ARH3, we removed the magnesium ions from the protein by dialysis against ethylenediaminetetraacetate (EDTA). This treatment inactivated ARH3 through the removal of Mg_II_, as validated by crystallization of *Lch*ARH3 (data not shown). Full activity could be restored by addition of magnesium to the reaction (Figure S2C). Next we tested whether supplementation with different metal salts had an influence on catalytic activity and protein stability (Figures 2D and 2E). From the tested metal panel, only magnesium and manganese were able to support hydrolysis of Ser-ADPr and PAR (Figures 2D and S4A). Interestingly, calcium and manganese led to a significant increase in the thermal stability of both human and gombessa ARH3, whereas magnesium had only an influence on the stability of *Lch*ARH3 (Figure 2E, Table 2). The K_d_ of the ARH3:metal interaction follows the same trend and is comparable between *h*ARH3 and *Lch*ARH3 (Figures 3F and 3G). These data indicate that calcium may act as an inhibitor of magnesium-, but not manganese-, supplemented ARH3. We confirmed this hypothesis by de-MARylation assay run in presence of either Mg^2+^ or Mn^2+^ (Figures 2H, 2I, 3E, and S4B and Table 3). The occupation of the Mg_I_ site after EDTA treatment suggests that its primary function is structural, whereas restoration of the hydrolase activity after Mg^2+^-supplementation implies a catalytic role of Mg_II_. These findings make it tempting to speculate that the dynamic occupation of the Mg_II_ site may have evolved as a regulatory mechanism.

**Table 2:** Influence of Metal Substitution on ARH3 Stability

	*h*ARH3^a^		*Lch*ARH3^b^	
	T_M_ (°C)	ΔT_M_ (°C)^c^	T_M_ (°C)	ΔT_M_ (°C)^c^
apo	46.7 ± 0.05		40.2 ± 0.16	
LiCl	46.6 ± 0.15	−0.1	40.1 ± 0.16	−0.1
MgCl_2_	46.7 ± 0.11	0.0	41.4 ± 0.17	1.3^d^
CaCl_2_	50.1 ± 0.08	3.4^d^	44.4 ± 0.08	4.2^d^
CrCl_3_	46.5 ± 0.04	−0.3	40.1 ± 0.21	−0.1
MnCl_2_	52.6 ± 0.09	5.9^d^	51.5 ± 0.23	11.3^d^
CoCl_2_	46.7 ± 0.09	0.0	39.6 ± 0.28	−0.6
NiCl_2_	46.4 ± 0.09	−0.3	40.7 ± 0.22	0.5
CuCl_2_	40.5 ± 0.10	−6.2^d^	39.0 ± 0.21	−1.2^d^
ZnCl_2_	44.1 ± 0.07	−2.6^d^	37.4 ± 0.25	−2.8^d^

a n = 3.

b n = 6.

c ΔT_M_ = T_M_ (salt) - T_M_ (apo).

d p < 10^−5^.

**Table 3:** Inhibition Parameters of *h*ARH1 and *h*ARH3

Inhibitor	*h*ARH1		*h*ARH3	
	K_i_ (μM)	IC_50_ (μM)	K_i_ (μM)	IC_50_ (μM)
ADPr	ND	228	1.08 ± 0.08	3.2
ADP-HPD	ND	NA	0.58 ± 0.12	0.587
ADP-HPM	ND	164	54.2 ± 4	480
CaCl_2_	NA	ND	NA	13.7

All samples measured in triplicate. NA, not applicable; ND, not determined.

Our structural data further suggest that the presence of both Mg^2+^ ions is required for the correct positioning of the distal ribose. In the *h*ARH1 model, the 2″OH is located between and coordinated by both Mg^2+^ ions, while the 3″ OH makes contact with Mg_II_ as well as Asp302. In the electron density map, we observed positive density extending from the α-face of the anomeric carbon (Figure 2J). Similar densities could be observed in several crystals (data not shown). Earlier reports indicate that the reversal of the hydrolysis reaction of ARHs is possible under crystallographic conditions (Berthold et al., 2009), suggesting that the ADPr could have reacted with another molecule in the crystallization solution. Despite extensive screening of all known crystallization components and potential reaction products (including arginine and lysine), we were unable to identify the chemical nature of the missing entity. Yet, presence of this density supports the notion that the modeled ADPr is in a catalytically meaningful conformation. Comparison of the *h*ARH1:ADPr (PDB: 6G28, this study) and *h*ARH1:ADP (PDB: 3HFW) structures revealed conformational changes close to the active site, which are, however, not the results of the presence of the distal ribose, but the presence of a potassium ion in the *h*ARH1:ADP structure (Figure S1D, Table S2). The K^+^ ion distorts helix α2 and draws the residues closer to the active site, leading to the backbone displacement of residues Lys23 to Gln28 with residues Glu25, Phe26, and Leu27 showing the largest C^α^ differences (4.1, 4.8, and 2.8 Å, respectively).

In comparison with *h*ARH1, the distal ribose in the *Lch*ARH3 structure is rotated by ∼40° into the binding cleft, with contacts between the 2″ OH and Mg_I_ and the 3″ OH and Mg_II_ (Figures 1E, S5A, and S5C). Moreover, the absolutely conserved Gln137 interacts with the Mg_II_ coordinating residue Asp303, which fixes the position of the similarly conserved Ile260 and provides shielding for C4″ and C5″ of the distal ribose within the complex. Comparison of *Lch*ARH3 with the apo forms of *m*ARH3 (PDB: 2QTY) and *h*ARH3 (PDB: 2FOZ) showed no backbone or side-chain conformational changes in the active site.

We further analyzed our models for potential peptide/protein binding surfaces. The structure of *Lch*ARH3 presents an extended cleft running from the quasidomain A/B interface through the active site and along the quasidomain A/D interface (Figures 1D, 2K, and S6B). In particular, the A/B interface contains several highly conserved residues, including Tyr60, Arg100, and Gly101 (Figures S3 and S6B). While containing fewer conserved residues, comparison of *Lch*ARH3 with *m*ARH3 and *h*ARH3 shows that the cleft along the A/D interface is structurally conserved. In contrast, the model of *h*ARH1 shows no obvious binding clefts (Figures 2K and S6A), which suggests that endogenous substrate binding may require conformational changes. The A/B interface is closed due to the presence of helix α2, which allows interaction between Tyr19, Arg52, and Gly96 and is absent in *Lch*ARH3 (Figure S2A), as well as increased shielding of Mg_I_ due to the presence of Ala98 and Pro99 (the isostructural position is nearly exclusively occupied by a single glycine; Gly101 in *Lch*ARH3) (Figures S3 and S6A). On the other hand, the A/D interface presents similar features as in ARH3, indicating that it could transition into a more open conformation (Figure S6).

Comparison of *h*ARH1 and *Lch*ARH3 reveals a high degree of structural conservation within the active site, with the placement of the metal ions being absolutely isostructural (Figures 1E and 2A). However, several features make the active sites distinct: (1) Helix α2 in *h*ARH1 is replaced by a short loop region between helix α1 and α2 in *Lch*ARH3. (2) The interface between quasidomain A and D leading into the potential protein binding surface is opened up in ARH1 due to the relative rotation of the proximal ribose and the presence of the adenosine binding loop, which would clash with the position of loop 13 in ARH3 containing Ile260 (Figure 2A). (3) The absolutely conserved asparagine (Asn137 in *Lch*ARH3) is replaced by a similarly conserved cysteine (Cys129 in *h*ARH1) decreasing the polarity of the lower C4″/C5″ binding interface. Finally, (4) differences in the quasidomain B loop running along the edge of the binding cleft (loop 8 in *h*ARH1 and loop 5 in *Lch*ARH3, respectively) account for an increased shielding of Mg_I_ in *h*ARH1.

Together these data suggest that differences in ADPr recognition between ARH1 and ARH3 mostly result from differences in the adenosine pyrophosphate interaction. Although both proteins show few differences in the immediate active site, differences in the potential binding surfaces are likely to contribute to substrate specificity.

### Differential Inhibition of ARH1 and ARH3 by ADPr Analogues

In order to probe whether the differences between the active sites have an effect on substrate recognition, we used the ADPr analogues adenosine diphosphate (hydroxymethyl)pyrrolidinediol (ADP-HPD) and -monoalcohol (ADP-HPM). These compounds were originally designed as PARG inhibitors and contain pyrrolidine instead of the distal ribose as well as differing in the number of hydroxyl substituents (Figure 3A). The synthesis of ADP-HPM, which is not commercially available, was updated to include an improved pyrophosphate coupling method and streamlined protecting group strategy. The only reported synthesis of ADP-HPM was accomplished in nine steps (9% overall yield) and relied on poor-yielding pyrophosphate formation that required 5 days to complete (Koh et al., 2003). By implementing a rapid P(III)-P(V) coupling, we accomplished the synthesis in seven steps (37% overall yield) (Figure S7A; see STAR Methods for detailed synthesis description). This updated synthesis route is versatile and can be adapted to other ADPr analogues as demonstrated by the synthesis of ADP-HPD (Figure S7B and STAR Methods). Using *in vitro* generated substrates (arginine ADP-ribosylated whole cell lysate as a substrate for ARH1 and serine MARylated histone H3 peptide as a substrate for ARH3), we noticed a striking difference in the inhibitory potential of ADPr and its analogues for ARH1 and ARH3 (Figures 3B and 3C). While ADPr and ADP-HPD inhibited ARH3, both had only mild activity against *h*ARH1. ADP-HPM appeared to have only minor inhibitor potential against either of the enzymes. To further confirm these findings, we used the recently developed substrate analogue 4-(trifluoromethyl)umbelliferone ADPr (TFMU-ADPr) (Drown et al., 2018) and measured the fluorescence response in absence and presence of the inhibitors (Figure 3D and Table 3). While *h*ARH3 was inhibited by ADP-HPD and ADP-HPM (K_i_ of 0.58 ± 0.12 μM and 54.2 ± 4 μM, respectively), the K_i_ of *h*ARH1 was too high to be determined reliably. After the optimization of assay conditions (see STAR Methods), we were able to determine IC_50_ values for both *h*ARH1 and 3 (Figure 3E and Table 3). In line with the previous results, *h*ARH3 is most efficiently inhibited by ADP-HPD and to a lesser extent by ADPr and ADP-HPM (IC_50_ of 0.587 μM, 3.2 μM, and 480 μM, respectively). Due to the low inhibitory activity, IC_50_ values for *h*ARH1 could only be determined for ADPr and ADP-HPM (228 μM and 164 μM, respectively), whereas no inhibition by ADP-HPD could be observed under these conditions.

In order to elucidate further the selectivity of these inhibitors, we performed crystallization experiments with *h*ARH1 and *Lch*ARH3 in the presence of these ligands and solved the structures of the *h*ARH1:ADP-HPM, *Lch*ARH3:ADP-HPD, and *Lch*ARH3:ADP-HPM complexes (Table 1). In addition, our recent study showed that the inosine analogue of TFMU-ADPr, TFMU-IDPr, is a selective substrate for *h*ARH3 and that *in situ* generation of arginine-ADPr (Arg-ADPr) is a potent, cellular inhibitor of *h*ARH3 (Drown et al., 2018). To understand this selectivity, we also solved the structures of *Lch*ARH3:IDPr and *Lch*ARH3:Arg-ADPr (Table 1). The complexes show very good agreement with the models described above (Table S1). The most striking feature is the absence of Mg_II_ from the *h*ARH1:ADP-HPM and *Lch*ARH3:ADP-HPM complexes (Figures 3F, 3H, S2A, and S2B). The *h*ARH1:ADP-HPM complex shows an inversion of the pyrrolidine moiety caused by the in ring nitrogen, which clashes with the backbone nitrogen of Gly100. This orientates the C1″ position into the Mg_II_ binding site, expelling the Mg^2+^ ion, even though the crystallization condition contained 50 mM magnesium acetate. Coordination of ADP-HPM by *Lch*ARH3 leads also to loss of Mg_II_, albeit through a different mechanism. In fact, the pyrrolidine makes no direct contacts with Mg_I_, but contacts Asp63 via the in ring nitrogen and Glu33 via the 2″ OH. Due to this placement, a water (w518) has to occupy the Mg_II_ binding site in order to achieve octahedral coordination of Mg_I_, thus preventing the coordination of a second Mg^2+^ ion. In contrast, the orientation of ADP-HPD in the active site is similar to ADPr (Figures 1E, 3G, and S2A). The only differences are that the 2″ OH moiety of ADP-HPD is bridging Mg_I_ and Mg_II_ and a flip of C5″ rotates the pyrrolidine ring slightly away from the Mg^2+^ ions allowing for a more relaxed conformation. The same conformation is adopted by IDPr and Arg-ADPr with the difference that Arg-ADPr makes further contacts with the attached arginine side chain, specifically N^ɛ^ and N^η2^ interact with Glu33 and N^η1^ with Gly101, whereas a mixture of α- and β-anomers at the 1″ OH of IDPr is observed. Exchange of adenosine for inosine had no discernible influence on the placement of the purine ring within the structure. Also noteworthy, the β-form of the distal ribose is strained due to close contacts with Asp63 and Gly101 (Figures 3I, 3J, and S2A), which could assist in product release post-catalysis.

Together our data suggest that the differences in binding affinity result from a complex interplay of coordination losses (Mg_II_), as well as establishment of new contacts in the active site (e.g., the positively charged HPM nitrogen with Ser305 in the *h*ARH1 structure). Notwithstanding, certain trends become apparent: (1) The anomerization at the C1″ of the distal ribose appears to have a negative influence on the binding strength. Indeed, it was shown previously by us and others that both ARH1 and ARH3 can only hydrolyze α-linked ADP-ribose (Moss et al., 1986, Voorneveld et al., 2018). The structures now give rationale to this observation as the β-form would require rotating of the ribose ring plane, which in turn may influence the coordination with Mg_I_ and Mg_II_. (2) The coordination of Mg_II_ is relatively weak and displacement can be favorable for ligand binding. (3) The affinity of ARH1 for the tested compounds is low, which may indicate that further enzyme-substrate contacts are required for the formation of a catalytic complex. From these data we conclude that even though ARH1 and ARH3 have strikingly similar distal ribose binding sites, it is possible to design chemical probes that can distinguish between the two enzymes.

## Discussion

In the present study, we describe the ligand-bound structures of the ARH family members ARH1 and ARH3. Our data indicate that despite the highly specialized reactions they catalyze, with ARH1 hydrolyzing ADP-ribosyl linkages to arginine and ARH3 to serine residues (Fontana et al., 2017, Moss et al., 1988), these enzymes are very similar, in particular with respect to their active sites. The structures presented here provide critical insights into their mode of enzyme-ligand interaction, help us to understand the differences in their catalytic behavior, and present a useful tool for targeted drug design.

One major distinction between the structures of ARH1 and ARH3 is the coordination of the adenosine pyrophosphate moieties, which has direct consequences for their substrate interactions. The ability of ARH3, but not ARH1, to degrade PAR chains (Niere et al., 2012, Oka et al., 2006) has implicated it in PARP1-mediated cell death, where it guards against cell death through its ability to degrade PAR chains (David et al., 2009, Mashimo et al., 2013). Our structures suggest that ARH3 is able to bind and cleave PAR chains both in an *endo* and *exo* manner, thus allowing the degradation of both attached and free chains. This is due to the orientation of the proximal ribose, which exposes both the 2′ and 3′ OH toward the enzyme surface, with hardly any limitations to the attachment of further ADPr units. In contrast, the proximal ribose in ARH1 is coordinated by the rigid adenosine binding loop (loop 16). The resulting orientation aids selectivity toward MARylated substrates, which aligns well with previous reports that ARTCs are mono-specific transferases (Corda and Di Girolamo, 2003).

**Figure 4:**
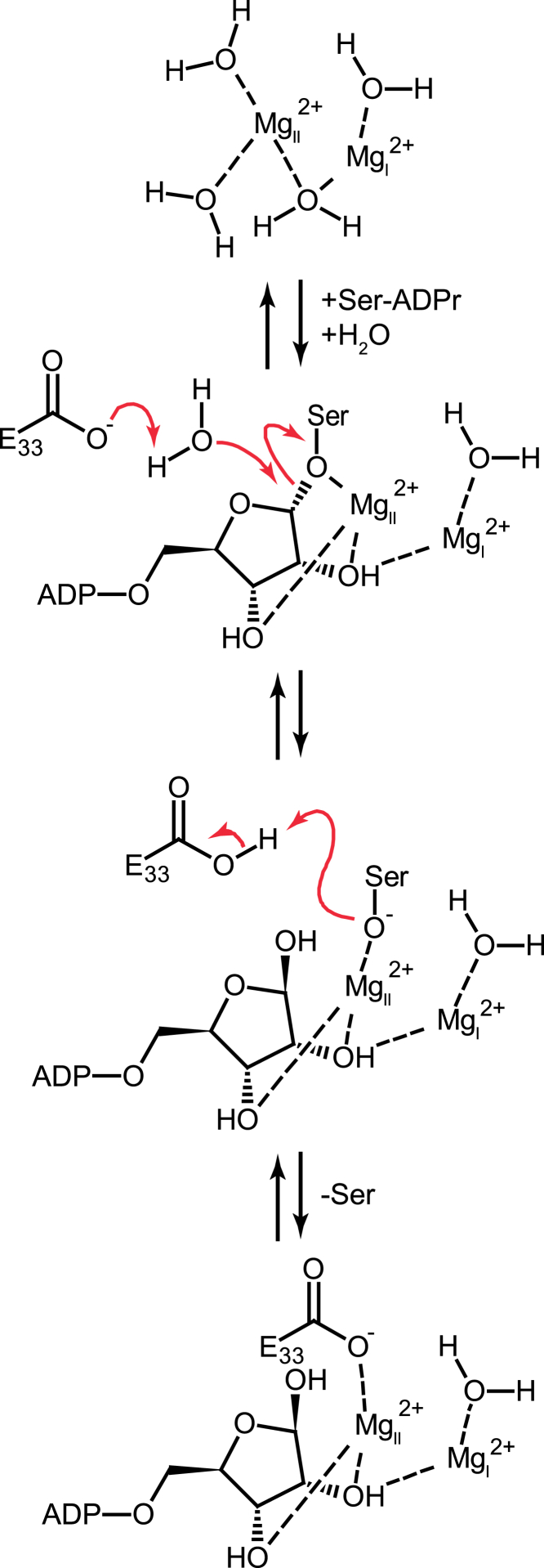
Proposed Reaction Mechanism for ARH3

Given the different substrate specificities, ARH1 preferentially cleaves *N*-glycosidic and ARH3 *O*-glycosidic bonds, the similarities between the active centers is surprising. All major contacts needed for the orientation of the distal ribose appear to be conserved, which includes the coordinated Mg^2+^ ions as well as interaction with Ala98 and Asp302 (*h*ARH1) and Gly101 and Asp303 (*Lch*ARH3), respectively. However, the 1″ OH in the *Lch*ARH3:ADPr complex appears to take part in the second coordination sphere of Mg_II_, whereas this is not the case in the *h*ARH1:ADPr complex. In the latter, the ADPr takes on a more relaxed conformation, which is mimicked in the *Lch*ARH3:Arg-ADPr and :ADP-HPD complexes, thus suggesting that an α-linked oxygen draws the ribose ring closer to the magnesium ion. A second striking aspect of the Mg_II_ coordination is the placement of an absolutely conserved glutamic acid crucial for full catalytic activity (Glu25 in *h*ARH1 and Glu33 in *Lch*ARH3, Figures S3 and 2C). While this glutamate coordinates Mg_II_ in the earlier reported human apo structure (PDB: 2FOZ) (Mueller-Dieckmann et al., 2006), our data show that it is not strictly necessary for Mg^2+^ coordination (Figures 1E and 2A). During revision, structures of the *h*ARH3:ADPr complex became available, highlighting an even greater degree of flexibility in glutamic acid positioning (Figure S1C) (Pourfarjam et al., 2018, Wang et al., 2018). While the mode of ADPr coordination between *h*ARH3 and *Lch*ARH3 is very similar overall, a key differences is the coordination of 2″OH at the distal ribose. In our structures, this moiety is either coordinating Mg_I_ (ADPr structure) or bridging between the Mg^2+^ ions (ADP-HPD, Arg-ADPr, and IDPr). In contrast, the distal ribose of the *h*ARH3:ADPr complex is rotated away from the binuclear metal center allowing the bridging water of the apo form to remain albeit in very close proximity (2.14 ± 0.11 Å) to the 2″ OH group. It is interesting to note that in *h*ARH1 Glu25 is part of the short helix α2, which is replaced by a short loop with Glu33 positioned at the end of helix α1 in *Lch*ARH3. This exchange imposes different constraints on the flexibilities of these residues during catalysis. Finally, the structure of the *Lch*ARH3:ADPr complex from the initial crystal system prior to optimization (PDB: 6G1Q, this study) suggests that while ligand binding is possible in the absence of Mg_II_, the specific arrangement of ADPr cannot be maintained in the active site. The low K_d_ of magnesium on its own (∼5.4 mM for *h*ARH3, ∼2.3 mM for *Lch*ARH3; Figures 2F and 2G), together with the apparent low concentration needed to restore activity (∼50 μM, Figure S2C), suggests that the substrate contributes to the stabilization of the binuclear metal center. This dissociative tendency is highlighted by the structures of *h*ARH1:ADP-HPM and *Lch*ARH3:ADP-HPM, which lack Mg_II_ in their active site, despite the presence of magnesium in the final crystallization condition (50 mM and 10 mM, respectively). The inability to coordinate Mg_II_ could account for the dramatic drop in K_i_ and IC_50_ observed for ARH3 (Table 3). This mode of inhibition stands in contrast to the structural studies on ADP-HPD as a PARG inhibitor (Kim et al., 2012, Lambrecht et al., 2015, Tucker et al., 2012). In PARG, the substrate is bound in a strained conformation due to the presence of a conserved phenylalanine (Phe875 in human PARG), which forces the distal ribose, in particular the linkage 1″ carbon, toward the catalytic loop. This conformation is further stabilized by extensive contacts between the Phe875 containing loop and the pyrophosphate moiety. Together this leads to a closed conformation and restricts the possibility of movement in the distal ribose. Consequently, comparison of ADPr (PDB: 4B1H), ADP-HPD (PDB: 4B1J), and ADP-HPM (this study; Figure S2D, Tables 1and S1) -bound PARG structures revealed little difference in the mode of binding (Figures S7A and S7C). The strained conformation, however, brings the ribose ring oxygen into the proximity of the P^α^ phosphate, which could stabilize the oxocarbenium reaction intermediate and explain the higher potency of ADP-HPD, which has a positively charged pyrrolidine nitrogen, in comparison with ADPr. In contrast, both ARH1 and ARH3 complexes show a relaxed ADPr conformation (Figures 1E, S2A, and S2B), which precludes a theoretical oxocarbenium-phosphate interaction. Together with the few restrictions on movement of the distal ribose in the active site, these findings offer several potential explanations: (1) formation of a short-lived oxocarbenium intermediate, (2) movement of the substrate during the catalytic mechanism, or (3) a mechanism that does not involve the formation of an oxocarbenium intermediate. While we cannot rule out (1) or (2), our findings and earlier reported data support a mechanism in which the distal ribose is orientated in the active site via interactions with both Mg^2+^ ions (Figure 4). In this strained conformation, the scissile bond becomes susceptible to a base-mediated S_N_2 attack of a nucleophilic water from the second coordination sphere. The intermediate serine oxyanion is stabilized by the interaction with Mg_II_, while the distal ribose obtains a more relaxed conformation and leaves the immediate catalytic site. The serine is subsequently protonated and released. Given the importance of Glu33 for the catalytic mechanism, its high flexibility (Figure S1C) and no alternative reversible protonatable residues in the catalytic center, we propose that it acts as acid/base in the reaction cycle and assists the serine release by coordinating Mg_II_ after the protonation step.

Our structures also give important insights into the development of selective inhibitors with three hotspots for modification: (1) As demonstrated with the development of a selective TFMU-IDPr substrate, selectivity can be achieved by using the differences in adenosine ribose binding. The coordination of the IDPr purine in *Lch*ARH3 is isostructural to the one of ADPr, suggesting that exchanges at the C6 position (and verisimilar the pyrimidine positions 1 to 3) are well tolerated by ARH3. In contrast, in PARG, the C6-linked amino group makes an electrostatic contact with Glu727 and water molecules. The former contact is not possible with an inosine ring, whereas the effect on water coordination is less clear, but the relative buried coordination of the purine ring indicates that an alteration of the water network could be detrimental for binding. The coordination of ADPr and IDPr in the *Lch*ARH3 structures suggests that substitution at the pyrimidine should be tolerated well by ARH3, whereas this position is more shielded in both PARG and ARH1. (2) Comparison of the potency of ADP-HPD and -HPM as ARH3 inhibitors as well as our differential scanning fluorimetry (DSF) data suggests that the stabilization of the binuclear metal center could increase the efficiency of inhibitors. Alternatively, displacement of the labile Mg_II_ with favorable contacts could have similar effects. (3) The *Lch*ARH3:Arg-ADPr complex suggests that further functionalization, here the attachment of a guanidino moiety to the C1″ position, is responsible for the improved inhibition compared with ADPr and ADP-HPD. The guanidino moiety makes several contacts with the catalytic Glu33, which stabilizes the ARH3:Arg-ADPr complex and limits the flexibility of the Glu33 side chain required for catalysis. Given the importance of ARH1 as a tumor suppressor (Kato et al., 2011) and its involvement in the defense against bacterial toxins (Kato et al., 2007), as well as the emerging role of ARH3 in the DDR and neurodegenerative disorders (Ghosh et al., 2018, Palazzo et al., 2018), such pharmacological tools would be highly desirable, not only to further elucidate their physiological functions, but also for their potential as therapeutic targets. For example, PARG is being investigated as a chemotherapeutic target and promising inhibitors for it were recently discovered (Gravells et al., 2017, James et al., 2016), but the overlap in function between PARG and ARH3 remains to be elucidated. PARG activity appears to decrease with decreasing chain length and only low activity against oligomers of fewer than five units could be detected (Barkauskaite et al., 2013, Hatakeyama et al., 1986, Uchida et al., 1993). This opens up the possibility that ARH3 rather than PARG hydrolyzes these short chains. Furthermore, the ability of ARH3 to degrade PAR chains of any length may allow ARH3 to partially compensate for the effect of PARG inhibition, thus making it an interesting target to further increase the effect of PARG inhibition.

## Significance

**ADP-ribosylation is an important process for cell homeostasis as it participates in the regulation of a wide variety of cellular processes, including DNA damage repair, aging, immunity, bacterial metabolism, and many others. (ADP-ribosyl)hydrolases (ARHs) catalyze the removal of specific ADP-ribosyl modifications from proteins with arginine- or serine-ADP-ribose linkages. Whereas ARH1 is specific for the *N*-glycolytic linkage of mono(ADP-ribosylated) arginines, ARH3 specifically cleaves the *O*-glycolytic linkage of mono(ADP-ribosylated) serine and, at least *in vitro*, other linkages as well, such as those in poly(ADP-ribose) chains. Our kinetic and structural analyses of ARH1 and ARH3 point out their catalytically important residues and reveal the structural basis for the differences between the substrate specificity and inhibitor affinity of these enzymes. These findings will provide further opportunities to design new targeted ADP-ribose derivatives to modulate the activities of other members of the ARH family. The therapeutic potential of such new molecules shows great promise and is worth exploring since ARH1 is an important tumor suppressor and involved in the defense against bacterial toxins, and ARH3 has an emerging role in the DNA damage response.**

## STAR★Methods

### Key Resources Table

**Table:** 

REAGENT or RESOURCE	SOURCE	IDENTIFIER
**Antibodies**		

Mouse monoclonal hexahistindine antibody	Clontech	631212; RRID: AB_2721905
horseradish peroxidase-conjugated streptavidin	Pierce	21130
Polyclonal goat anti-mouse immunoglobulins/HRP	Dako	P0447; RRID: AB_2617137

**Bacterial Strains**		

*E. coli* JM109 Competent Cells	Promega	L2005
*E. coli* Rosetta (DE3) Competent Cells	Novagen (Merck)	0954-3CN

**Chemicals, Peptides, and Recombinant Proteins**		

ADP-ribose	Sigma-Aldrich	A0752
ADP-HPM	This study	n/a
ADP-HPD	Calbiochem (Merck)/ this study	118415 / n/a
IDPr	This study	n/a
Arg-ADPr	(Drown et al., 2018)	n/a
TFMU-ADPr	(Drown et al., 2018)	n/a
TFMU-IDPr	(Drown et al., 2018)	n/a
^32^P-NAD^+^	PerkinElmer	NEG023X500UC
Trans-4-hydroxyproline methyl ester	Sigma-Aldrich	30681
Diisopropylethylamine	Sigma-Aldrich	D125806
FmocCl	Sigma-Aldrich	160512
Tert-butyldimethylsilyl chloride	Oakwood Chemical	003869
Imidazole	Sigma-Aldrich	I202
Lithium borohydride	Sigma-Aldrich	62460
*N*,*N*,*N’*,*N’*-tetraisopropyl 9-methylfluorenylphosphoramidite	(Hofer et al., 2015)	n/a
Tetrazole	Sigma-Aldrich	88185
Tert-butylhydrogenperoxide	Sigma-Aldrich	416665
Trifluoroacetic acid	Sigma-Aldrich	T6508
4,5-Dicyanoimidazole	Sigma-Aldrich	554030
Martin’s Sulfurane Dehydrating Agent	Sigma-Aldrich	428841
Osmium tetroxide	Sigma-Aldrich	201030
tert-Butyldimethylsilyl trifluoromethanesulfonate	Sigma-Aldrich	226149
*N*,*N*-dimethylethylamine	Sigma-Aldrich	652571
2,6-lutidine	Sigma-Aldrich	L3900
4-Methylmorpholine *N*-oxide	Sigma-Aldrich	224286
AMP	Sigma-Aldrich	A2252
Silicagel 60M	Macherey-Nagel	815381.25P
Dowex 50WX2 hydrogen form 50-100 mesh	Oakwood Chemical	099513
Dowex 50WX8	Sigma-Aldrich	217514
SYPRO™ Orange (5000X in DMSO)	ThermoFisher Scientific	S6650
Lithium chloride	Sigma-Aldrich	310468
Magnesium chloride hexahydrate	Sigma-Aldrich	M2670
Calcium chloride dehydrate	Fluka	223506
Chromium(III) chloride hexahydrate	Sigma-Aldrich	27096
Manganese(II) chloride tetrahydrate	Sigma-Aldrich	221279
Cobalt(II) chloride hexahydrate	Sigma-Aldrich	C 2644
Nickel(II) chloride hexahydrate	Alfa Aesar	A14366
Copper(II) chloride	Fisher Scientific	C/7920/48
Zinc chloride	Sigma-Aldrich	31650

**Critical Commercial Assays**		

ProPlex Screen HT-96	Molecular Dimensions	MD1-42
Structure Screen 1 + 2 HT-96	Molecular Dimensions	MD1-30

**Deposited Data**		

*h*ARH3 apo structure	(Mueller-Dieckmann et al., 2006)	PDB: 2FOZ
*m*ARH3 apo structure	(Mueller-Dieckmann et al., 2008)	PDB: 2QTY
*h*ARH1 apo structure	deposited	PDB: 3HFW
*h*ARH3:ADPr structure	(Wang et al., 2018)	PDB: 5ZQY
*h*ARH3:ADPr structure	(Pourfarjam et al., 2018)	PDB: 6D36
*h*ARH1:ADPr structure	This paper	PDB: 6G28
*h*ARH1:ADP-HPM-structure	This paper	PDB: 6G2A
*Lch*ARH3 apo structure	This paper	PDB: 6G1P
*Lch*Arh3:ADPr structure (one Mg^2+^ ion)	This paper	PDB: 6G1Q
*Lch*Arh3:ADPr structure (two Mg^2+^ ions)	This paper	PDB: 6HGZ
*Lch*Arh3:ADP-HPD structure	This paper	PDB: 6HH3
*Lch*Arh3:ADP-HPM structure	This paper	PDB: 6HH5
*Lch*Arh3:IDPr structure	This paper	PDB: 6HOZ
*Lch*Arh3:Arg-ADPr structure	This paper	PDB: 6HH4
PARG:ADP-HPM structure	This paper	PDB: 6HH6

**Experimental Models: Cell Lines**		

HeLa (Human adenocarcinoma)	ATCC	ATCC CCL-2

**Recombinant DNA**		

pET-41a(+)	Novagen (Merck)	70556-3
pET-9H_3_	(Rack et al., 2015)	n/a
pDEST17-*h*ARH1	(Fontana et al., 2017)	n/a
pASK60-OmpA-mARTC2.2	(Mueller-Dieckmann et al., 2002)	n/a
pDEST17-HPF1	(Gibbs-Seymour et al., 2016)	n/a
pET-28a-PARP1	(Langelier et al., 2011)	n/a
*Pma*ARH3/*Xtr*ARH3/*Lch*ARH3	GeneArt™	Custom gene synthesis

**Software and Algorithms**		

CCP4i2	(Potterton et al., 2018)	http://www.ccp4.ac.uk
PHENIX	(Adams et al., 2010)	http://www.phenix-online.org
COOT	(Emsley et al., 2010)	http://www2.mrc-lmb.cam.ac.uk/personal/pemsley/coot
JalView (v 2.10)	(Waterhouse et al., 2009)	http://www.jalview.org
PyMol (v 1.8)	Schrödinger, LLC	http://www.pymol.org
Aline (v 1.0)	(Bond and Schuttelkopf, 2009)	http://www.bondxray.org/software/aline.html
CheckMyMetal	(Zheng et al., 2014, Zheng et al., 2017)	https://csgid.org/csgid/metal_sites
Xia2	(Winter et al., 2013)	https://xia2.github.io
PHASER	(Storoni et al., 2004)	http://www.phaser.cimr.cam.ac.uk
LigPlot^+^ (v 1.4)	(Laskowski and Swindells, 2011)	https://www.ebi.ac.uk/thornton-srv/software/LigPlus
Prism 6	GraphPad	n/a
SoftMax Pro (v 6.4)	Molecular Devices	Build 204720

**Other**		

Amicon Ultracel-3k	EMD Millipore	Cat# UFC800324
ACCQPrep	Teledyne Isco	HP125UV
Luna C18 5 μm 21.2x150mm	Phenomenex	00F-4252-P0-AX
CombiFlash Rf+	Teledyne Isco	68-5230-022
SpectraMax Multi-mode Microplate Reader	Molecular Devices	M3
RediSep Rf C18 Gold 5.5g	Teledyne Isco	69-2203-328
RediSep Rf C18 Gold 150g	Teledyne Isco	69-2203-338
GSTrap 4B (1 mL)	GE Healthcare	29048609
HisTrap HP (5 mL)	GE Healthcare	17524801
HiTrap Q (1 mL)	GE Healthcare	29051325
HiLoad 16/600 Superdex 75 pg	GE Healthcare	28989333
Ni-NTA Agarose	Jena Bioscience	AC-501-100
Seed Beads	Hampton Research	HR2-320
MRC crystallization plate (2 drop, 96 well)	Molecular Dimensions	MD11-00
MicroAmp^®^ fast 96-well reaction plates	Life Technologies	4346907

### Contact for Reagent and Resources Sharing

Further information and requests for resources and reagents should be directed to and will be fulfilled by the Lead Contact, Ivan Ahel (ivan.ahel@path.ox.ac.ac).

### Experimental Model and Subject Details

*Escherichia coli* JM109 and Rosetta (DE3) cells were grown in LB medium supplemented with 2 mM MgSO_4_ and antibiotics appropriate for each expression plasmid at 37°C.

Human HeLa cell (Female, 31 years old) were cultured in DMEM supplemented with 10% FBS and penicillin-streptomycin (100 U/mL) at 37°C in humidified atmosphere containing 5% CO_2_.

### Method Details

#### Plasmid Construction

The coding sequence of *h*ARH1 was amplified from a pDEST17-ARH1 vector (Fontana et al., 2017) and cloned into pET-41a(+) via the NcoI/XhoI restriction sites introducing a short N-terminal His-tag. Sequences of *Pma*ARH3 (GenBank: XP_015504659; aa residues 19-370), *Xtr*ARH3 (GenBank: CAJ81573.1; full length) and *Lch*ARH3 (GenBank: XP_005988572; aa residues 10-362) were codon optimized for expression in *E. coli*, gene synthesized (GeneArt™; Thermo Fisher Scientific) and cloned into pET-9H_3_ (Rack et al., 2015) using the vector’s NcoI/BamHI sites. Expression vectors for *h*ARH3, HPF1, PARP1, PARG and *m*ARTC2.2 were described earlier (Fontana et al., 2017, Gibbs-Seymour et al., 2016, Lambrecht et al., 2015, Langelier et al., 2011, Mueller-Dieckmann et al., 2002, Tucker et al., 2012). All indicated mutations were introduced via PCR based site-directed mutagenesis.

#### Sequence Alignments

The multiple sequence alignment of ARH1 and ARH3 proteins was generated using JalView 2.8.0b1 (Waterhouse et al., 2009) and the MAFFT L-INS-I algorithm implemented therein (Katoh and Toh, 2010). Alignment representations were created with ALINE (Bond and Schuttelkopf, 2009). Sequence details and accession numbers are given in Table S3.

#### Protein Expression and Purification

##### For Biochemistry

Expression of recombinant proteins in Rosetta (DE3) cells was induced at OD_600_ 0.6 with 0.4 mM IPTG, cells were grown overnight at 290 K and harvested by centrifugation. Recombinant His-tagged proteins were purified at 277 K by Ni^2+^-NTA chromatography (Jena Bioscience) according to the manufacturer’s protocol using the following buffers: all buffers contained 50 mM TrisHCl (pH 8) and 500 mM NaCl; additionally, the lysis buffer contained 25 mM, the washing buffer 40 mM and the elution buffer 500 mM imidazole. For purification of the ARH proteins all buffers also contained 10 mM MgCl_2_. All proteins were dialysed overnight against 50 mM TrisHCl (pH 8), 200 mM NaCl, 1 mM DTT and 5% (v/v) glycerol. Purity of the protein preparations was assessed using SDS-PAGE and Coomassie Brilliant Blue (CBB) staining (Figure S2E).

##### For Differential Scanning Fluorimetry (DSF)

*Lch*ARH3 WT was purified as described above and dialysed against 50 mM TrisHCl (pH 8), 200 mM NaCl, 20 mM EDTA, 1 mM DTT and 5% (v/v) glycerol. EDTA was removed by dialysing twice against 50 mM TrisHCl (pH 8), 200 mM NaCl, 1 mM DTT and 5% (v/v) glycerol. Each dialysis step was carried out with a minimum 1:1,000 ratio of sample to buffer. Only samples with purity >90% (as assessed by SDS-PAGE) were used for subsequent DSF experiments.

##### For Crystallisation

*h*ARH1 underwent affinity purification over a HisTrap HP column (GE Healthcare), followed by anion exchange chromatography using a HiTrap Q HP column (GE Healthcare) using buffer A (50 mM TrisHCl [pH 8]) and B (50 mM TrisHCl [pH 8], 1 M NaCl) and a gradient elution of 3-100% B and size exclusion chromatography using a HiLoad Superdex 75 pg column (GE Healthcare) with 10 mM Bis-Tris propane (pH 7.6), 100 mM NaCl and 1 mM DTT as elution buffer.

*Lch*ARH3 was affinity purified over a HisTrap HP column, followed by dialysis against 50 mM TrisHCl (pH 8), 500 mM NaCl, 20 mM imidazole and 1 mM DTT in presence of HRV3C protease for proteolytic cleavage of the His-tag. Removal of the uncleaved protein was achieved by rebinding to a HisTrap HP column and the protease was removed by binding to a GSTrap 4B column (GE Healthcare). The final step involved size exclusion chromatography using a HiLoad Superdex 75 pg column with 10 mM PIPES (pH 7), 100 mM NaCl and 1 mM DTT as elution buffer.

PARG was expressed and purified as described earlier (Lambrecht et al., 2015, Tucker et al., 2012). Briefly, PARG pellets were resuspended in homogenisation buffer (50 mM TrisHCl [pH 8], 400 mM NaCl, 5 mM β-mercaptoethanol, 5 mM imidazole, 0.3 mg/mL lysozyme, 2.5 U/mL Benzonase (Novagen) and cOmplete™ EDTA-free protease inhibitor (Roche)) and homogenized. The cleared lysate underwent affinity purification over a HisTrap HP column (GE Healthcare), the affinity tag was cleaved using TEV protease and separated from uncleaved protein by subtractive IMAC over a HisTrap HP column (GE Healthcare). The cleaved protein underwent size exclusion chromatography using a HiLoad Superdex 200 pg column with 50 mM HEPES (pH 7), 150 mM NaCl and 2 mM DTT as elution buffer.

Proteins were concentrated using Vivaspin 20 columns (GE Healthcare).

#### Sample Analysis and Antibodies

Reactions for analysis were stopped by adding LDS sample buffer and incubation for 5 min at 90°C. Subsequently the samples were resolved by SDS-PAGE and either vacuum dried for autoradiography or transferred onto a nitrocellulose membrane. Immunoblot analyses were carried out using primary and secondary antibodies as indicated. Proteins were detected by enhanced chemiluminescence (Pierce).

#### (ADP-ribosyl)hydrolase Activity Assays

##### ARH1: Removal of Arg-ADP-ribosylation from HeLa Cell Extracts

*In vitro* modification of proteins from HeLa cell extracts by *m*ARTC2.2 recombinant protein was performed, with minor modifications, as described earlier (Palazzo et al., 2016). Briefly, cells were grown to confluence on two 10-cm dishes, washed thrice with 50 mM TrisHCl (pH 7.5) and 200 mM NaCl and lysed in 800 μL of 50 mM Tris-HCl pH 7.5, 150 mM NaCl, 0.5% Triton X-100, 0.2 mM DTT and 1 μM olaparib at 4°C supplemented with cOmplete™ EDTA-free protease inhibitor (Roche). The cell extract was clarified by centrifugation. 12 uL of extract per reaction were supplemented with 15 mM MgCl_2_, 1 μM *m*ARTC2.2 and 1 μCi ^32^P-NAD^+^ and incubated for 15 min at 30°C. Lysates were further incubated in presence of hydrolases for 45 min at 30°C. Reactions were stopped by addition of LDS sample buffer (Life Technologies) and incubation at 95°C for 3 min. Samples were then analysed by SDS-PAGE and autoradiography. For inhibitor study the *h*ARH1 was pre-incubated with indicated amount of inhibitor for 5 min at RT.

##### ARH3: Degradation of PAR and De-modification of Ser-ADP-ribosyl H3 Peptide

ARH3 activity assays were performed essentially as described (Fontana et al., 2017). Briefly, H3 peptide (aa 1-20, biotinylated) was modified by incubation with 0.5 μM PARP1, 1 μM HPF1 and activated DNA (Trevigen) in assay buffer (50 mM TrisHCl [pH 8], 200 mM NaCl, 2 mM MgCl_2_, 1 mM DTT, 10 μM NAD^+^ and 1 μCi ^32^P-NAD^+^). Reactions were incubated for 30 min at 30°C and stopped by addition of 1 μM olaparib. Reaction were further incubated in presence of 1 μM hydrolase for 1 h at 30°C. Reactions were stopped by addition of LDS sample buffer (Life Technologies) and incubation at 95°C for 3 min. Samples were then analysed by SDS-PAGE, immunoblot and autoradiography. For inhibitor study the ARH3 was pre-incubated with indicated amount of inhibitor for 5 min at RT.

#### DSF

Assays were performed essentially as described earlier (Vivoli et al., 2014). Briefly, 10 μM *Lch*ARH3 wt EDTA-treated, 10x SYPRO^®^ Orange (ThermoFisher Scientific) and indicated amounts of metal salts in assay buffer (50 mM TrisHCl [pH 8], 200 mM NaCl and 1 mM DTT) were thermal denaturated in MicroAmp^®^ Fast plates (Life Technologies) in a StepOne qPCR instrument (ThermoFisher Scientific). The initial temperature of 24°C was held for 2.5 min, followed by a ramp to 97.5°C at 0.5°C/45 sec and then 2 min pause at 98°C. Curves were fit using GraphPad Prism 6. For analysis recorded data were cropped two data points after recorded fluorescence signal maximum and the resulting curves fitted using the Boltzman equation

$$F=BOTTOM+\frac{\left(TOP-BOTTOM\right)}{1+e^{\left(\frac{T_{M}-T}{SLOPE}\right)}}$$

with T_M_, melting temperature; T, temperature; SLOPE, steepness of curve. For the inference of the binding constant the determined T_M_’s were plotted and analysed using a single binding site model:

$$T_{M}=BOTTOM+\left(\left(TOP-BOTTOM\right)*\left(1-\left(\frac{P-K_{d}-M+\sqrt{{\left(P+M+K_{d}\right)}^{2}*\left(4*P*M\right)}}{2P}\right)\right)\right)$$

with P, protein concentration; M, metal salt concentration; K_d_ dissociation constant.

For the initial metal salt screen 100 μM of indicated salt were added to the sample, whereas for the K_d_ determination 16-point dilution series were added: steps are 2.5-fold dilutions with MgCl_2_ starting at 500 mM, CaCl_2_ at 300 mM and MnCl_2_ at 50 mM.

#### Enzyme Kinetics and Inhibition Assay

Reactions were performed as described in (Drown et al., 2018). Briefly, reactions were carried out in 384-well plates using 50 mM Na_2_HPO_4_ (pH 7.4), 10 mM MgCl_2_, 5 mM DTT and indicated amounts of TFMU-ADPr as substrate. After shaking for 5 s, fluorescence was recorded at 5 s intervals for 15 min on a Molecular Devices SpectraMax M3 microplate reader (reader settings: λ_Ex_ 385 nm, λ_Em_ 502 nm, λ_cutoff_ 495 nm, 6 reads/well, low gain). Initial reaction rates were determined by fitting the linear portions of reaction progress curves. Initial rates were plotted against substrate concentration and fit to the Michaelis-Menton equation using a non-linear curve-fitting algorithm in GraphPad Prism 6.

For inhibitor studies enzymes and inhibitor were pre-incubated for 5 min at RT before addition of substrate. Percent inhibition was calculated with the no enzyme and no inhibitor reactions as positive and negative controls, respectively. Dose-response curves were fit using GraphPad Prism 6.

#### Crystallisation

*h*ARH1 for crystallisation was expressed as described above and purified protein concentrated to 500 μM (∼20.3 mg/mL). Initial *h*ARH1 crystallisation condition for protein supplemented with 2.5 mM ADPr were identified using the ProPlex matrix screen (Molecular Dimensions). Reproducible crystals grew under various condition using ADPr containing crystals as seed stock as follows: crystals were grown at 292 K by sitting-drop vapour diffusion in MRC 96 well plates (Molecular Dimensions) by mixing 200 nL purified protein supplemented with 2.5 mM ligand with 50 nL seed stock and 250 nL of precipitant solution. Seed stock was prepared using Seed Bead™ (Hampton Research) with several crystals and mother liquor to 50 μL as stabilizing solution. For ADPr containing crystals the precipitant solution consisted of 200 mM magnesium formate, 20% (w/v) PEG3350 and for ADP-HPM crystals of 100 mM magnesium acetate, 100 mM MOPS (pH 7.5) and 12% (w/v) PEG8000. The crystals were cryoprotected by dipping them into a solution of 18% (v/v) glycerol in precipitant solution and vitrified by submersion in liquid nitrogen.

*Lch*ARH3 for crystallisation was expressed as described above and purified protein concentrated to 300 μM (∼11.5 mg/mL). Initial *Lch*ARH3 crystallisation condition for the unligated protein were identified using the Structure Screen 1 & 2 HT-96 screen (Molecular Dimensions). Reproducible crystals for structure determination and soaking experiments were grown at 292 K by the sitting-drop vapour diffusion method in MRC 96 well plates (Molecular Dimensions) in 100 mM sodium citrate (pH 4.6–5.6), 21-27% (w/v) PEG4000 and 200 mM ammonium acetate. For determination of the ADPr complex with the initial crystal system prior to optimisation, the crystals were soak 3 h with 10 mM ADPr. Apo and ADPr complex crystals were vitrified by transfer into mother liquor supplemented with 16 % (v/v) ethylene glycol for 5 sec prior to submersion in liquid nitrogen. Subsequently, crystal conditions were optimized by addition of 10 mM MgCl_2_ and 10 % (v/v) glycerol to the protein sample prior to set-up of crystal conditions. For ADPr and analogue complexes, apo crystals were soaked for 3 h with 10 mM ADPr, 10 mM ADP-HPD, 10 mM IDPr and 10 mM Arg-ADPr as well as for 74 h with 5 mM ADP-HPM in mother liquor containing 10 mM MgCl_2_ and 6 % (v/v) glycerol. All optimized crystals were vitrified by transfer into mother liquor supplemented with 20% glycerol for 5 sec prior to submersion in liquid nitrogen.

PARG was crystallised as described earlier (Lambrecht et al., 2015, Tucker et al., 2012). Briefly, PARG was concentrated to 7.5 mg/mL in SEC buffer, supplemented with 1 mM ADP-HPM and crystals were grown at 292 K by sitting drop vapour diffusion by mixing in MRC 96 well plates (Molecular Dimensions) in 200 mM ammonium sulphate, 100 mM PCTP (pH 7.5) and 19% (w/v) PEG3350. Crystals were cryoprotected in a solution of 20% (v/v) glycerol in mother liquor and vitrified in liquid nitrogen.

#### X-ray Data Collection, Processing and Refinement

X-ray diffraction data were collected using synchrotron radiation at Diamond Light Source (Rutherford Appleton Laboratory, Harwell, UK) (Table 1) and processed using Xia2 (Winter et al., 2013). *h*ARH1 structure was solved via molecular replacement using PHASER (Storoni et al., 2004) with a model produced from human ARH1 protein (PDB 3HFW), *Lch*ARH1 structure was solved with a model derived from human ARH3 (PDB 2FOZ) and PARG structure was solved with a model derived from human PARG (PDB 5A7R). Model building for all structures was carried out in COOT (Emsley et al., 2010) and real space refinement with REFMAC5 (Murshudov et al., 1997) within CCP4i2 (Potterton et al., 2018). Datasets from the initial crystal system prior to optimisation had higher R-factors than expected post-refinement. Analysis of the collected data indicated that the datasets for *h*ARH1 and *Lch*ARH3 suffered from translational pseudo-symmetry (TPS). Improvements of the crystallisation conditions for *Lch*ARH3 appears to have reduced the amount of TPS, which coincided with improved R-factors. Metal coordination was validated using the CheckMyMetal (CMM) server (Table S2) (https://csgid.org/csgid/metal_sites/) (Zheng et al., 2014, Zheng et al., 2017), structural figures were prepared using PyMOL (Molecular Graphics System, Version 1.8 Schrödinger, LLC), Protein-ligand interaction figures were produced with LigPlot^+^ (Laskowski and Swindells, 2011) and polder OMIT maps were calculated using the *PHENIX* implementation of the algorithm (Adams et al., 2010, Liebschner et al., 2017).

#### Synthesis of IDP-ribose

TFMU-IDPr (4.8 mg, 6.0 μmol) was dissolved in 15 mL ARH3 reaction buffer (50 mM Na_2_HPO_4_, 10 mM MgCl_2_, 5 mM DTT, pH 7.4) and *h*ARH3 was added to a final concentration of 1 μM. Mixture was incubated at 37°C for 12 h. Protein was removed from reaction mixture by 3 kDa MWCO centrifuge filtration and filtrate was lyophilized. The solid was redissolved in solvent A (20 mM Et_3_N⋅HOAc (pH 7.2)) and subjected to ion-pairing preparative HPLC using Luna C18 21.5x150mm column with solvent A to solvent B (acetonitrile) gradient of A:B (20 mL/min): 98:2, 0 min; 98:2, 2 min; 75:25, 16 min; 75:25, 23 min; 40:60, 27 min, 40:60, 30 min. Fractions were analysed by LCMS to identify which contain IDPr. Fractions containing IDPr were lyophilized and redissolved in H_2_O. Triethylammonium cation was exchanged for ammonium by passing through Dowex 50WX2 (ammonium form). Flow through was lyophilized to give a pale yellow solid (3.2 mg, 89%).

LRMS (ESI-SQ) m/z: [M-H]^-^ Calcd for C_15_H_21_N_4_O_15_P_2_ 559.05; Found 559.30.

#### Chemical Synthesis of ADP-HPM and ADP-HPD

All reactions sensitive to air and/or moisture were carried out in oven-dried (>100°C) glassware under nitrogen atmosphere and under anhydrous conditions otherwise noted. *N*,*N*,*N’*,*N’*-tetraisopropyl 9-methylfluorenylphosphoramidite was synthesized as described before (Hofer et al., 2015), acetonitrile, dichloromethane, *N*,*N*-dimethylformamide and tetrahydrofuran used in reactions were obtained from a solvent dispensing system. All other reagents were of standard commercial purity and were used as received. Analytical thin-layer chromatography was performed on EMD Merck silica gel plates with F254 indicator. Silica gel for column chromatography was purchased from Sorbent Technologies (40-75 μm particle size). Preparative C18 chromatography was performed using a Teledyne Isco CombiFlash Rf system with CombiFlash Gold columns. ^1^H, ^13^C and ^31^P NMR spectra were recorded at 500, 126, 202 MHz, respectively. Chemical shifts are reported in ppm (δ) with reference to internal residual solvent [^1^H NMR, CHCl_3_ (7.26), CHD_2_OD (3.31), HDO (4.79); ^13^C NMR, CDCl_3_ (77.0), CD_3_OD (49.0)]. Coupling constants (*J*) are reported in hertz (Hz). The following abbreviations are used to designate the multiplicities: s = singlet, d = doublet, t = triplet, q = quartet, m = multiplet, br = broad. For annotated NMR spectra of the synthesis of ADP-HPM and ADP-HPD and the synthetic intermediates see Data S1. High-resolution mass spectra were recorded by the University of Illinois Mass Spectrometry Center.

#### Synthesis Protocol of ADP-HPM

**Figure:**
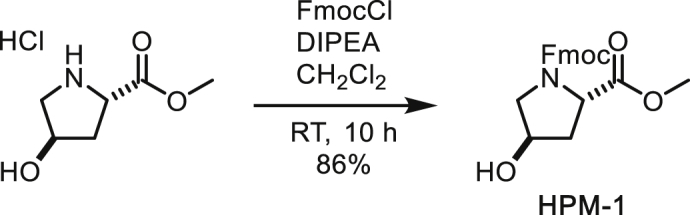

##### *N*-Fmoc-*trans*-4-hydroxyproline Methyl Ester

To a suspension of *trans*-4-hydroxyproline methyl ester (900 mg, 4.95 mmol) in CH_2_Cl_2_ (25 mL) were added DIPEA (1.41 mL, 10.9 mmol) and FmocCl (1.41 g, 5.45 mmol) at 0°C. After stirring at room temperature for 10 h, the reaction was quenched with water. The organic layer was separated and the aqueous layer was extracted with CH_2_Cl_2_. The combined organic layer was washed with brine, dried over MgSO_4_, filtered and concentrated under reduced pressure. The residue was subjected to silica gel column chromatography (55% to 75% ethyl acetate/hexane) to afford *N*-Fmoc-*trans*-4-hydroxyproline methyl ester (1.56 g, 86%) as a white solid. NMR signals were observed as 1:1 rotamers.

^**1**^**H NMR** (500 MHz, CDCl_3_): 7.75 (dd, *J* = 7.5, 2.5, 2H), 7.62-7.53 (m, 2H), 7.39 (t, *J* = 7.4 Hz, 2H), 7.30 (t, *J* = 7.4, 2H), 4.55-4.38 (m, 3H), 4.37-4.28 (m, 1H), 4.28-4.22 (m, 0.5H), 4.17-4.12 (m, 0.5H), 3.75-3.54 (m, 2H), 3.74 (s, 1.5H), 3.64 (s, 1.5H), 2.78 (br, 0.5H), 2.67 (br, 0.5H), 2.38-2.27 (m, 1H), 2.12-2.04 (m, 1H).

^**13**^**C NMR** (126 MHz, CDCl_3_) δ 173.20, 173.14, 155.14, 154.94, 144.10, 144.01, 143.85, 143.61, 141.36, 141.31, 127.78, 127.75, 127.16, 125.19, 125.10, 124.97, 120.05, 120.04, 120.01, 70.11, 69.24, 67.84, 67.69, 58.02, 57.66, 55.35, 54.72, 52.51, 52.45, 47.29, 47.21, 39.35, 38.46.

**HRMS** (ESI-TOF): *m/z* Calc. for C_21_H_21_NO_5_Na [M+Na]^+^: 390.1317, found: 390.1303.

**Figure:**
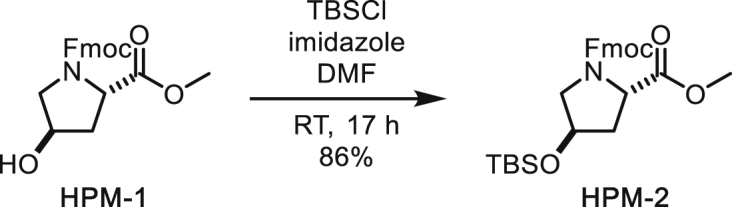

##### *N*-Fmoc-*trans*-4-*O*-TBS-proline Methyl Ester

To a solution of *N*-Fmoc-*trans*-4-hydroxyproline methyl ester (1.49 g, 4.06 mmol) in DMF (24 mL) were added imidazole (552 mg, 8.11 mmol) and TBSCl (795 mg, 5.27 mmol). After stirring at room temperature for 17 h, the reaction was diluted with diethylether and water. The organic layer was separated and the aqueous layer was extracted with diethylether. The combined organic layer was washed with brine, dried over MgSO_4_, filtered and concentrated under reduced pressure. The residue was subjected to silica gel column chromatography (10% to 25% ethyl acetate/hexane) to afford *N*-Fmoc-*trans*-4-*O*-TBS-proline methyl ester (1.68 g, 86%) as a colourless oil. NMR signals were observed as 3:2 rotamers.

^**1**^**H NMR** (500 MHz, CDCl_3_): 7.77 (dd, *J* = 7.6, 2.3 Hz, 2H), 7.64-7.53 (m, 2H), 7.40 (t, *J* = 7.4 Hz, 2H), 7.33-7.28 (m, 2H), 4.54-4.34 (m, 4H), 4.28 (t, *J* = 7.2 Hz, 0.6H), 4.19 (t, *J* = 6.8 Hz, 0.4H), 3.77 (s, 1.8H), 3.73-3.65 (m, 1H), 3.65 (s, 1.2H), 3.55-3.46 (m, 1H), 2.29-2.19 (m, 1H), 2.10-2.03 (m, 1H), 0.90 (s, 5.4H), 0.88 (s, 3.6H), 0.10-0.07 (m, 6H).

^**13**^**C NMR** (126 MHz, CDCl_3_) δ 173.32, 173.25, 155.11, 154.67, 144.27, 144.12, 144.02, 143.83, 141.42, 141.40, 127.78, 127.73, 127.19, 127.14, 125.23, 125.19, 125.14, 125.04, 120.08, 120.04, 120.02, 70.60, 69.72, 67.68, 67.66, 58.13, 57.77, 55.32, 55.02, 52.47, 52.40, 47.39, 47.26, 40.13, 39.09, 25.84, 18.10, -4.65, -4.70, -4.71, -4.74.

**HRMS** (ESI-TOF): *m/z* Calc. for C_27_H_36_NO_5_Si [M+H]^+^: 482.2363, found: 482.2350.

**Figure:**
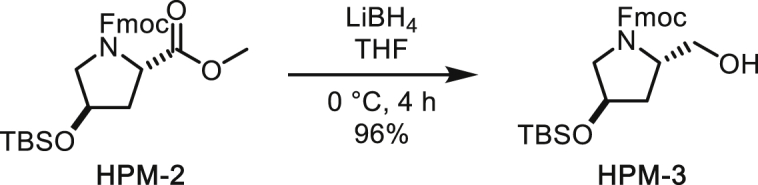

##### *N*-Fmoc-*trans*-1-hydroxymethyl-4-*O*-TBS-pyrrolidine

To a solution of *N*-Fmoc-*trans*-4-*O*-TBS-proline methyl ester (1.05 g, 2.18 mmol) in THF (11 mL) was added LiBH_4_ (237 mg, 10.9 mmol) at 0°C. The mixture was gradually warmed up to room temperature and then quenched with 1 *N* HCl after 4 h. The organic layer was separated and the aqueous layer was extracted with ethyl acetate. The combined organic layer was washed with saturated aqueous NaHCO_3_ and brine, dried over MgSO_4_, filtered and concentrated under reduced pressure. The residue was subjected to silica gel column chromatography (33% to 40% ethyl acetate/hexane) to afford *N*-Fmoc-*trans*-1-hydroxymethyl-4-*O*-TBS-pyrrolidine (953 mg, 96%) as a colourless gum.

^**1**^**H NMR** (500 MHz, CDCl_3_): 7.77 (d, *J* = 7.6 Hz, 2H), 7.60 (dd, *J* = 7.5, 4.7, 2H), 7.41 (t, *J* = 7.5 Hz, 2H), 7.32 (t, *J* = 7.4 Hz, 2H), 4.62 (m, 1H), 4.48-4.37 (m, 2H), 4.34 (m, 1H), 4.26 (t, *J* = 6.9 Hz, 1H), 4.18 (m, 1H), 3.75 (d, *J* = 11.4 Hz, 1H), 3.59 (dd, *J* = 11.6, 7.1 Hz, 1H), 3.51-3.42 (m, 2H), 2.02-1.96 (m, 1H), 1.68 (ddd, *J* = 13.1, 9.0, 4.4 Hz, 1H), 0.90 (s, 9H), 0.09 (s, 3H), 0.08 (s, 3H).

^**13**^**C NMR** (126 MHz, CDCl_3_) δ 157.25, 143.99, 143.89, 141.40, 141.38, 127.79, 127.12, 125.08, 125.04, 120.07, 120.04, 69.83, 67.67, 66.50, 59.68, 56.11, 47.29, 38.00, 25.82, 18.07, -4.66, -4.75.

**HRMS** (ESI-TOF): *m/z* Calc. for C_26_H_35_NO_4_SiNa [M+Na]^+^: 476.2233, found: 476.2218.

**Figure:**
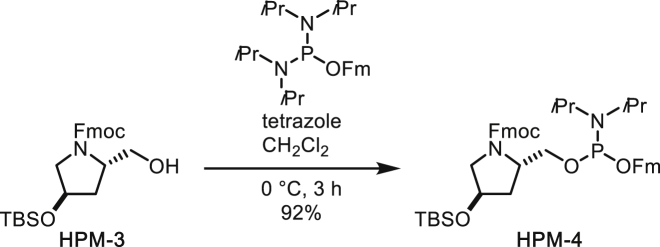

##### (*i*Pr_2_N)(*O*Fm)P-*O*-(Fmoc,TBS-HPM)

To a solution of *N*-Fmoc-*trans*-1-hydroxymethyl-4-*O*-TBS-pyrrolidine (294 mg, 0.648 mmol) and (*i*Pr_2_N)_2_P(OFm) (399 mg, 0.935 mmol) in CH_2_Cl_2_ (6.5 mL) was added tetrazole (0.45 M solution in CH_3_CN, 1.87 mL, 0.842 mmol) at 0°C. After stirring at that temperature for 3 h, the mixture was diluted with hexane. Then white precipitate was filtered and the filtrate was concentrated under reduced pressure. The residue was subjected to silica gel column chromatography (short column, eluted rapidly with 15% ethyl acetate/hexane) to afford (*i*Pr_2_N)(*O*Fm)P-*O*-(Fmoc,TBS-HPM) (464 mg, 92%) as af white solid. Intermediate hydrolysed easily, so was taken forward to next step immediately.

^**1**^**H NMR** (500 MHz, CDCl_3_): 7.83-7.60 (m, 8H), 7.47-7.29 (m, 8H), 4.62-3.48 (m, 14H), 2.26-2.16 (m, 1H), 2.09-1.99 (m, 1H), 1.27-1.17 (m, 12H), 0.96-0.91 (m, 9H), 0.14-0.09 (m, 6H).

^**13**^**C NMR** (126 MHz, CDCl_3_) δ 155.04, 144.98, 144.70, 144.29, 144.23, 144.15, 141.41, 141.36, 141.32, 141.27, 127.68, 127.65, 127.53, 127.50, 127.47, 127.43, 127.15, 127.10, 127.06, 127.02, 126.96, 126.93, 126.89, 125.51, 125.47, 125.29, 125.26, 125.24, 125.19, 125.10, 125.07, 125.03, 120.01, 119.99, 119.90, 119.84, 77.36, 70.57, 70.54, 69.96, 69.93, 67.29, 67.16, 66.41, 66.26, 66.15, 66.07, 65.95, 65.90, 64.52, 64.42, 64.31, 63.62, 63.54, 63.42, 57.35, 57.30, 57.25, 56.82, 55.52, 55.43, 55.33, 49.28, 49.22, 47.38, 43.11, 43.08, 43.02, 42.98, 38.58, 38.38, 37.67, 37.43, 25.87, 24.80, 24.74, 24.68, 24.63, 18.09, -4.61, -4.69, -4.73.

^**31**^**P NMR** (202 MHz, CDCl_3_): 148.8, 148.5, 147.6.

**HRMS** (ESI-TOF): *m/z* Calc. for C_46_H_59_N_2_O_5_PSi [M+H]^+^: 778.3931, found: 778.3952.

**Figure:**
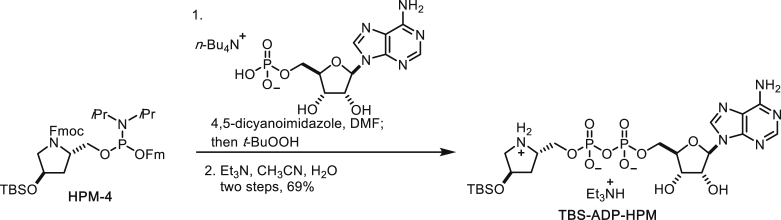

##### TBS-ADP-HPM Triethylamine Salt

To a solution of adenosinemonophosphate tetrabutylammonium salt (317 mg, 0.539 mmol) and 4,5-dicyanoimidazole (81.8 mg, 0.693 mmol) in DMF (3.85 mL) was added (*i*Pr_2_N)(*O*Fm)P-*O*-(Fmoc,TBS-HPM) (300 mg, 0.385 mmol, solution in 1.9 mL DMF). The mixture was stirred at room temperature for 30 min and then TBHP (5.5 M in decane, 0.175 mL, 0.963 mmol) was added at 0°C. The mixture was stirred at room temperature for further 30 min and solvents were evaporated. This crude pyrophosphate was dissolved in CH_3_CN (3.85 mL) and water (3.85 mL) and then Et_3_N (1.92 mL) was added for the deprotection of Fmoc and Fm groups. After stirring at room temperature for 12 h, the mixture was diluted with 50% aqueous MeOH and washed with hexane. The aqueous layer was concentrated under reduced pressure and the residue was subjected to cation exchange column (Dowex 50WX8, pre-equilibrated with Et_3_N in 20% aqueous MeOH). The solvents were evaporated and the residue was subjected to C18 column chromatography (0% to 100% CH_3_CN/water) to afford TBS-ADP-HPM triethylamine salt (198 mg, 69% for 2 steps) as a white solid.

^**1**^**H NMR** (CD_3_OD, 500 MHz): 8.55 (s, 1H), 8.20 (s, 1H), 6.09 (d, *J* = 5.4 Hz, 1H), 4.66-4.63 (m, 2H), 4.46 (dd, *J* = 5.1, 3.4 Hz, 1H), 4.35-4.23 (m, 4H), 4.14-4.05 (m, 2H), 3.38 (dd, *J* = 12.1, 3.4 Hz, 1H), 3.15 (q, *J* = 7.3 Hz, 6H), 2.05 (dd, *J* = 8.3, 2.8 Hz, 2H), 1.30 (t, *J* = 7.3 Hz, 9H), 0.92 (s, 9H), 0.11 (s, 6H).

^**13**^**C NMR** (CD_3_OD, 125 MHz): 157.2, 153.8, 150.7, 141.0, 120.2, 89.2, 85.2 (d, ^3^*J*_CP_ = 9.1 Hz), 76.2, 72.9, 71.8, 66.5 (d, ^2^*J*_CP_ = 5.1 Hz), 65.3 (d, ^2^*J*_CP_ = 4.2 Hz), 59.6 (d, ^3^*J*_CP_ = 8.4 Hz), 54.6, 47.4, 37.0, 26.2, 18.8, 9.1, -4.8, -4.9.

^**31**^**P NMR** (CD_3_OD, 202 MHz): -10.22 (d, *J* = 22.4 Hz), -10.58 (d, *J* = 22.4 Hz).

**HRMS** (ESI-TOF) m/z: [M+H]^+^ Calc. for C_21_H_39_N_6_O_11_P_2_Si 641.1921; Found 641.1917.

**Figure:**
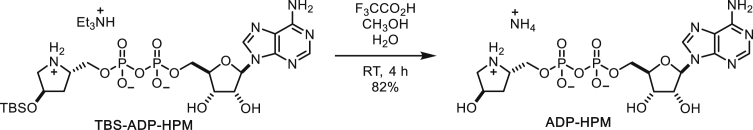

**ADP-HPM**: TBS-ADP-HPM ammonium salt was prepared by the previous procedure with NH_4_OH for pre-equilibration in cation exchange step. To a solution of monoTBS-ADP-HPM (33.8 mg, 0.0514 mmol) in MeOH (1 mL) and water (1 mL) was added TFA (0.5 mL). The mixture was stirred at room temperature for 4.5 h and the solvents were evaporated. The residue was subjected to C18 column chromatography (0% to 100% CH_3_CN/40 mM NH_4_OAc aq) to afford ADP-HPM (22.8 mg, 82%) as a white solid.

^**1**^**H NMR** (500 MHz, Deuterium Oxide) δ 8.38 (s, 1H), 8.09 (s, 1H), 6.03 (d, *J* = 5.6 Hz, 1H), 4.67 (t, *J* = 5.4 Hz, 1H), 4.56 (t, *J* = 4.0 Hz, 1H), 4.45 (dd, *J* = 5.1, 3.8 Hz, 1H), 4.32 (br, 1H), 4.22 (dt, *J* = 11.7, 3.7 Hz, 1H), 4.16 (s, 1H), 4.09 (dtd, *J* = 9.7, 6.6, 2.9 Hz, 1H), 3.97 (dt, *J* = 11.5, 5.8 Hz, 1H), 3.33 (dd, *J* = 12.6, 3.6 Hz, 1H), 3.24 (dt, *J* = 12.6, 1.5 Hz, 1H), 2.06 (ddt, *J* = 14.2, 7.2, 1.7 Hz, 1H), 1.96 (ddd, *J* = 14.3, 10.4, 4.3 Hz, 1H).

^**13**^**C NMR** (126 MHz, Deuterium Oxide) δ 155.34, 152.66, 148.82, 139.57, 118.38, 86.88, 83.61 (d, ^3^*J*_CP_ = 8.6 Hz), 74.17, 70.22, 69.70, 65.25 (d, ^2^*J*_CP_ = 4.7 Hz), 64.14 (d, ^2^*J*_CP_ = 4.6 Hz), 58.07 (d, ^3^*J*_CP_ = 8.0 Hz), 52.65, 34.29.

^**31**^**P NMR** (202 MHz, Deuterium Oxide) δ -11.20, -10.45.

**HRMS** (ESI-TOF) m/z: [M+H]^+^ Calc. for C_15_H_25_N_6_O_11_P_2_ 527.1057; Found 527.1046.

#### Synthesis Protocol of ADP-HPD

**Figure:**
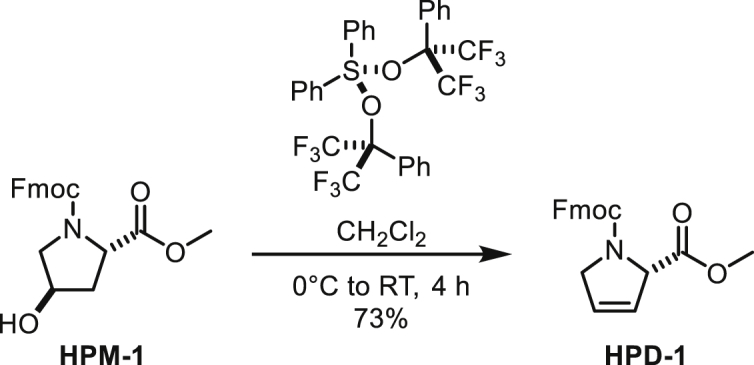

##### *N*-Fmoc-L-3,4-dehydroproline Methyl Ester (HPD-1)

To a stirring solution of **HPM-1** (2.52 g, 6.85 mmol) in CH_2_Cl_2_ (60 mL) at 0°C was added Martin’s Sulfurane dehydrating agent (4.93 g, 7.33 mmol, 1.1 eq). Reaction mixture was stirred at 0°C for 1 h then allowed to warm to room temperature. After stirring for 3 h, the reaction mixture was quenched by the addition of satd aq NH_4_Cl. Aqueous layer was extracted with CH_2_Cl_2_. Combined organic layers were washed with brine, dried over anhydrous MgSO_4_, filtered and evaporated. Residue was purified by silica gel chromatography (26% EtOAc/hexane) to provide expected alkene with some diphenyl sulfoxide contaminant. Diphenyl sulfoxide was removed by further purification via C18 chromatography, product eluting at ∼60% H_2_O/THF (1.75 g, 73%). Observed rotamers in 52:48 ratio.

^**1**^**H NMR** (500 MHz, CDCl_3_) δ 7.79 – 7.74 (m, 2H), 7.68 – 7.63 (m, 5H), 7.63 – 7.52 (m, 2H), 7.48 – 7.42 (m, 7H), 7.40 (td, *J* = 7.4, 2.9 Hz, 2H), 7.32 (dddt, *J* = 7.4, 5.3, 2.5, 1.3 Hz, 2H), 6.03 – 5.96 (m, 1H), 5.83 – 5.77 (m, 0.5H), 5.77 – 5.72 (m, 0.5H), 5.15 – 5.10 (m, 0.5H), 5.01 (ddd, *J* = 4.7, 2.4, 1.2 Hz, 0.5H), 4.49 (ddd, *J* = 10.5, 6.6, 2.0 Hz, 1H), 4.44 – 4.35 (m, 1.5H), 4.35 – 4.26 (m, 2H), 4.19 (t, *J* = 6.7 Hz, 0.5H), 3.76 (d, *J* = 0.9 Hz, 1.5H), 3.65 (d, *J* = 1.0 Hz, 1.5H).

^**13**^**C NMR** (126 MHz, CDCl_3_) δ 170.53, 170.43, 154.43, 154.17, 145.72, 144.16, 144.13, 143.92, 143.80, 141.42, 141.39, 131.13, 129.41, 129.23, 129.14, 127.81, 127.79, 127.74, 127.16, 127.14, 127.12, 125.23, 125.14, 125.04, 124.97, 124.92, 124.87, 124.83, 124.80, 120.08, 120.06, 120.04, 67.68, 67.62, 66.68, 66.23, 54.06, 53.50, 52.56, 52.53, 47.38, 47.30.

**HRMS** (ESI): *m/z* calc. for C_21_H_20_NO_4_ [M+H]^+^: 350.1392, found: 350.1386.

**Figure:**
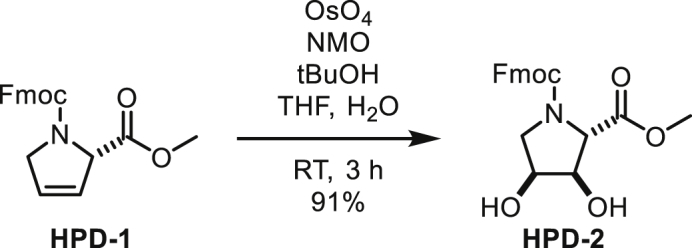

##### *N*-Fmoc-(2*S*,3*R*,4*S*)-3,4-dihydroxyproline Methyl Ester (HPD-2)

To a stirring solution of **HPD-1** (1.59 g, 4.56 mmol) in t-BuOH (20 mL), THF (5 mL) and H_2_O (1 mL) was added OsO_4_ as a 0.2 M solution in CH_3_CN (1.0 mL, 0.2 mmol, 4 mol %) and *N*-methylmorpholine-*N*-oxide (810 mg, 6.9 mmol, 1.5 eq). Reaction mixture was stirred at RT for 3 h then quenched with satd aq Na_2_SO_3_ and diluted with EtOAc (150 mL). Biphasic mixture was stirred for 1 h then separated. Organic layer was washed with satd aq NH4Cl and brine, dried over MgSO_4_, filtered and evaporated. Residue was purified by silica gel chromatography (50% to 75% EtOAc/hexane) to provide expected product as a 9:1 mixture of diastereomers (1.597 g, 91%).

^**1**^**H NMR** (500 MHz, CDCl_3_) δ 7.75 (dd, *J* = 7.6, 5.0 Hz, 2H), 7.61 – 7.50 (m, 2H), 7.39 (td, *J* = 7.5, 3.5 Hz, 2H), 7.34 – 7.27 (m, 2H), 4.47 – 4.21 (m, 6H), 4.13 (td, *J* = 7.0, 4.0 Hz, 1H), 3.83 – 3.78 (m, 1H), 3.76 (s, 1H), 3.73 (dd, *J* = 11.6, 5.3 Hz, 1H), 3.66 (s, 1H), 3.59 (ddd, *J* = 24.7, 11.4, 4.3 Hz, 1H).

^**13**^**C NMR** (126 MHz, CDCl_3_) δ 171.62, 155.22, 154.95, 143.96, 143.86, 143.79, 143.59, 141.37, 127.88, 127.84, 127.22, 125.19, 125.17, 125.07, 124.96, 120.13, 120.09, 75.97, 74.89, 70.68, 69.83, 68.01, 64.81, 64.58, 52.90, 52.80, 51.29, 51.02, 47.24, 47.16.

**HRMS** (ESI): *m/z* calc. for C_21_H_22_NO_6_ [M+H]^+^: 384.1447, found: 384.1444.

**Figure:**
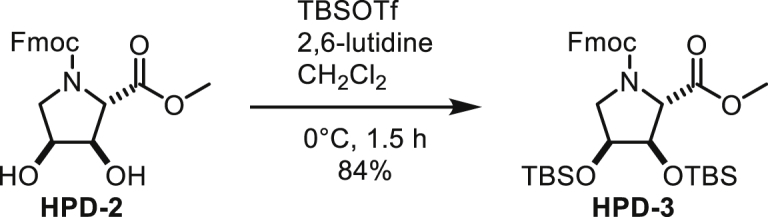

##### *N*-Fmoc-(2*R*,3*R*,4*S*)-3,4-bis((*tert*-butyldimethylsilyl)oxy)-proline Methyl Ester (HPD-3)

To a stirring solution of **HPD-2** (1.52 g, 3.96 mmol) in CH_2_Cl_2_ at 0°C added 2,6-lutidine (1.40 mL, 1.30 g, 12.1 mmol, 3 eq) and TBSOTf (2.74 g, 10.4 mmol, 2.6 eq). Reaction mixture was stirred at 0°C for 1.5 h and quenched with H_2_O. Mixture was extracted with CH_2_Cl_2_. Combined organic layers were washed with brine, dried over MgSO_4_, filtered and evaporated. Residue was purified by silica gel chromatography (10% EtOAc/hexane) to provide some fractions of a single diastereomer (1.569 g, 65%) and others as a mixture of diastereomers (459 mg, 19%). Two rotamers were observed by NMR (55:45).

^**1**^**H NMR** (500 MHz, CDCl_3_) δ 7.76 (t, *J* = 6.9 Hz, 2H), 7.63 – 7.51 (m, 2H), 7.39 (q, *J* = 7.2 Hz, 2H), 7.30 (td, *J* = 7.5, 2.9 Hz, 2H), 4.44 – 4.33 (m, 2H), 4.29 (t, *J* = 7.2 Hz, 0.55H), 4.24 (d, *J* = 3.0 Hz, 0.55H), 4.22 – 4.12 (m, 3H), 3.78 (d, *J* = 1.2 Hz, 1.6H), 3.74 – 3.68 (m, 1H), 3.67 (d, *J* = 1.2 Hz, 1.4H), 3.45 (dd, *J* = 10.2, 6.3 Hz, 0.44H), 3.40 (dd, *J* = 9.8, 6.4 Hz, 0.55H), 0.92 (s, 14H), 0.89 (s, 4H), 0.10 (s, 9H), 0.08 (s, 3H).

^**13**^**C NMR** (126 MHz, CDCl_3_) δ 171.53, 171.52, 155.10, 154.73, 144.28, 144.13, 144.04, 143.85, 141.48, 141.45, 141.43, 141.41, 127.84, 127.82, 127.75, 127.22, 127.17, 127.14, 125.26, 125.23, 125.16, 125.03, 120.11, 120.06, 77.02, 76.12, 71.82, 71.21, 67.74, 67.67, 65.65, 65.64, 52.57, 52.52, 50.51, 50.49, 47.35, 47.29, 25.98, 25.96, 25.87, 18.28, -4.40, -4.42, -4.45, -4.52, -4.55, -4.56, -4.98, -5.03.

**HRMS** (ESI): *m/z* calc. for C_33_H_50_NO_6_Si_2_ [M+H]^+^: 612.3177, found: 612.3177.

**Figure:**
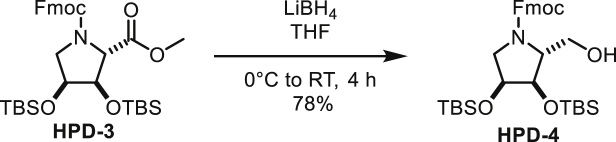

##### *N*-Fmoc-(2*R*,3*R*,4*S*)-3,4-bis((*tert*-butyldimethylsilyl)oxy)-2-(hydroxymethyl)pyrrolidine (HPD-4)

To a stirring solution of **HPD-3** (1485 mg, 2.43 mmol) in THF (24 mL) at 0°C was added lithium borohydride (305 mg, 14 mmol, 6 eq). Reaction mixture was stirred at 0°C for 2 h and then slowly warmed to RT and stirred for an additional 2 h. Reaction was cooled to 0°C and quenched by addition of 1 M HCl. Mixture was diluted with satd aq NH_4_Cl and extracted with EtOAc. Combined organic layers were washed with NaHCO3 and brine, dried over Na_2_SO_4_ and evaporated. Residue was purified by silica gel chromatography (25% EtOAc/hexane) to provide white solid (1.10 g, 78%).

^**1**^**H NMR** (500 MHz, CDCl_3_) δ 7.77 (d, *J* = 7.5 Hz, 2H), 7.62 – 7.53 (m, 2H), 7.41 (t, *J* = 7.5 Hz, 2H), 7.31 (t, *J* = 7.5 Hz, 2H), 4.67 – 4.54 (m, 0.5H), 4.44 (d, *J* = 6.9 Hz, 1.5H), 4.26 (t, *J* = 6.9 Hz, 1H), 4.07 (td, *J* = 5.5, 3.6 Hz, 1H), 3.99 (s, 0.25H), 3.89 – 3.77 (m, 2.3H), 3.58 (dd, *J* = 11.1, 6.4 Hz, 0.77H), 3.43 – 3.30 (m, 2H), 3.21 (t, *J* = 4.3 Hz, 0.74H), 0.95 – 0.88 (m, 9H), 0.86 (s, 3H), 0.13 – 0.01 (m, 9H).

^**13**^**C NMR** (126 MHz, CDCl_3_) δ 157.02, 144.07, 143.94, 141.55, 141.48, 127.88, 127.86, 127.21, 127.16, 125.17, 125.09, 120.16, 120.10, 74.59, 71.22, 67.56, 66.05, 64.10, 51.48, 47.43, 25.98, 25.96, 18.27, -4.10, -4.40, -4.47, -4.79.

**HRMS** (ESI): *m/z* calc. for C_32_H_50_NO_5_Si_2_ [M+H]^+^: 584.3228, found: 584.3226.

**Figure:**
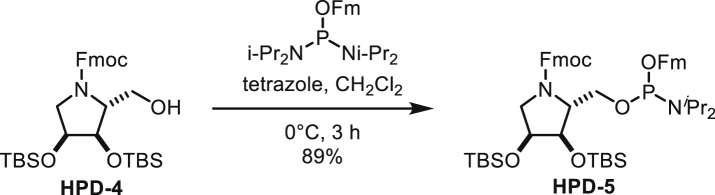

*N*-Fmoc-(2*R*,3*R*,4*S*)-3,4-bis((*tert*-butyldimethylsilyl)oxy)-2-(hydroxymethyl)pyrrolidine 9-fluorenylmethyl diisoproylphosphoramidite (HPD-5): To a stirring solution of HPD-4 (40.3 mg, 0.069 mmol) and 9-fluorenylmethyl tetraisopropylphosphorodiamidite (40.1 mg, 0.094 mmol, 1.4 eq) in CH_2_Cl_2_ (2 mL) at 0°C was added tetrazole as a 0.45 M solution in CH_3_CN (0.18 mL, 0.081 mmol, 1.2 eq). Reaction mixture was stirred at 0°C for 3 h. Solvent was removed by rotary evaporation and residue was purified by silica gel chromatography (89:10:1 hexane:EtOAc:diethylmethylamine) to provide phosphoramidite as a white foam (344.4 mg, 89%). Despite appearing as two species by ^31^P NMR (likely rotamers), spectra of other nuclei reveal a complex mixture of rotamers. Compound decomposed analysed by mass spectrometry.

^**1**^**H NMR** (600 MHz, CDCl_3_) δ 7.79 – 7.72 (m, 4H), 7.72 – 7.53 (m, 5H), 7.45 – 7.22 (m, 8H), 4.57 – 4.49 (m, 0H), 4.40 (ddd, *J* = 27.1, 10.4, 7.3 Hz, 1H), 4.36 – 4.12 (m, 4H), 4.00 (ddt, *J* = 18.7, 8.6, 6.5 Hz, 1H), 3.87 – 3.74 (m, 2H), 3.69 (ddt, *J* = 14.5, 5.5, 3.7 Hz, 1H), 3.61 (ttt, *J* = 14.2, 6.9, 3.7 Hz, 2H), 3.54 – 3.47 (m, 1H), 3.44 – 3.33 (m, 1H), 1.20 (d, *J* = 6.8 Hz, 2H), 1.18 – 1.10 (m, 11H), 0.95 – 0.87 (m, 18H), 0.12 – 0.05 (m, 12H).

^**13**^**C NMR** (151 MHz, CDCl_3_) δ 155.62, 155.58, 155.43, 155.42, 145.22, 145.14, 145.07, 144.86, 144.80, 144.78, 144.45, 144.38, 144.36, 144.31, 144.26, 141.64, 141.61, 141.56, 141.54, 141.48, 129.02, 128.32, 127.91, 127.74, 127.71, 127.34, 127.26, 127.14, 127.08, 125.79, 125.71, 125.46, 125.33, 125.28, 125.25, 125.21, 120.21, 120.10, 120.03, 75.18, 74.92, 74.65, 74.44, 71.48, 71.46, 71.03, 71.01, 67.58, 67.49, 67.32, 66.65, 66.52, 66.48, 66.44, 66.37, 66.32, 66.28, 65.99, 65.94, 65.66, 65.60, 65.53, 65.43, 65.37, 62.27, 62.17, 61.98, 61.88, 61.57, 61.46, 61.30, 61.21, 50.96, 50.85, 50.80, 50.69, 49.52, 49.47, 47.60, 47.55, 43.34, 43.31, 43.27, 43.24, 26.17, 25.03, 24.87, 24.83, 18.50, -4.08, -4.22, -4.34, -4.40, -4.51, -4.60.

^**31**^**P NMR** (243 MHz, CDCl_3_) δ 147.30, 147.13.

**Figure:**
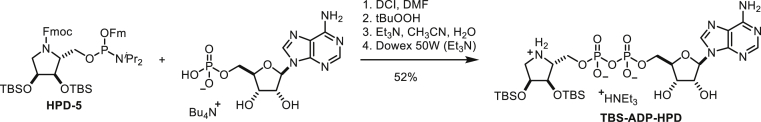

##### Triethylammonium 3,4-bis((*tert*-butyldimethylsilyl)oxy)-ADP-HPD

To a 10 mL flask added tetrabutylammonium AMP (292 mg, 0.497 mmol, 1.4 eq) and 4,5-dicyanoimidazole (76.1 mg, 0.644 mmol, 1.8 eq). Dissolved in 4 mL DMF. **HPD-5** (320.8 mg, 0.353 mmol, 1 eq) was added as a solution in 4 mL DMF. Reaction mixture was stirred at RT for 1 h, then cooled to 0°C and t-BuOOH was added as a 5.5 M solution in decane (0.13 mL, 0.72 mmol, 2.0 eq). Reaction was stirred at 0°C for 1 h. Solvent was removed by rotary evaporation (60°C bath temp.). Residue was redissolved in CH_3_CN (5 mL) and H_2_O (5 mL). Triethylamine (3 mL) was added and mixture was stirred overnight at RT. Solvent was removed by rotary evaporation. Tetrabutylammonium cation was exchanged by elution through Dowex 50Wx2 resin (Et_3_N-form). Purification via C18 chromatography was performed, product eluting with ∼30% CH_3_CN/H_2_O (160.2 mg, 52%).

^**1**^**H NMR** (500 MHz, CD_3_OD) δ 8.55 (s, 1H), 8.20 (s, 1H), 6.09 (d, *J* = 5.4 Hz, 1H), 4.64 (t, *J* = 5.3 Hz, 1H), 4.45 (dd, *J* = 5.1, 3.5 Hz, 1H), 4.39 – 4.32 (m, 3H), 4.29 – 4.23 (m, 3H), 4.12 (dt, *J* = 11.9, 4.9 Hz, 1H), 3.64 – 3.58 (m, 1H), 3.44 (dd, *J* = 12.2, 3.0 Hz, 1H), 3.19 (q, *J* = 7.3 Hz, 6H), 3.14 (dd, *J* = 12.2, 1.8 Hz, 1H), 1.30 (t, *J* = 7.3 Hz, 9H), 0.94 (s, 9H), 0.93 (s, 9H), 0.18 (s, 3H), 0.15 (s, 3H), 0.14 (s, 6H).

^**13**^**C NMR** (126 MHz, CD_3_OD) δ 157.22, 153.77, 150.86, 141.11, 120.20, 89.12, 85.39 (d, *J* = 8.9 Hz), 76.20, 73.88, 73.27, 71.84, 66.59 (d, *J* = 5.71 Hz), 62.79 (d, *J* = 4.7 Hz), 62.18 (d, *J* = 8.4 Hz), 51.07, 47.76, 26.39, 26.35, 18.93, 18.82, 9.19, -4.00, -4.41, -4.48, -4.81.

^**31**^**P NMR** (203 MHz, CD_3_OD) δ -11.25 (d, *J* = 22.5 Hz), -11.83 (d, *J* = 22.5 Hz).

**HRMS** (ESI): *m/z* calc. for C_27_H_53_N_6_O_12_Si_2_P_2_ [M+H]^+^: 771.2735, found 771.2714.

**Figure:**
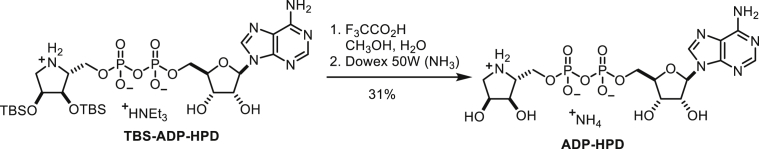

##### ADP-HPD

To a stirring solution of **TBS-ADP-HPD** (142 mg, 0.163 mmol) in 1:1 CH_3_OH:H_2_O (6 mL) was added trifluoroacetic acid (0.75 mL). Reaction mixture was stirred at RT for 2 h. Solvent was removed by rotary evaporation. Residue was purified by ion-pairing chromatography (40 mM Et_3_N⋅HOAc, pH 6.5). Product-containing fractions were combined and evaporated. Triethylammonium cation was exchanged for ammonium cation by elution through Dowex 50Wx2 resin (NH_4_-form). After lyophilizing twice from H_2_O, obtained ADP-HPD as adduct with one equivalent of ammonium acetate (28 mg, 31%).

^**1**^**H NMR** (600 MHz, D_2_O) δ 8.43 (s, 1H, adenosyl H-8), 8.15 (s, 1H, adenosyl H-2), 6.09 (d, *J* = 5.6 Hz, 1H, adenosyl H-1’), 4.73 (t, *J* = 5.4 Hz, 1H, adenosyl H-2’), 4.52 (dd, *J* = 5.1, 4.0 Hz, 1H, adenosyl H-3’), 4.42 – 4.34 (m, 4H), 4.26 – 4.18 (m, 3H), 3.79 – 3.75 (m, 1H), 3.49 (dd, *J* = 12.9, 3.8 Hz, 1H, pyrrolidine H-5), 3.37 (dd, *J* = 12.9, 1.9 Hz, 1H, pyrrolidine H-5’), 1.92 (s, 3H, acetate).

^**13**^**C NMR** (126 MHz, D_2_O) δ 181.34 (acetate C-1), 155.40 (adenosyl C-6), 152.74 (adenosyl C-2), 148.90 (adenosyl C-4), 139.71 (adenosyl C-8), 118.49 (adenosyl C-5), 87.08 (adenosyl C-1’), 83.66 (d, *J*^CP^ = 8.7 Hz, adenosyl C-4’), 74.30 (adenosyl C-2’), 71.03, 70.32 (adenosyl C-3’), 69.60, 65.40 (d, *J*^CP^ = 5.5 Hz, pyrrolidine C-6), 62.26 (d, *J* = 4.9 Hz, adenosyl C-5’), 60.51 (d, *J* = 8.5 Hz, pyrrolidine C-2), 49.76 (pyrrolidine C-5), 23.22 (acetate C-2).

^**31**^**P NMR** (243 MHz, D_2_O) δ -10.98 (d, *J* = 21.0 Hz), -11.35 (d, *J* = 21.0 Hz).

**HRMS** (ESI): *m/z* calc. for C_15_H_25_N_6_O_12_P_2_ [M+H]^+^: 543.1006, found 543.1000.

**Figure:**
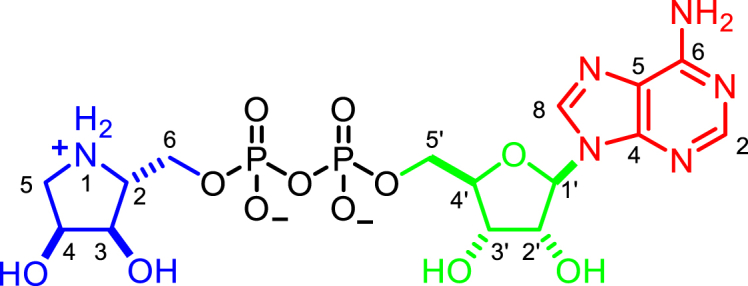

**Figure:**
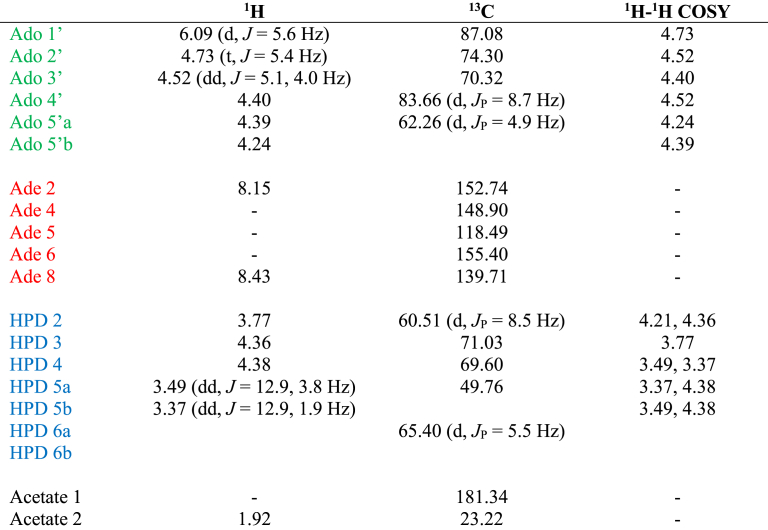

### Data and Software Availability

The atomic coordinates and structure factors for the *h*ARH1:ADPr, *h*ARH1:ADP-HPM, apo-*Lch*ARH3, *Lch*ARH3:ADPr, *Lch*ARH3:ADP-HPD, *Lch*ARH3:ADP-HPM, *Lch*ARH3:IDPr, *Lch*ARH3:Arg-ADPr and PARG:ADP-HPM structures reported in this paper have been deposited in the RCSB Protein Data Bank (www.rcsb.org) under accession codes 6G28, 6G2A, 6G1P, 6G1Q, 6HGZ, 6HH3, 6HH5, 6HOZ, 6HH4, 6HH6, respectively.

## Supplemental Information

Document S1. Figures S1–S7 and Tables S1–S3

Data S1. NMR Spectra Concerning the Synthesis of ADP-HPM, Related to STAR Methods

Document S2. Article plus Supplemental Information
