# Supplementary material for: (ADP-ribosyl)hydrolases: Structural Basis for Differential Substrate Recognition and Inhibition
Source: Cell Chem Biol. 2018 Dec 20;25(12):1533–1546.e12. doi: 10.1016/j.chembiol.2018.11.001 (PMC6309922; doi:10.1016/j.chembiol.2018.11.001)
Supplement: Document S1. Figures S1–S7 and Tables S1–S3 [file mmc1.pdf]

Cell Chemical Biology, Volume *25*

Supplemental Information

(ADP-ribosyl)hydrolases: Structural Basis for Differential Substrate Recognition and Inhibition

Johannes Gregor Matthias Rack, Antonio Ariza, Bryon S. Drown, Callum Henfrey, Edward Bartlett, Tomohiro Shirai, Paul J. Hergenrother, and Ivan Ahel

Supplemental Information Content

Tables

Table S1. Comparison of structure similarity. **III**

Table S2. Validation of metal-binding with CheckMyMetal. **IV**

Table S3. Sequences used to generate the alignment of ARH1 and ARH3. **V**

Figures

Figure S1. Ligand coordination diagrams. **VI**

Figure S2. Quality control experiments. **VII**

Figure S3. Multiple sequence alignment of ARH1 and ARH3. **VIII**

Figure S4. Evolutionary conservation of metal preference and inhibitor sensitivity. **IX**

Figure S5. Comparison of the coordination of ADPr, ADP-HPD and ADP-HPM by PARG and *Lch*ARH3. **X**

Figure S6. Potential substrate binding surfaces. **XI**

Figure S7. Chemical synthesis schemes. **XII**

Table S1. Comparison of structure similarity.[a] (Related to Results and Table 1)

structure[b]	compared to	RMSD (Å)	over (n) C^{α}
*h*ARH1:ADPr	*h*ARH1:ADP[c]	0.192	290
hARH1:ADP-HPM	*h*ARH1:ADP[c]	0.190	291
	*h*ARH1:ADPr[c]	0.211	285
*Lch*ARH3 apo	*h*ARH3 apo[d]	0.401	243
	*m*ARH3 apo[e]	0.380	234
*Lch*ARH3:ADPr (initial)	*h*ARH3 apo[d]	0.463	263
	*m*ARH3 apo[e]	0.427	266
	*Lch*ARH3 apo[b]	0.217	258
*Lch*ARH3:ADPr (optimised)	*h*ARH3 apo[d]	0.437	259
	*m*ARH3 apo[e]	0.429	272
	*Lch*ARH3 apo[c]	0.189	263
*Lch*ARH3:ADP-HPD	*h*ARH3 apo[d]	0.405	247
	*m*ARH3 apo[e]	0.403	261
	*Lch*ARH3:ADPr (optimised)[b]	0.099	267
*Lch*ARH3:ADP-HPM	*h*ARH3 apo[d]	0.459	257
	*m*ARH3 apo[e]	0.462	271
	*Lch*ARH3:ADPr (optimised)[b]	0.192	286
*Lch*ARH3:IDPr	*h*ARH3 apo[d]	0.398	249
	*m*ARH3 apo[e]	0.413	259
	*Lch*ARH3:ADPr (optimised)[b]	0.166	289
*Lch*ARH3:Arg-ADPr	*h*ARH3 apo[d]	0.397	241
	*m*ARH3 apo[e]	0.400	249
	*Lch*ARH3:ADPr (optimised)[b]	0.177	271
PARG:ADP-HPM	PARG:ADPr[f]	0.172	458
	PARG:ADP-HPD[g]	0.171	445

(a) Similarity was calculated using PyMOL (Molecular Graphics System, Version 1.8 Schrödinger, LLC)
(b) This study
(c) PDB 3HFW
(d) PDB 2FOZ
(e) PDB 2QTY
(f) PDB 4B1H
(g) PDB 4B1J

Table S2. Validation of metal-binding with CheckMyMetal.[a] (Related to Figure 2 and Table 1)

Metal	ID	Occupancy	B factor (env.)[b]	Ligands	Geometry	gRMSD	Vacancy	Bidentate
*h*ARH1:ADP complex (PDB 3HFW)[c]								
Mg	Mg_I	1	17.4 (11.3)	O_6	Octahedral	6.2°	0	0
K		1	16.3 (9.4)	O_6	Octahedral	*17.6°*	0	0
*h*ARH1:ADPr complex (PDB 6G28)[d]								
Mg	Mg_I	1	8.5 (8.9)	O_6	Octahedral	8.1°	0	0
Mg	Mg_{II}	1	9.4 (9.8)	O_6	Octahedral	6.1°	0	0
*h*ARH1:ADP-HPM complex (PDB 6G2A)[d]								
Mg	Mg_I	1	14.2 (14.0)	O_6	Octahedral	9.7°	0	0
Mg	Mg_{free}	1	25.1 (24.6)	O_6	Octahedral	2.9°	0	0
apo *h*ARH3 (PDB 2FOZ)[e]								
Mg	Mg_I	1	*11.4 (19.3)*	O_6	Octahedral	4.9°	0	0
Mg	Mg_{II}	1	*11.3 (19.3)*	O_6	Octahedral	6.4°	0	0
apo *m*ARH3 (PDB 2QTY)[f]								
Mg	Mg_I	1	*8.2 (15.1)*	O_6	Octahedral	5.3°	0	0
Mg	Mg_{II}	1	*12.9 (16.7)*	O_6	Octahedral	7.9°	0	0
*h*ARH3:ADPr complex (PDB 5ZQY)[g]								
Mg	Mg_I	1	**12.7 (24.5)**	O_7	**Pentagonal Bipyramidal**	10°	0	0
Mg	Mg_{II}	1	*17.4 (25.9)*	O_6	Octahedral	*18.3°*	0	0
*h*ARH3:ADPr complex (PDB 6D36)[h]								
Mg	Mg_I	1	27.1 (24.9)	O_6	Octahedral	11.2°	0	0
Mg	Mg_{II}	1	*19.1 (25.2)*	O_6	Octahedral	*14.6°*	0	0
apo *Lch*ARH3 (PDB 6G1P)[d]								
Mg	Mg_I	1	*16.8 (19.4)*	O_6	Octahedral	5.4°	0	0
*Lch*ARH3:ADPr complex (PDB 6G1Q)[d]								
Mg	Mg_I	1	53.1 (48.9)	O_6	Octahedral	13.2°	0	0
*Lch*ARH3:ADPr complex (PDB 6HGZ)[d]								
Mg	Mg_I	1	25.5 (24.3)	O_6	Octahedral	10.4°	0	0
Mg	Mg_{II}	1	30.1 (27.9)	O_6	Octahedral	*20.1°*	0	1
*Lch*ARH3:ADP-HPD complex (PDB 6HH3)[d]								
Mg	Mg_I	1	41.1 (41.3)	O_6	Octahedral	*13.8°*	0	0
Mg	Mg_{II}	1	49.5 (46.9))	O_6	Octahedral	12.1°	0	0
*Lch*ARH3:ADP-HPM complex (PDB 6HH5)[d]								
Mg	Mg_I	1	62.2 (55.3)	O_6	Octahedral	*16.6°*	0	0
*Lch*ARH3:IDPr complex (PDB 6HOZ)[d]								
Mg	Mg_I	1	28.6 (28.7)	O_6	Octahedral	*17°*	0	0
Mg	Mg_{II}	1	34.4 (31.9)	O_6	Octahedral	8.9°	0	0
*Lch*ARH3:Arg-ADPr complex (PDB 6HH4)[d]								
Mg	Mg_I	1	29.3 (29.2)	O_6	Octahedral	*16°*	0	0
Mg	Mg_{II}	1	33.4 (29.9)	O_6	Octahedral	7.1°	0	0

(a) Underlined: Borderline values are given in *italic* and outliers in **bold**.
(b) Metal B factor, with valence-weighted environmental average B factor in parenthesis
(c) Unpublished data
(d) This study
(e) (Mueller-Dieckmann et al., 2006)
(f) (Mueller-Dieckmann et al., 2008)
(g) (Wang et al., 2018)
(h) (Pourfarjam et al., 2018)

Table S3. Sequences used to generate the alignment of ARH1 and ARH3. (Related to Figure 3)

common name	binominal name	accession[a]
ARH1		
human	*Homo sapiens*	NP_001116.1
sheep	*Ovis aries*	XP_011950441.1
mouse	*Mus musculus*	NP_031440.1
Tasmanian devil	*Sarcophilus harrisii*	XP_003766517.1
great tit	*Parus major*	XP_015495276.1
wild turkey	*Meleagris gallopavo*	XP_003202881.2
African clawed frog	*Xenopus laevis*	NP_001087730.1
common garter snake	*Thamnophis sirtalis*	XP_013932088.1
gombessa	*Latimeria chalumnae*	XP_006010000.1
Atlantic salmon	*Salmo salar*	NP_001133133.1
zebrafish	*Danio rerio*	NP_001003453.1
ARH3		
human	*Homo sapiens*	NP_060295.1
European mouflon	*Ovis aries musimon*	XP_011971959.1
mouse	*Mus musculus*	NP_598644.1
Tasmanian devil	*Sarcophilus harrisii*	XP_003765409.2
great tit	*Parus major*	XP_015504659.1
helmeted guineafowl	*Numida meleagris*	XP_021231189.1
tropical clawed frog	*Xenopus tropicalis*	CAJ81573.1
common garter snake	*Thamnophis sirtalis*	XP_013916955.1
gombessa	*Latimeria chalumnae*	XP_005988572.1
Atlantic salmon	*Salmo salar*	NP_001135008.1
zebrafish	*Danio rerio*	NP_001004565.2

(a) GenBank accession numbers

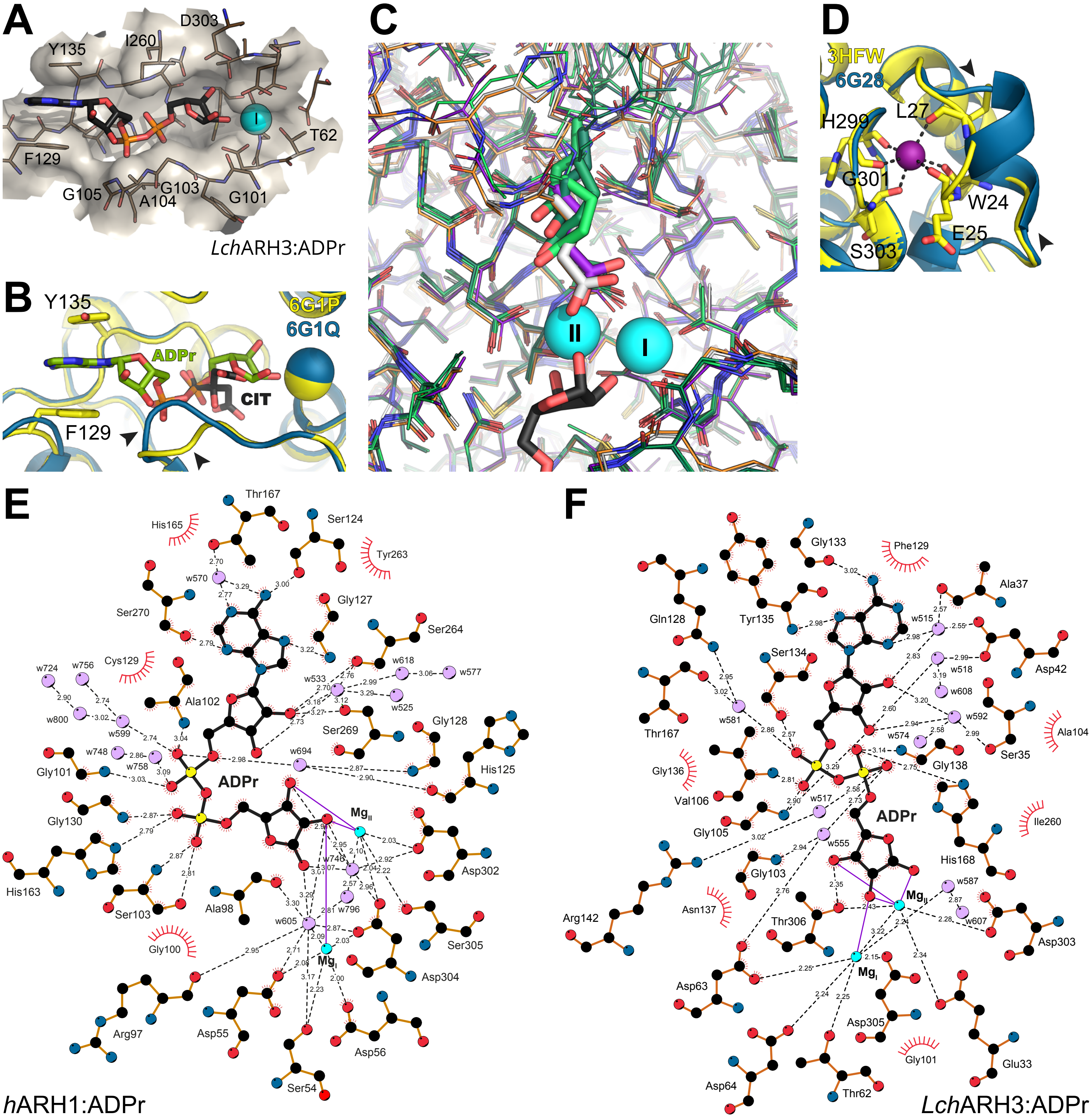

Figure S1. Ligand coordination diagrams. (Related to Figure 2 and 4)
(A) Liquorice-surface representation of the *Lch*ARH3 (brown) in complex with ADPr (black) structure obtained with the initial crystal system prior to optimisation. Residues important for the interaction are highlighted. Note, the absence of Mg_{II} form the structure. **(B)** Comparison between apo-*Lch*ARH3 (blue) and *Lch*ARH3:ADPr (yellow:green) shows movement of parts of loop 4 (indicated by arrows). The closed apo form is stabilized by a co-crystalized citrate molecule (black). Mg^{2+} ions (in colour of corresponding backbone) and the adenosine coordinating residues Phe129 and Tyr135 are given for orientation. **(C)** Liquorice representation of Glu33 (*Lch*ARH3) flexibility as highlighted by the comparison of different structures: *m*ARH3 apo (white; PDB 2QTY), *h*ARH3 apo (orange; PDB 2FOZ), *h*ARH3:ADPr (green gradient according to protomer light A to dark D; PDB 6D36) and *Lch*ARH3:ADPr (purple; this study, PDB 6HGZ). Mg^{2+} ion (cyan) and ADPr (black) from *Lch*ARH3:ADPr are given for orientation. **(D)** Comparison between *h*ARH1:ADP (PDB 3HFW, yellow) and *h*ARH1:ADPr (this study, PDB 6G28, blue) shows the coordination of a single potassium ion (purple) in the *h*ARH1:ADP complex. This is associated with the distortion of helix α2 (indicated by arrow heads). **(E and F)** Ligand coordination of *h*ARH1:ADPr (E) and *Lch*ARH3:ADPr (F). Atom are given as circles (carbon, black; oxygen, red; nitrogen, blue; phosphorus, yellow; magnesium, cyan; and waters, mauve), ligand bonds are black and protein residue bond light brown. Polar contacts are given as dotted lines with distances indicated in ångström, ligand-magnesium interactions are highlighted in purple and hydrophobic interactions are indicated by red semi-circles. Diagrams were generated with LigPlot+ (Laskowski and Swindells, 2011).

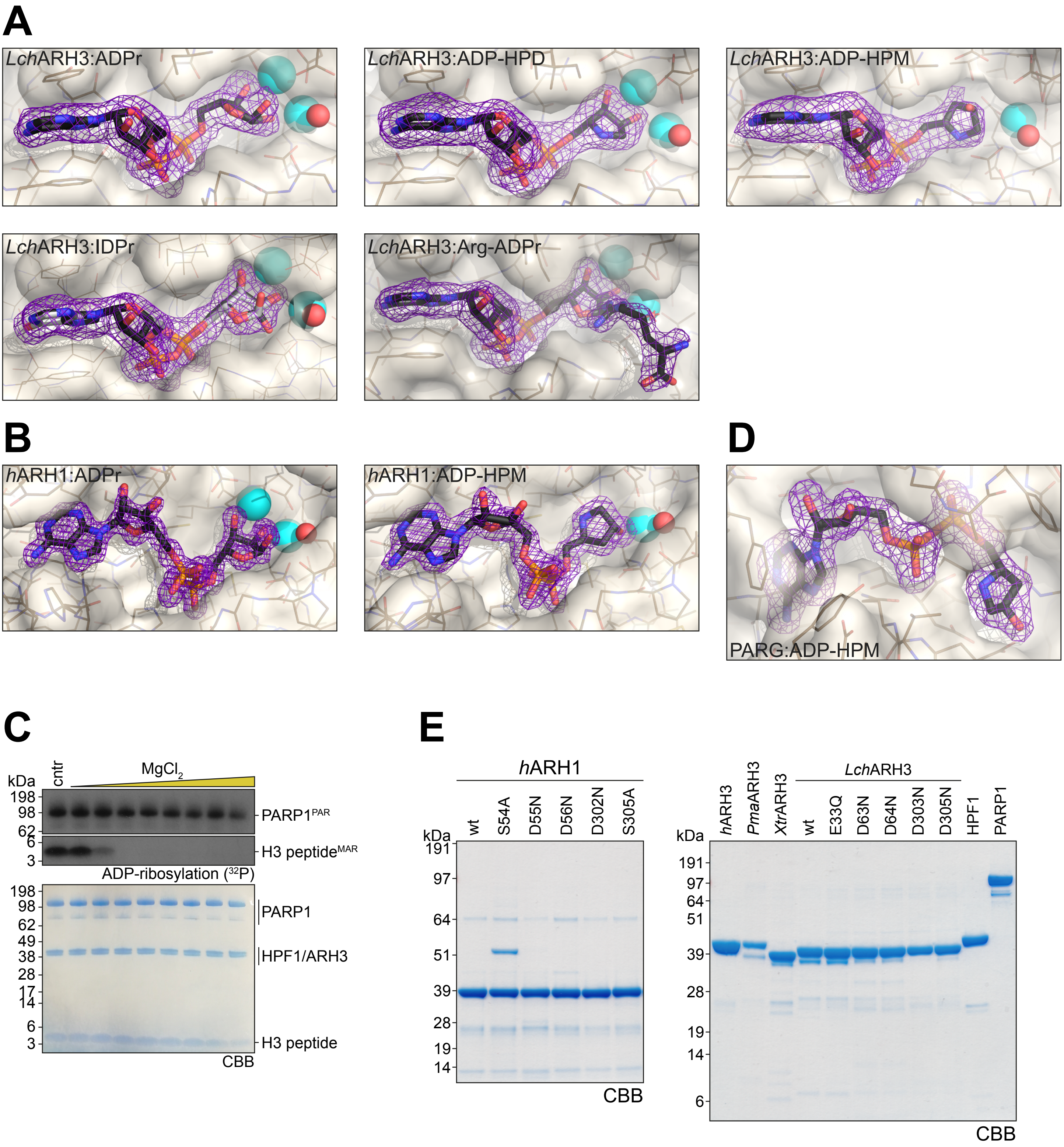

Figure S2. Quality control experiments. (Related to Figure 1, 2, S4 and STAR*Methods)
(A) Polder OMIT maps of ligands bound to *Lch*ARH3 contoured at 4 σ. Note, that the *Lch*ARH3:IDPr complex contains both the α- and β-anomer (grey and black, respectively). **(B)** Polder OMIT maps of ligands bound to *h*ARH1 contoured at 4 σ. **(C)** Reactivation of EDTA-treated *Lch*ARH3 by magnesium. The reactions were supplemented with increasing amounts of $MgCl_2$ (1, 10, 25, 50, 100, 250, 500 and 1000 µM) as indicated. **(D)** Polder OMIT maps of ADP-HPM bound to PARG contoured at 4 σ. **(E)** Protein purity was accessed by SDS-PAGE and CBB staining. The depicted gel show representative purity of single affinity batch purification of protein for biochemistry. 5 µg protein were loaded per lane.

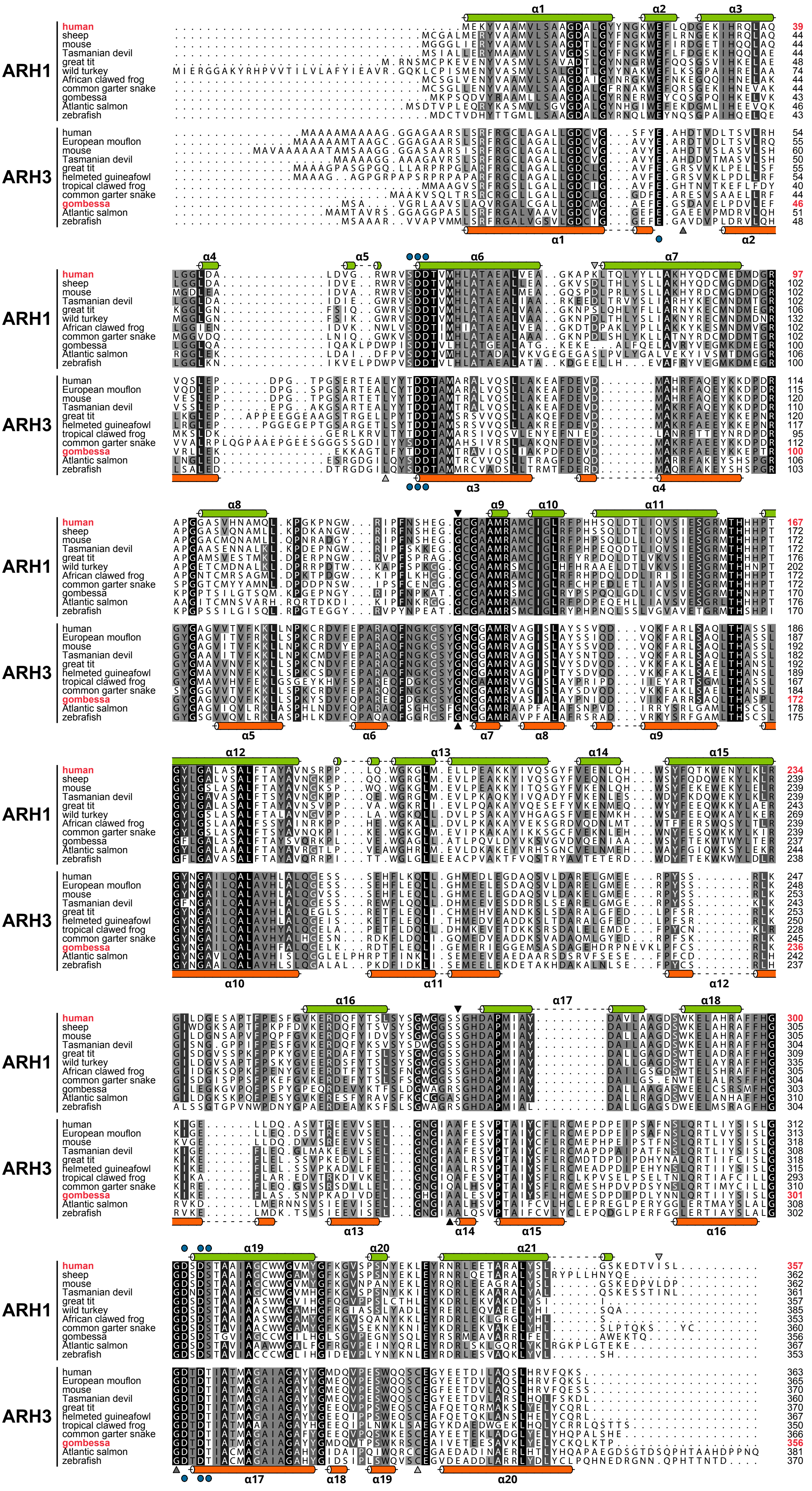

Figure S3. Multiple sequence alignment of ARH1 and ARH3. (Related to Figure 1, 2, 3 and Table S3) Sequence alignment of vertebrate ARH1 and ARH3. Sequences of proteins crystallised in this study are emphasised (red). α-helices are highlighted as tubes (*h*ARH1 green; *Lch*ARH3 orange), β-bridges as triangles (colour-matched) and magnesium coordinating residues as circles (blue) given above and below the alignment, respectively. The alignment was created with MAFFT and visualized using ALINE.

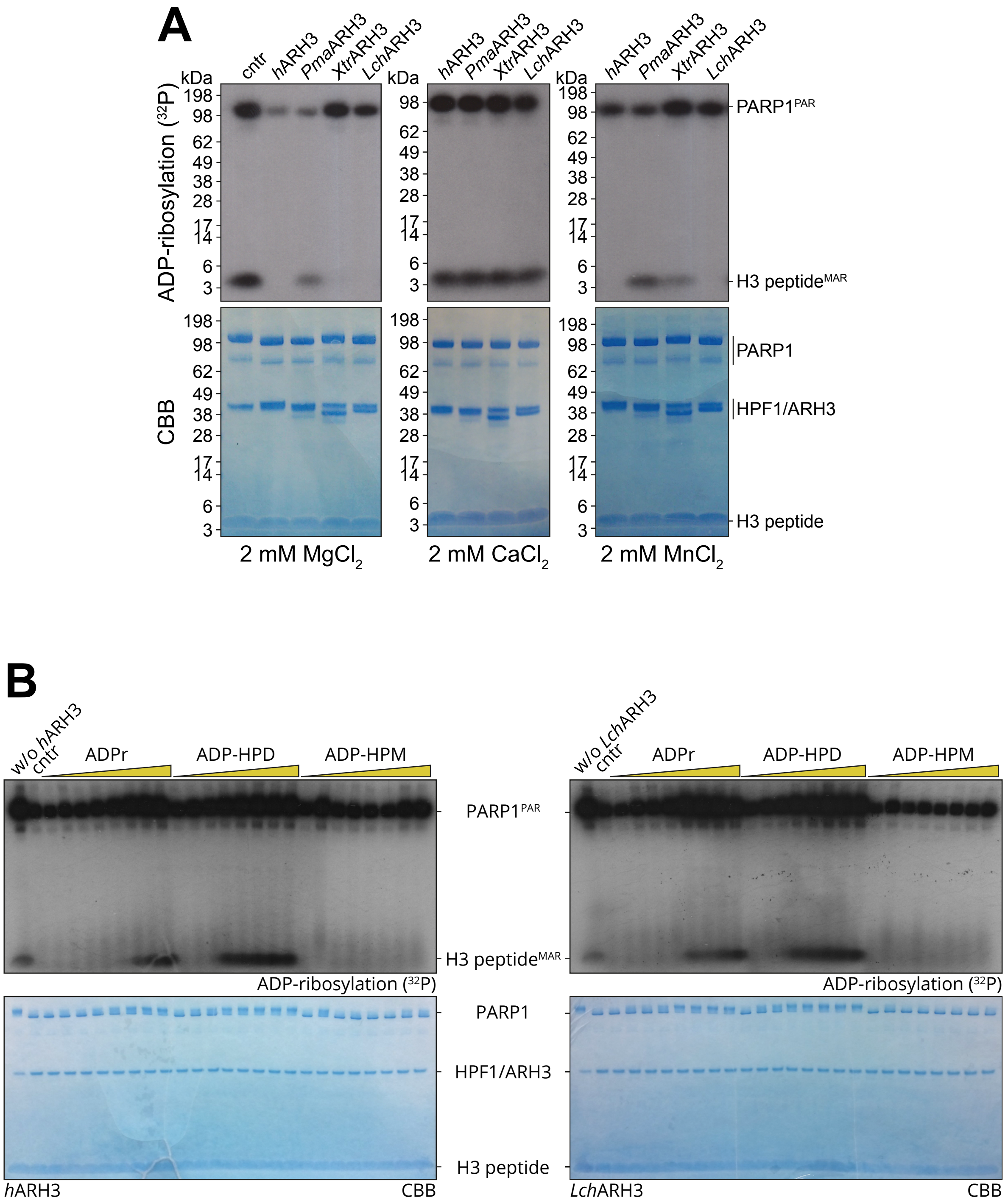

Figure S4. Evolutionary conservation of metal preference and inhibitor sensitivity. (Related to Figure 2 and 3) **(A)** Activity assay with the indicated ARH3 homologues were performed in presence of 2 mM $MgCl_2$, $CaCl_2$, or $MnCl_2$. **(B)** Analysis of *h*ARH3 (left panel) and *Lch*ARH3 (right panel) inhibition by ADPr, ADP-HPD and ADP-HPM. Histone H3 peptide (aa 1-20) was serine ADP-ribosylated by PARP1 in presence of HPF1. The reaction was stopped with olaparib and supplemented with wt ARH3 and increasing amounts of compound (1, 5, 10, 50, 100, 250, 500 and 1000 µM) as indicated.

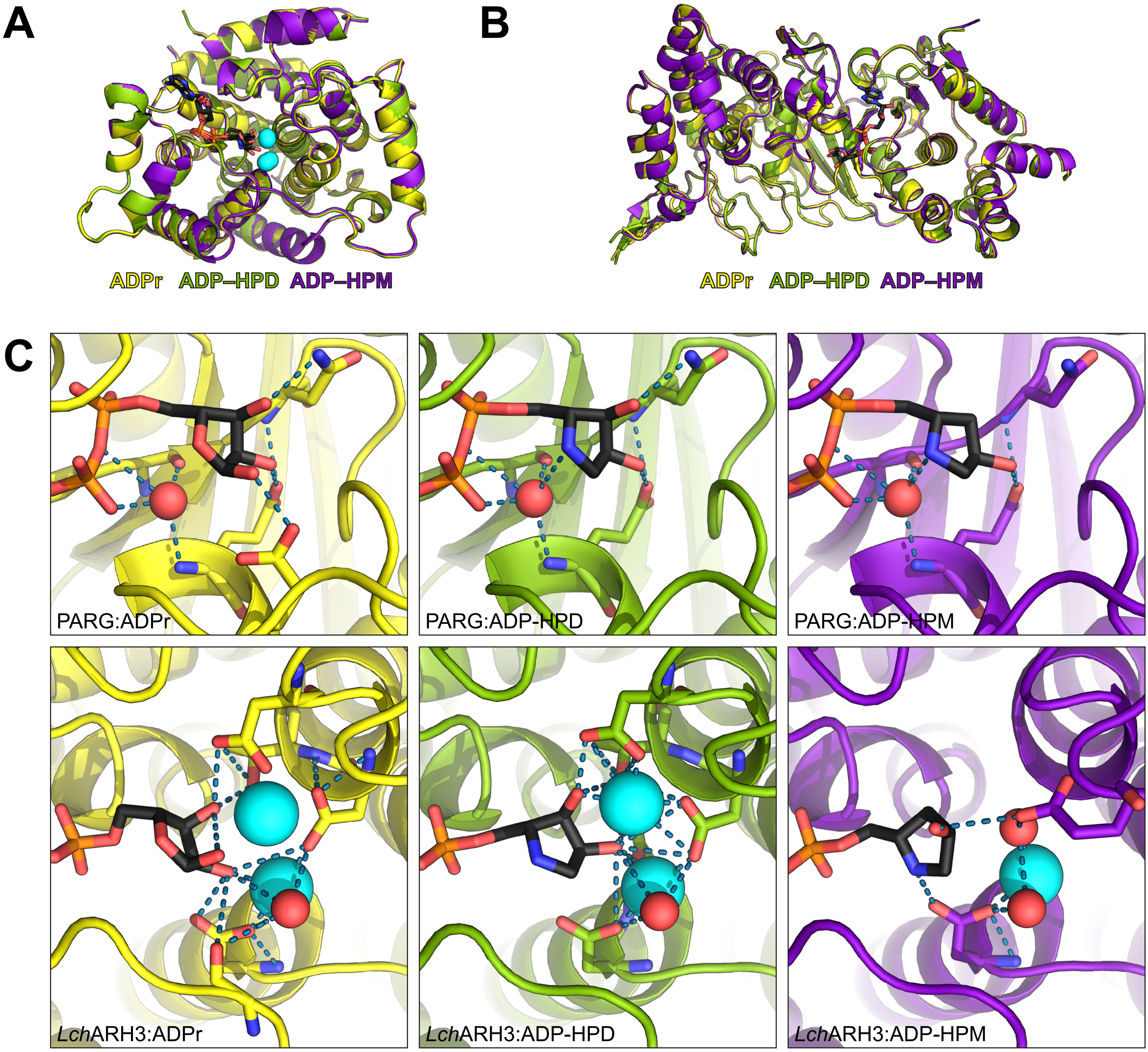

Figure S5. Comparison of the coordination of ADPr, ADP-HPD and ADP-HPM by PARG and *Lch*ARH3.
(Related to Figure 4 and Discussion)
(A) Overall ribbon representation of *Lch*ARH3 in complex with ADPr, ADP-HPD and ADP-HPM. The protein is coloured according to ligand (ADPr, yellow; ADP-HPD, green; ADP-HPM, purple) and Mg^{2+} ions are given in cyan. **(B)** Overall ribbon representation of PARG in complex with ADPr (PDB 4B1H), ADP-HPD (PDB 4B1J) and ADP-HPM (this study). Protein is coloured as indicated in (A). **(C)** Close-up of the coordination of the distal ribose (ADPr) or pyrrolidine ring (ADP-HPD and -HPM). The upper panels show PARG and the lower panels *Lch*ARH3. Protein is coloured as indicated in (A) with addition of magnesium coordinating waters (red) and polar contacts (blue).

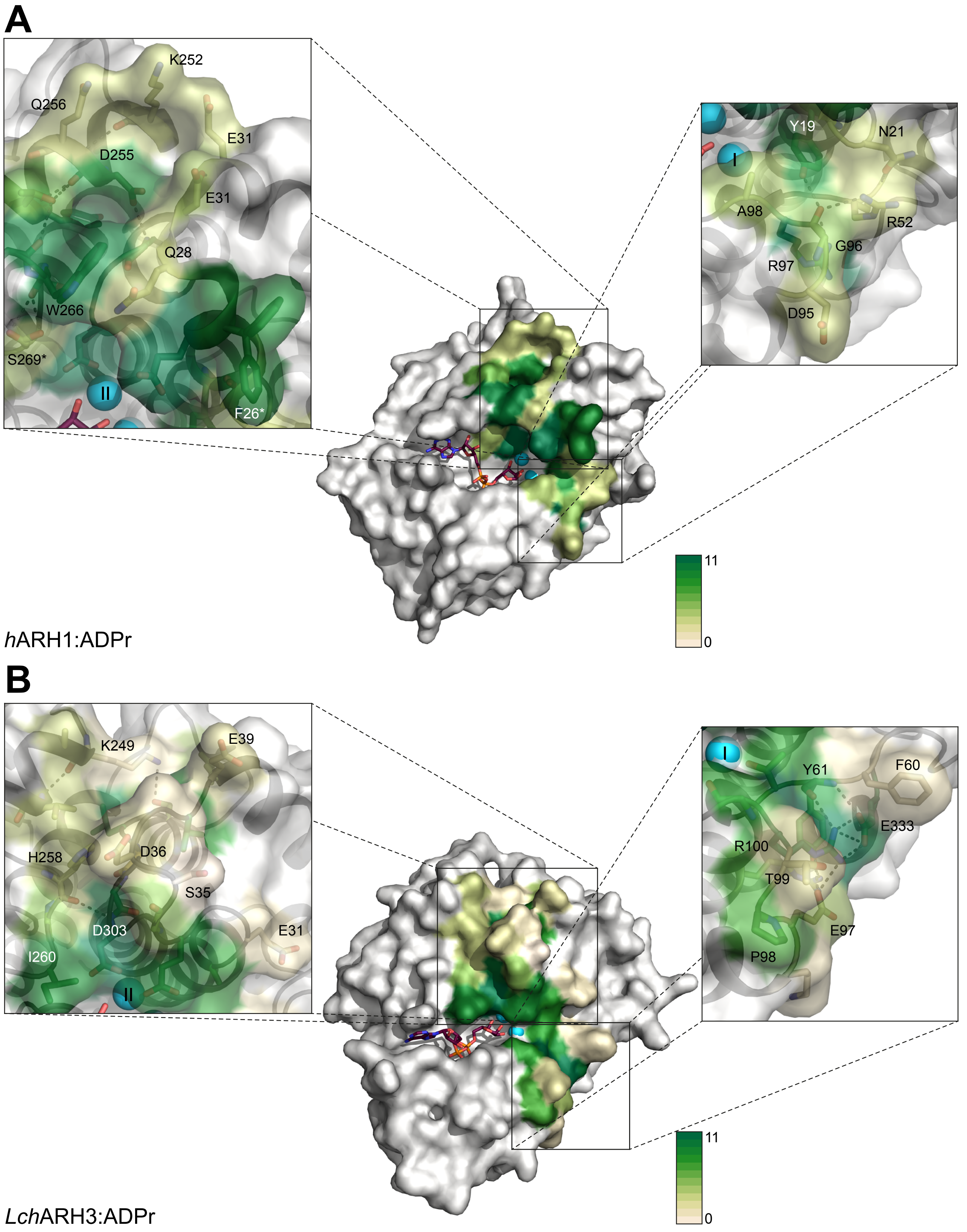

Figure S6. Potential substrate binding surfaces. (Related to Figure 2)
Surface representation of *h*ARH1 **(A)** and *Lch*ARH3 **(B)**. The potential substrate binding surfaces are highlighted according to physicochemical conservation (scale 0-10 for property conservation and 11 for residue conservation) (Livingstone and Barton, 1993). The bound ADPr (purple) and magnesium ions (cyan) are given for orientation. Close-up contain in addition ribbon-liquorice representation of residues forming the potential binding surfaces and selected polar contacts are indicated as dotted lines.

A

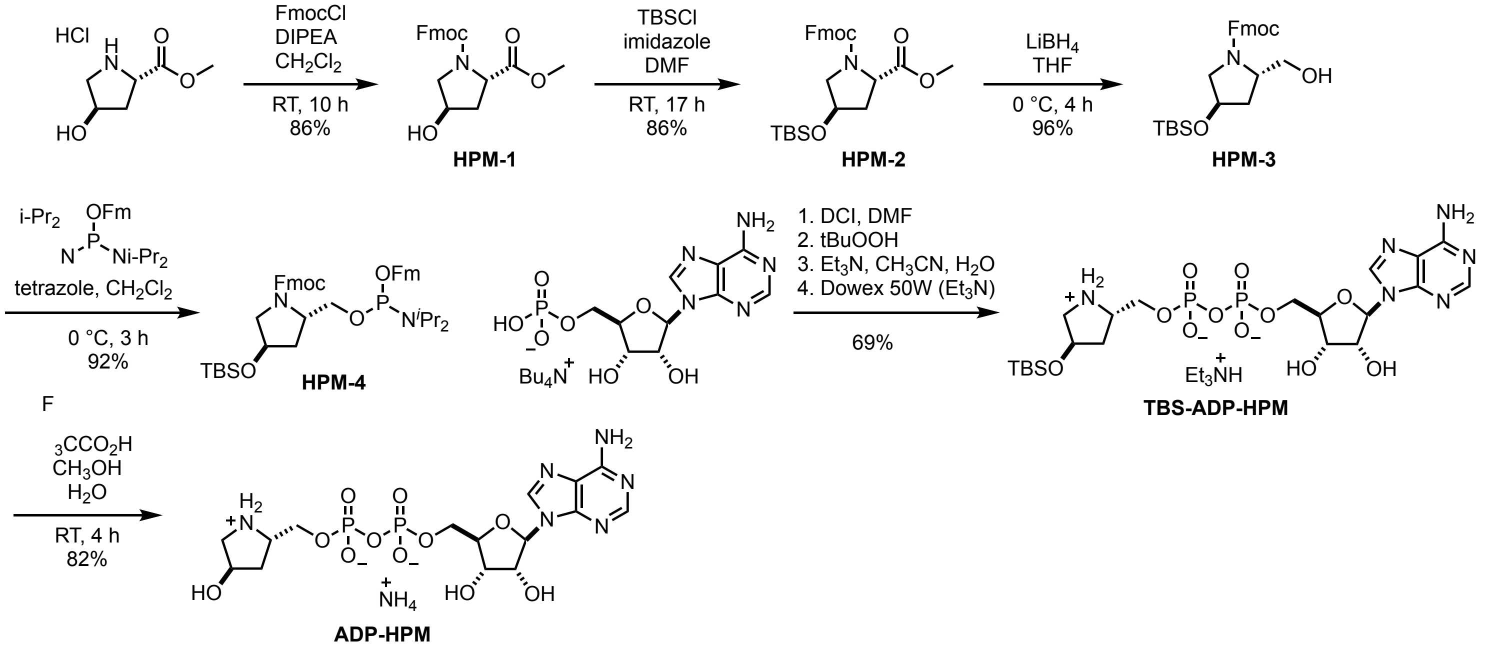

B

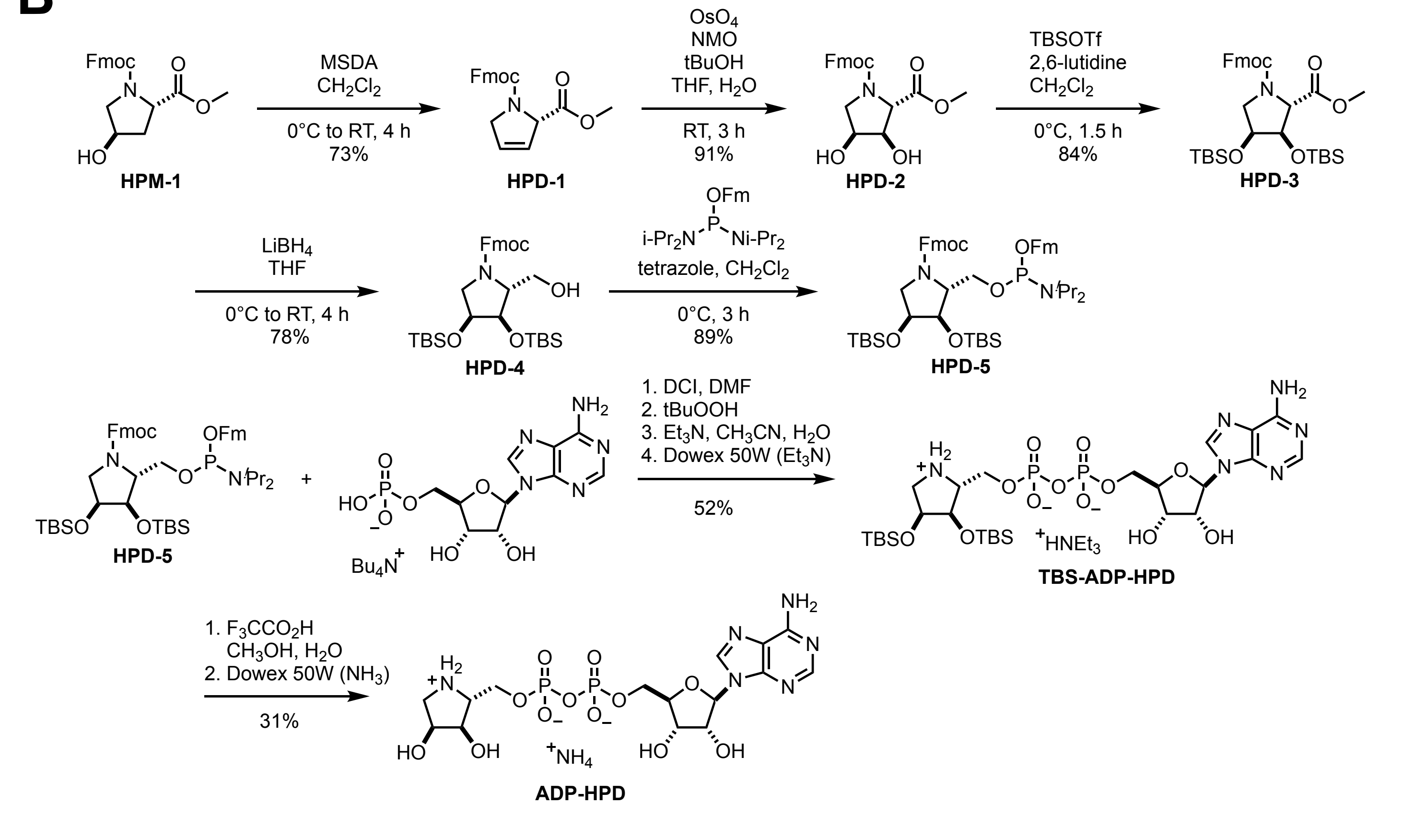

Figure S7. Chemical synthesis schemes. (Related to STAR*Methods)
(A) Synthesis scheme of ADP-HPM.
(B) Synthesis scheme of ADP-HPD.
