# Supplementary material for: (ADP-ribosyl)hydrolases: Structural Basis for Differential Substrate Recognition and Inhibition
Source: Cell Chem Biol. 2018 Dec 20;25(12):1533–1546.e12. doi: 10.1016/j.chembiol.2018.11.001 (PMC6309922; doi:10.1016/j.chembiol.2018.11.001)
Supplement: Data S1. NMR Spectra Concerning the Synthesis of ADP-HPM, Related to STAR Methods [file mmc2.pdf]

Data S1. NMR spectra concerning the synthesis of ADP-HPM (Related to STAR*Methods)

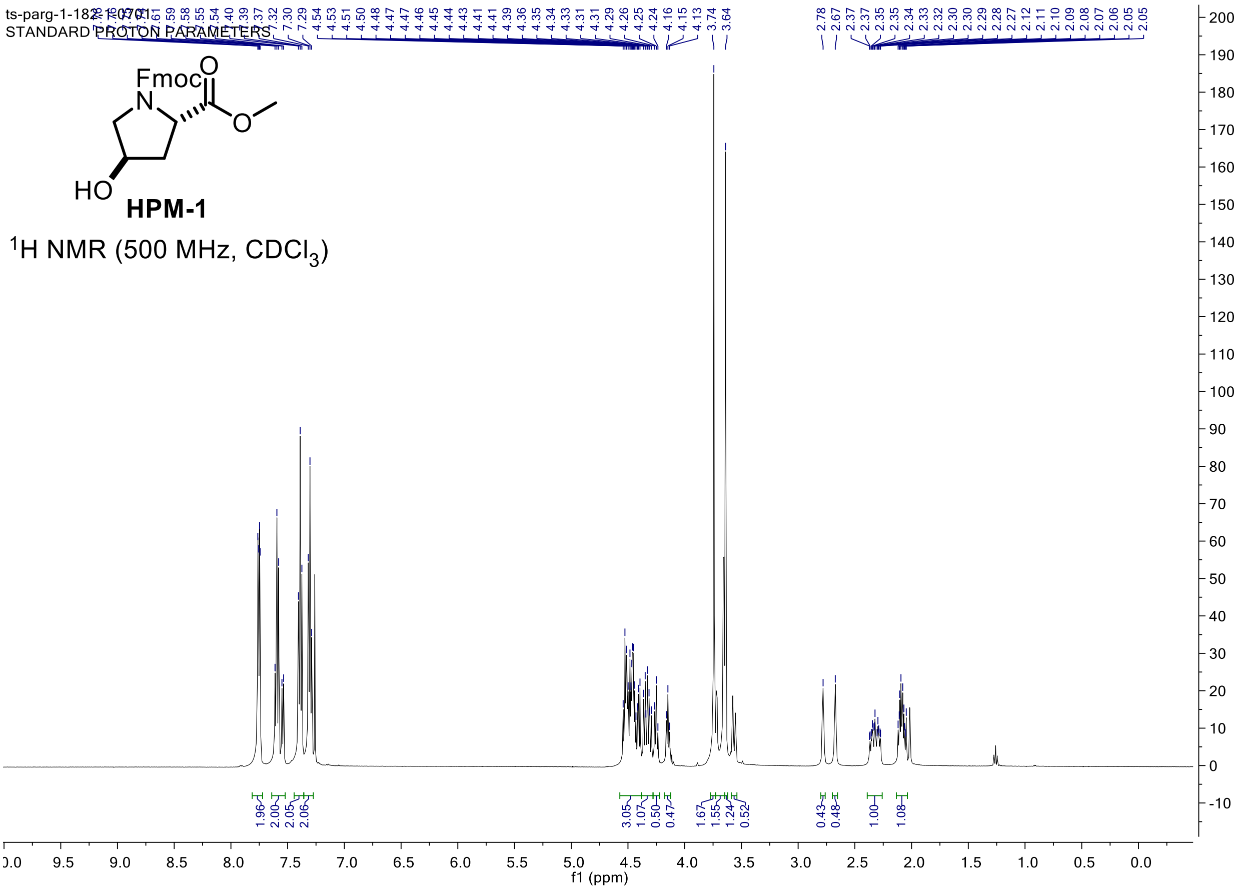

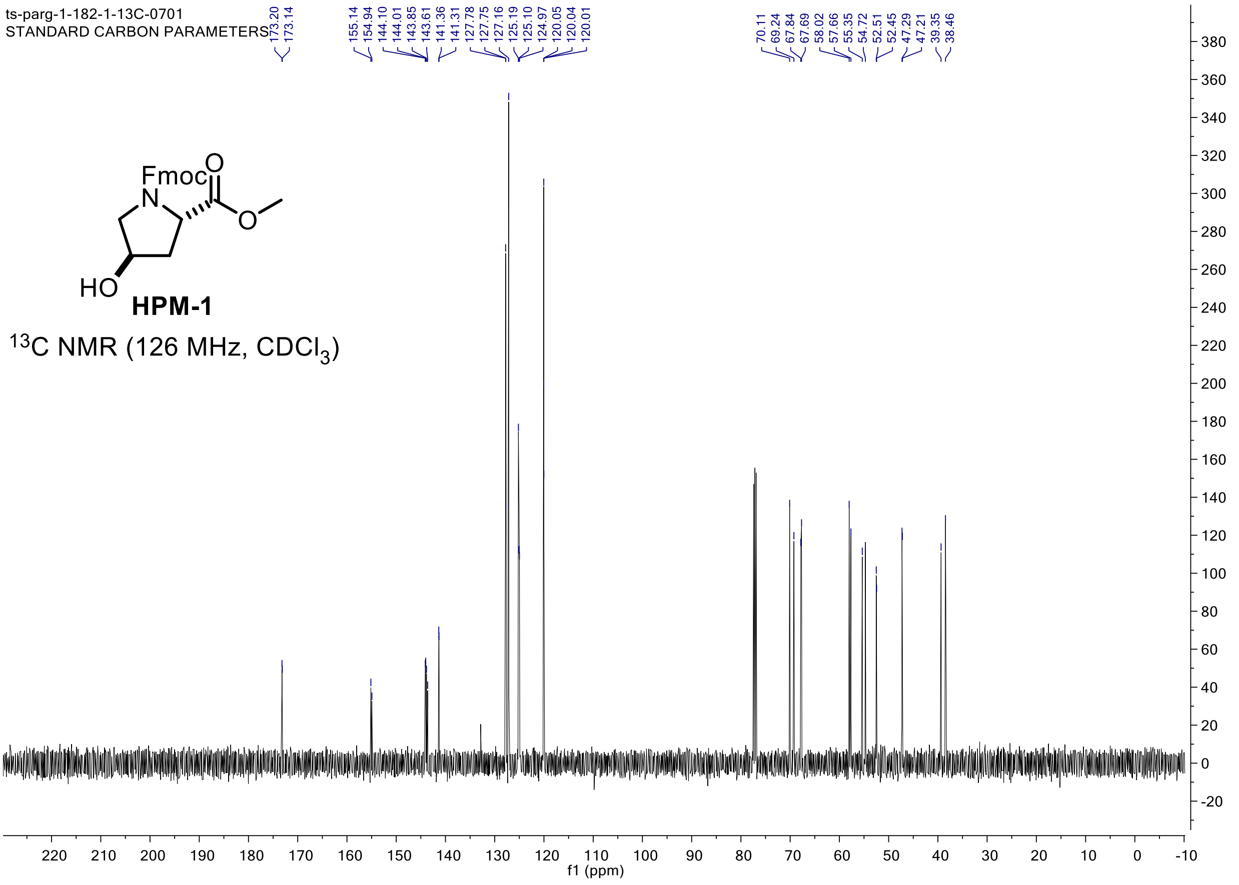

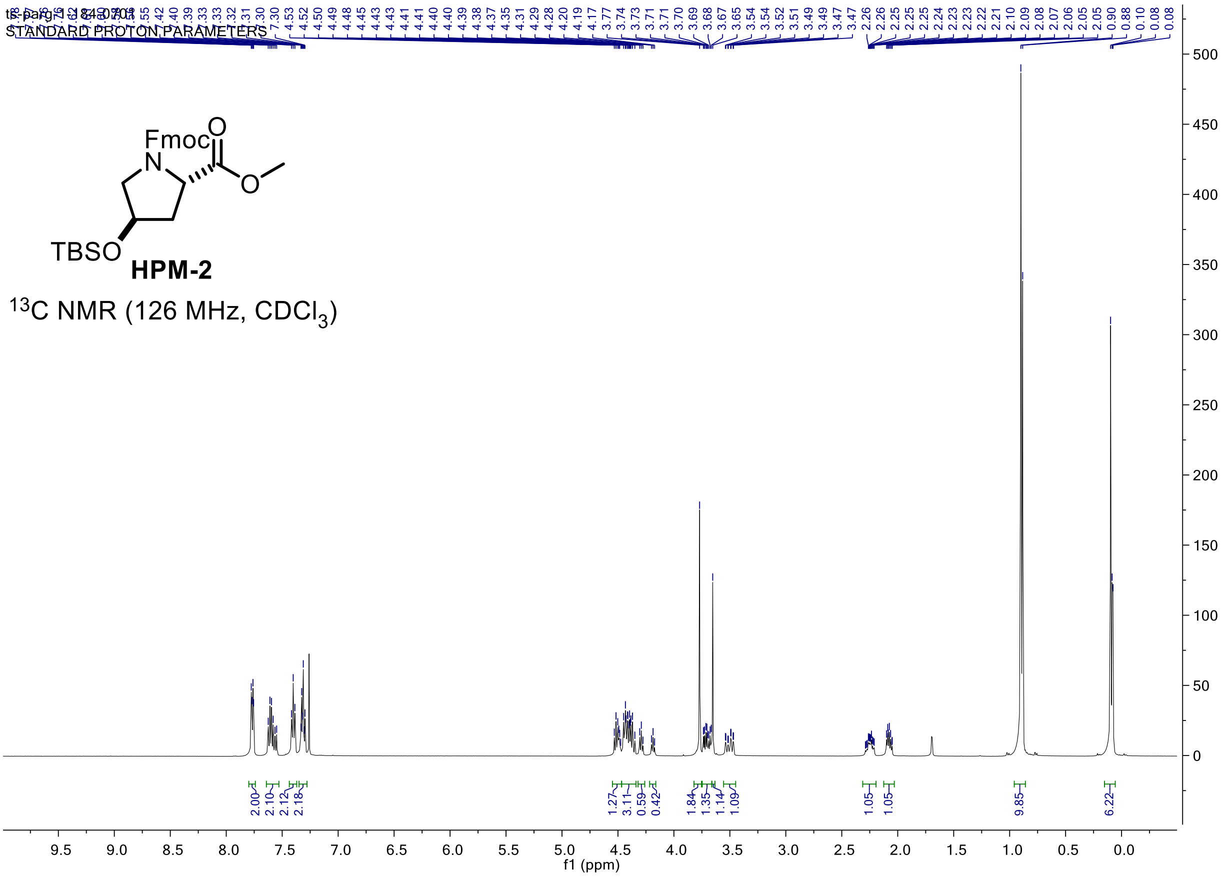
ts-parg-1-184-1H-0701
STANDARD PROTON PARAMETERS
Fmoc
N
O
O
TBSO
HPM-2
13C NMR (126 MHz, CDCl3)
7.55 7.42 7.40 7.39 7.33 7.33 7.32 7.31 7.30 4.53 4.52 4.50 4.49 4.48 4.45 4.43 4.43 4.41 4.41 4.40 4.40 4.39 4.38 4.37 4.35 4.31 4.29 4.28 4.20 4.19 4.17 3.77 3.74 3.73 3.71 3.71 3.70 3.69 3.68 3.67 3.65 3.54 3.54 3.52 3.51 3.49 3.49 3.47 3.47 2.26 2.26 2.25 2.25 2.25 2.24 2.23 2.23 2.22 2.21 2.10 2.09 2.08 2.07 2.06 2.05 2.05 0.90 0.88 0.10 0.08 0.08
2.00 2.10 2.12 2.18 1.27 3.11 0.59 0.42 1.84 1.35 1.14 1.09 1.05 1.05 9.85 6.22
f1 (ppm)

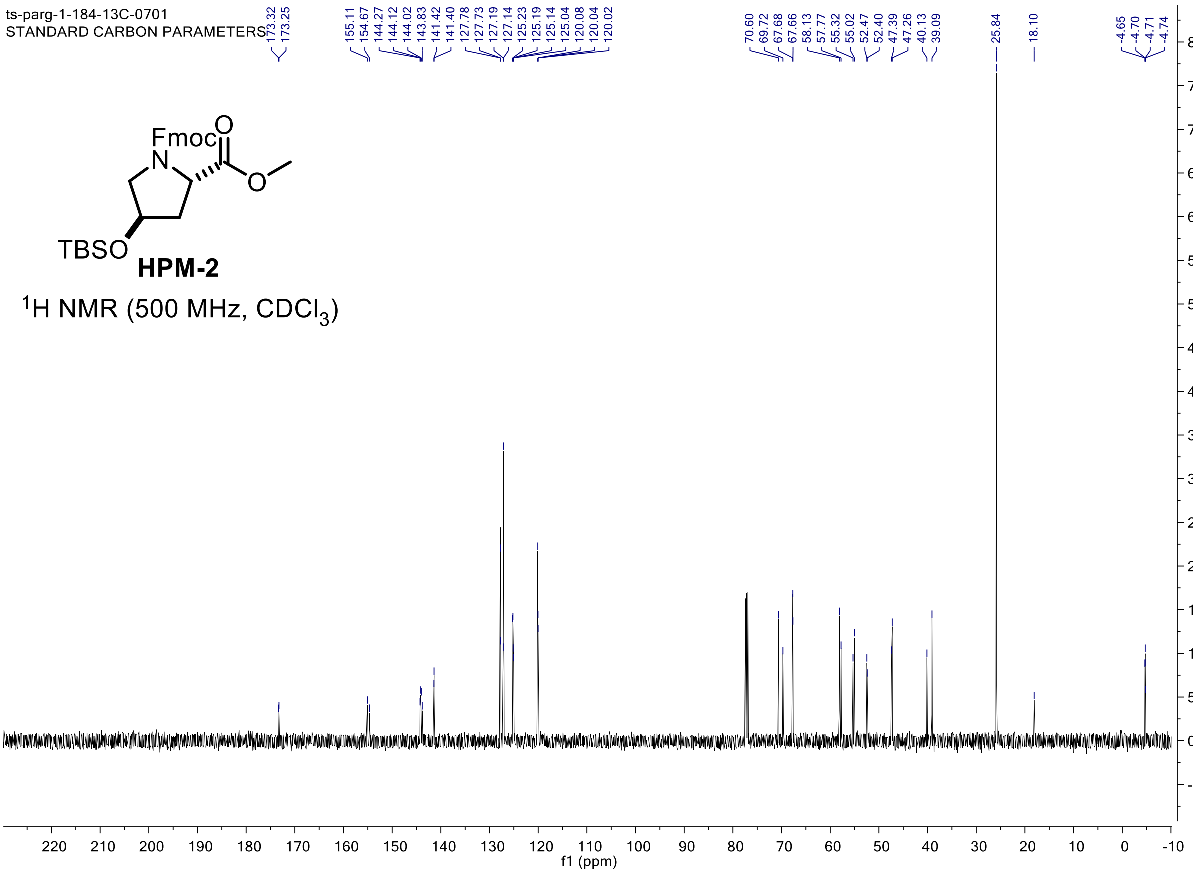
ts-parg-1-184-13C-0701
STANDARD CARBON PARAMETERS
Fmoc
N
O
O
TBSO
HPM-2
1H NMR (500 MHz, CDCl3)
173.32 173.25 155.11 154.67 144.27 144.12 144.02 143.83 141.42 141.40 127.78 127.73 127.19 127.14 125.23 125.19 125.14 125.04 120.08 120.04 120.02 70.60 69.72 67.68 67.66 58.13 57.77 55.32 55.02 52.47 52.40 47.39 47.26 40.13 39.09 25.84 18.10 -4.65 -4.70 -4.71 -4.74
f1 (ppm)

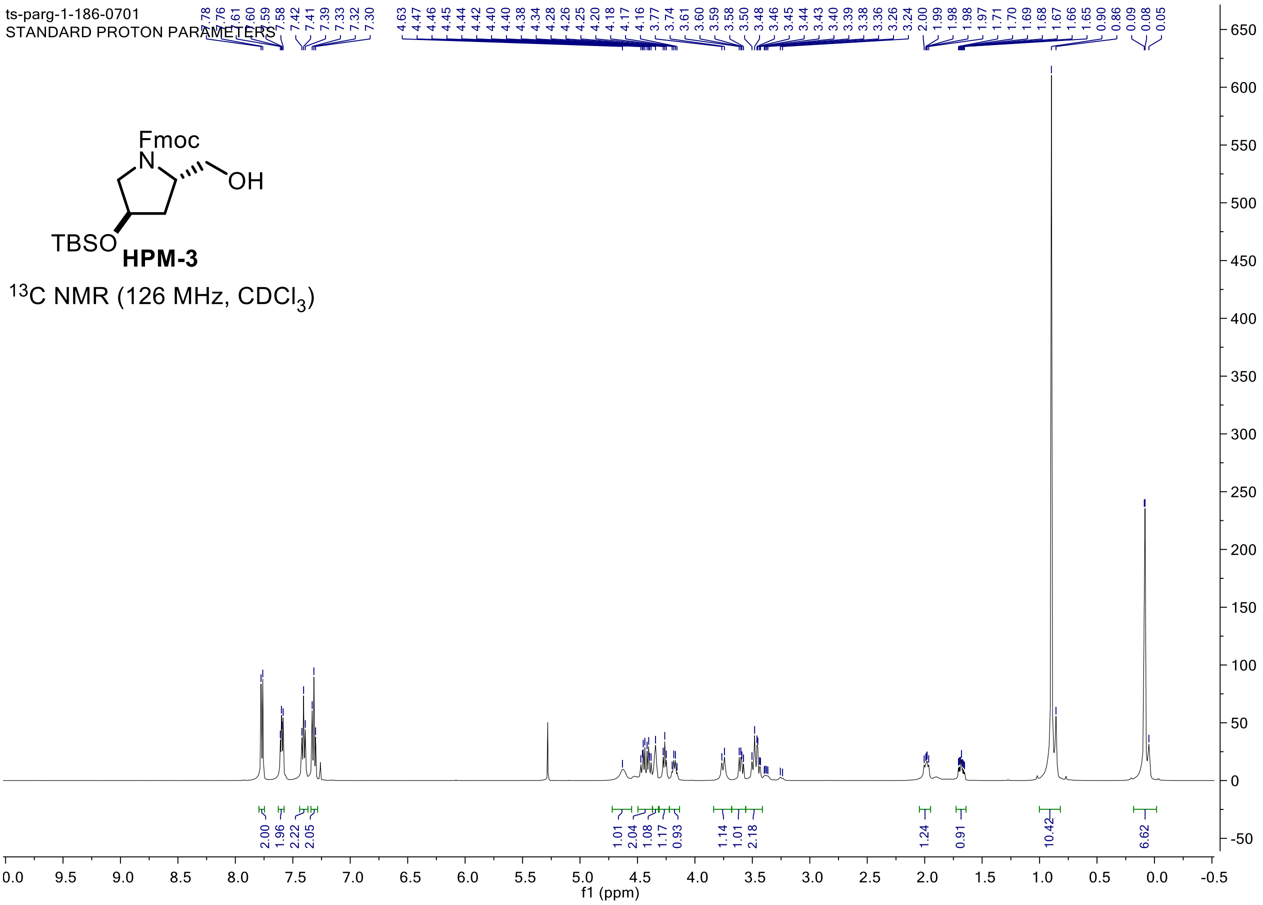
ts-parg-1-186-0701
STANDARD PROTON PARAMETERS
7.78 7.76 7.61 7.60 7.59 7.58 7.42 7.41 7.39 7.33 7.32 7.30
4.63 4.47 4.46 4.45 4.44 4.42 4.40 4.40 4.38 4.34 4.28 4.26 4.25 4.20 4.18 4.17 4.16 3.77 3.74 3.61 3.60 3.59 3.58 3.50 3.48 3.46 3.45 3.44 3.43 3.40 3.39 3.38 3.36 3.26 3.24 2.00 1.99 1.98 1.98 1.97 1.71 1.70 1.69 1.68 1.67 1.66 1.65 0.90 0.86 0.09 0.08 0.05
Fmoc
N
OH
TBSO
HPM-3
^{13}C NMR (126 MHz, CDCl$_3$)
2.00 1.96 2.22 2.05 1.01 2.04 1.08 1.17 0.93 1.14 1.01 2.18 1.24 0.91 10.42 6.62
f1 (ppm)

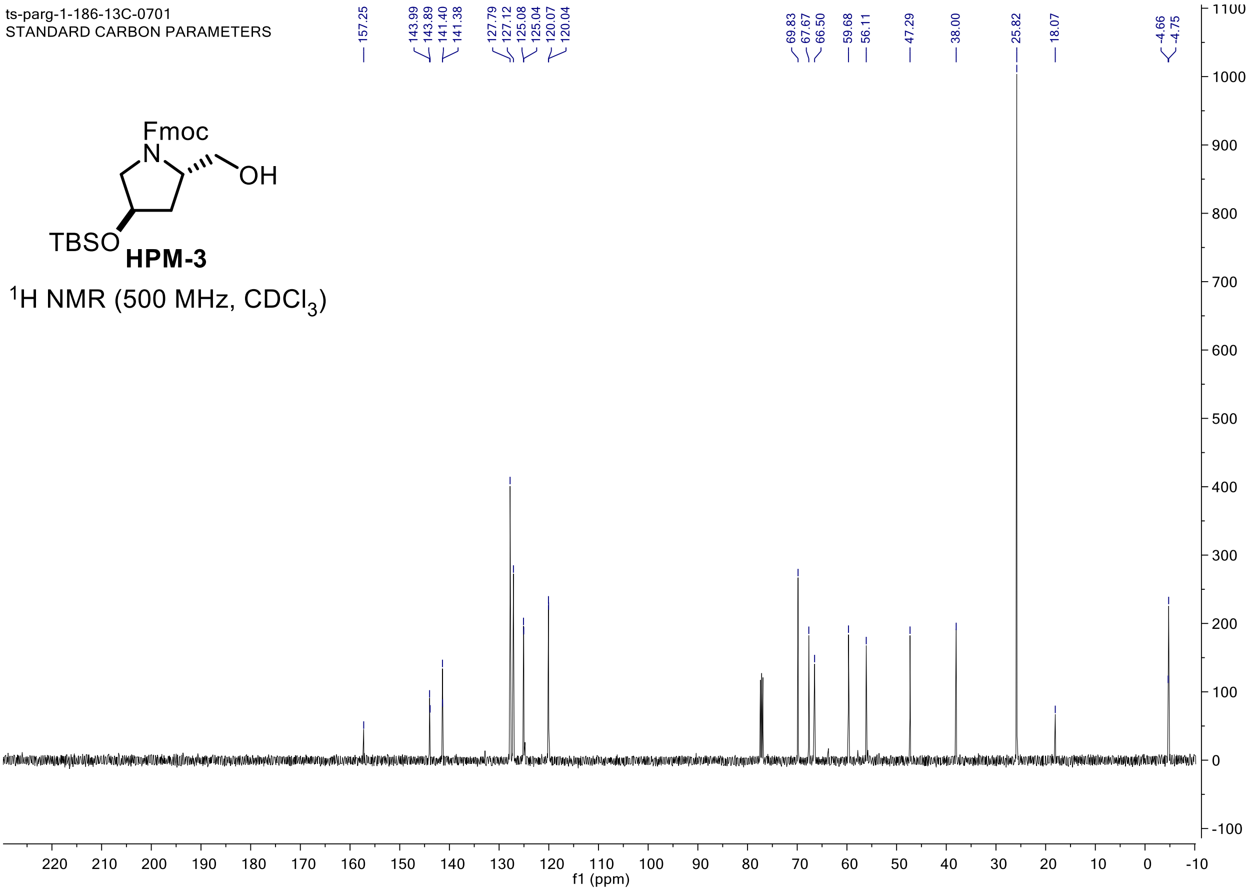
ts-parg-1-186-13C-0701
STANDARD CARBON PARAMETERS
157.25
143.99 143.89 141.40 141.38
127.79 127.12 125.08 125.04 120.07 120.04
69.83 67.67 66.50 59.68 56.11 47.29 38.00 25.82 18.07 -4.66 -4.75
Fmoc
N
OH
TBSO
HPM-3
^{1}H NMR (500 MHz, CDCl$_3$)
f1 (ppm)

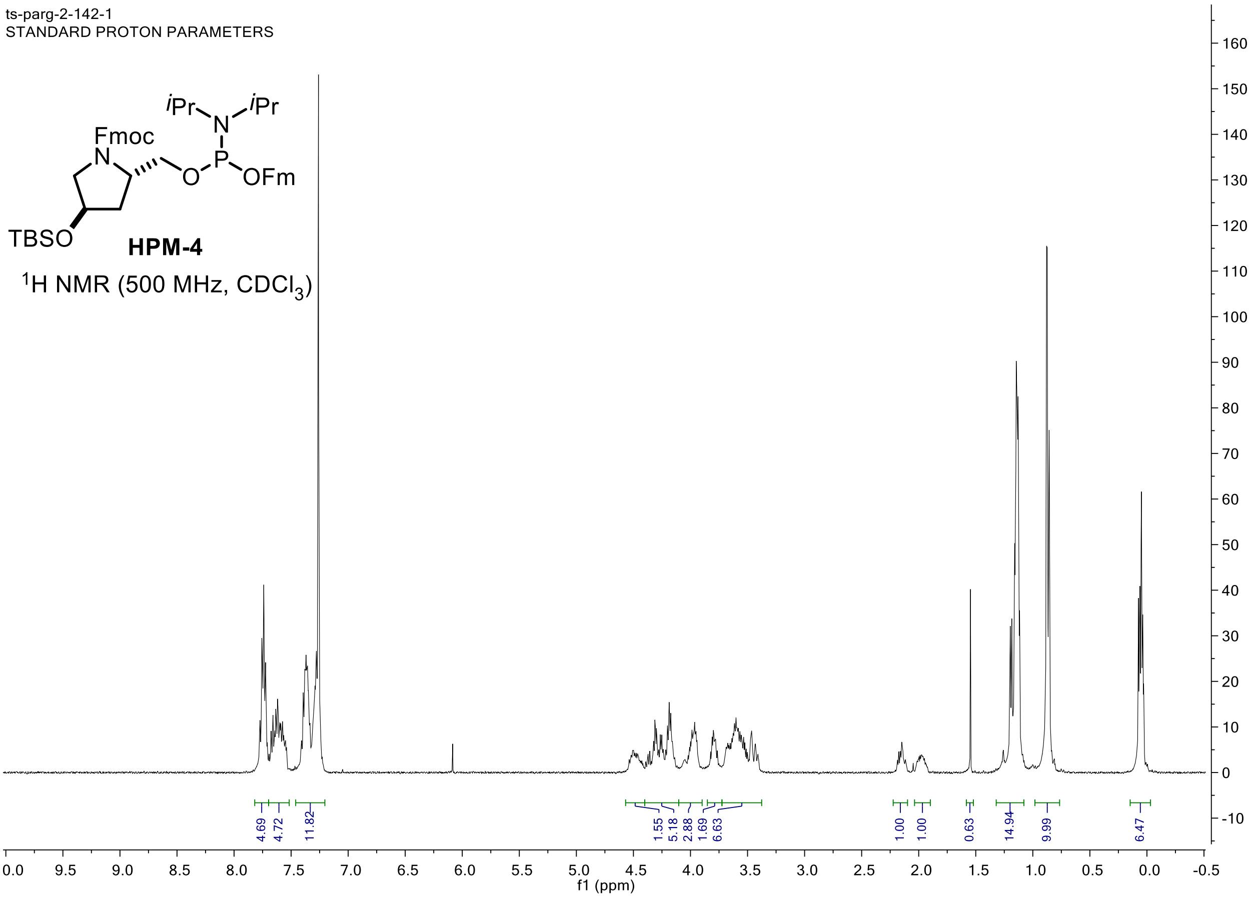
ts-parg-2-142-1
STANDARD PROTON PARAMETERS
iPr N iPr
Fmoc
N
O P OFm
TBSO
HPM-4
1H NMR (500 MHz, CDCl3)
4.69 4.72 11.82 1.55 5.18 2.88 1.69 6.63 1.00 1.00 0.63 14.94 9.99 6.47
f1 (ppm)

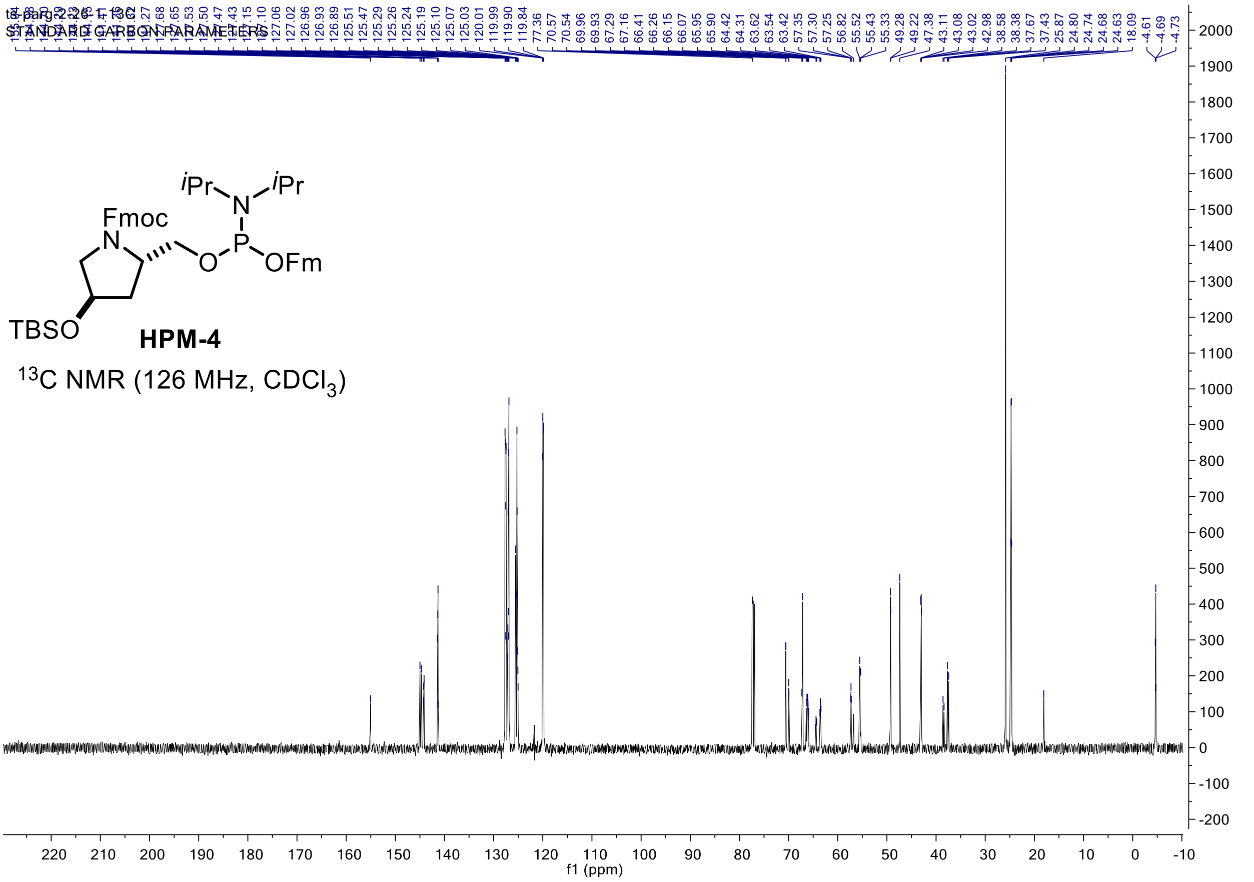
STANDARD CARBON PARAMETERS
127.68 127.65 127.53 127.50 127.47 127.43 127.15 127.10 127.06 127.02 126.96 126.93 126.89 125.51 125.47 125.29 125.26 125.24 125.19 125.10 125.07 125.03 120.01 119.99 119.90 119.84 77.36 70.57 70.54 69.96 69.93 67.29 67.16 66.41 66.26 66.15 66.07 65.95 65.90 64.42 64.31 63.62 63.54 63.42 57.35 57.30 57.25 56.82 55.52 55.43 55.33 49.28 49.22 47.38 43.11 43.08 43.02 42.98 38.58 38.38 37.67 37.43 25.87 24.80 24.74 24.68 24.63 18.09 -4.61 -4.69 -4.73
iPr N iPr
Fmoc
N
O P OFm
TBSO
HPM-4
13C NMR (126 MHz, CDCl3)
f1 (ppm)

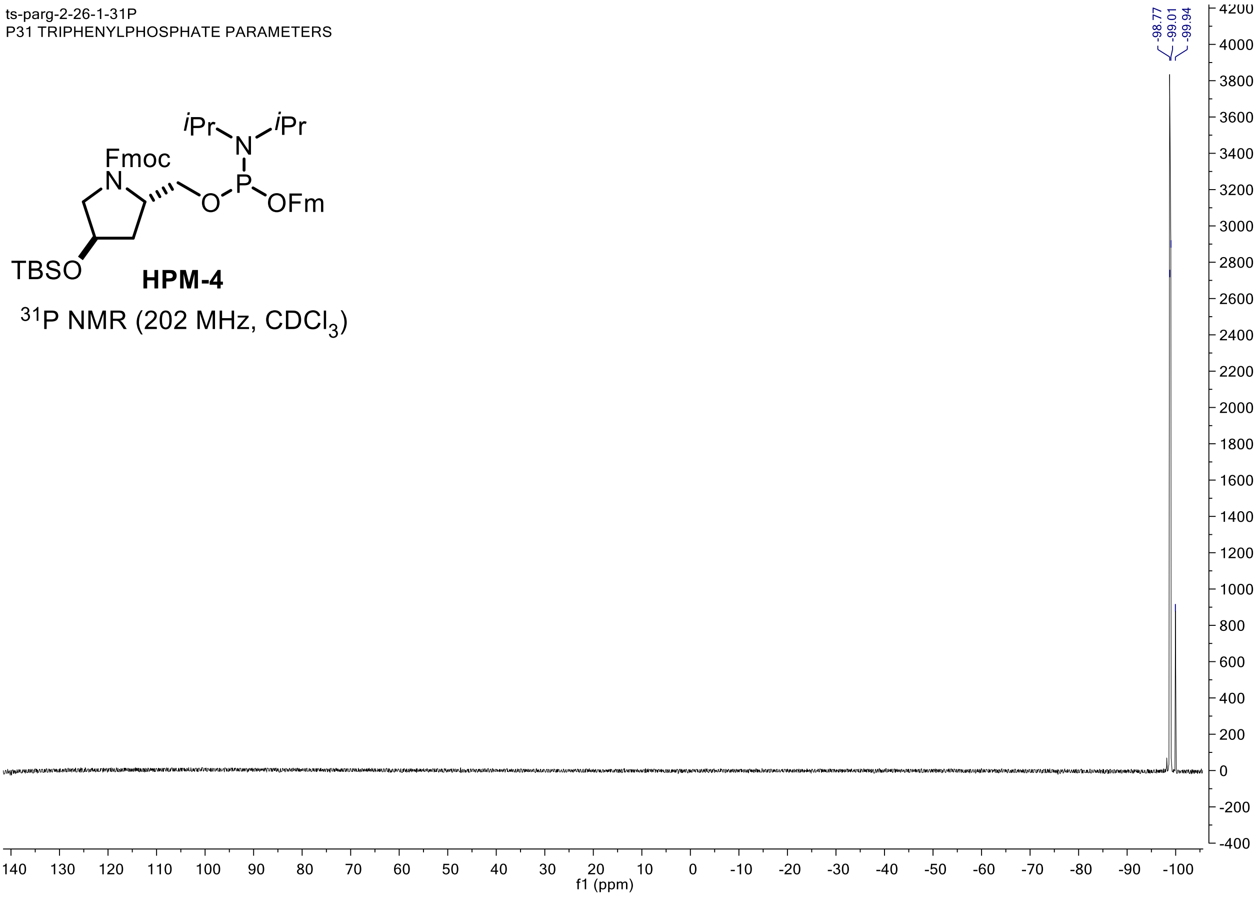
ts-parg-2-26-1-31P
P31 TRIPHENYLPHOSPHATE PARAMETERS
Fmoc
TBSO
OFm
HPM-4
31P NMR (202 MHz, CDCl3)
-98.77
-99.01
-99.94
f1 (ppm)

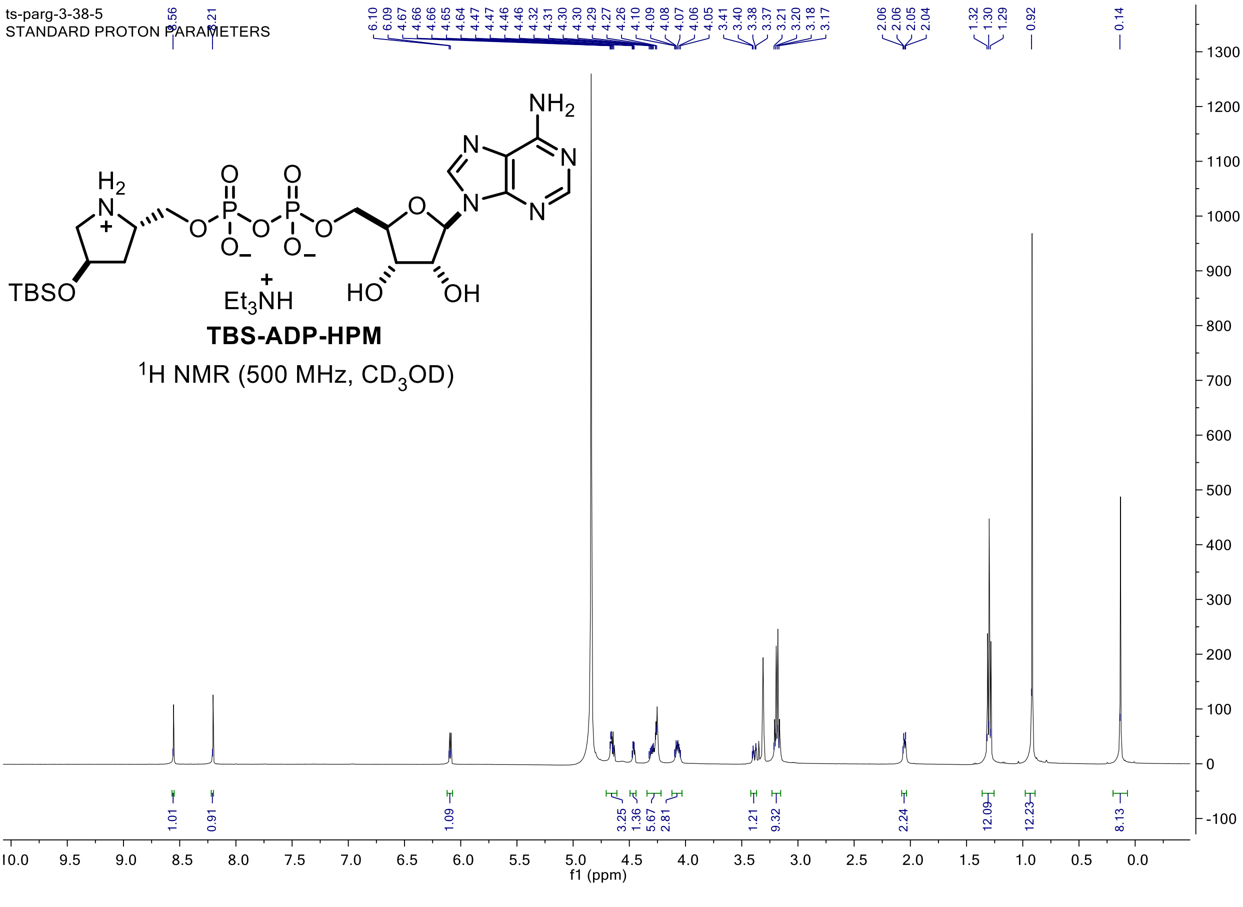
ts-parg-3-38-5
STANDARD PROTON PARAMETERS
NH2
TBSO
Et3NH
HO
OH
TBS-ADP-HPM
1H NMR (500 MHz, CD3OD)
8.56
8.21
6.10
6.09
4.67
4.66
4.66
4.65
4.64
4.47
4.47
4.46
4.32
4.31
4.30
4.30
4.29
4.27
4.26
4.10
4.09
4.08
4.07
4.06
4.05
3.41
3.40
3.38
3.37
3.21
3.20
3.18
3.17
2.06
2.06
2.05
2.04
1.32
1.30
1.29
0.92
0.14
1.01
0.91
1.09
3.25
1.36
5.67
2.81
1.21
9.32
2.24
12.09
12.23
8.13
f1 (ppm)

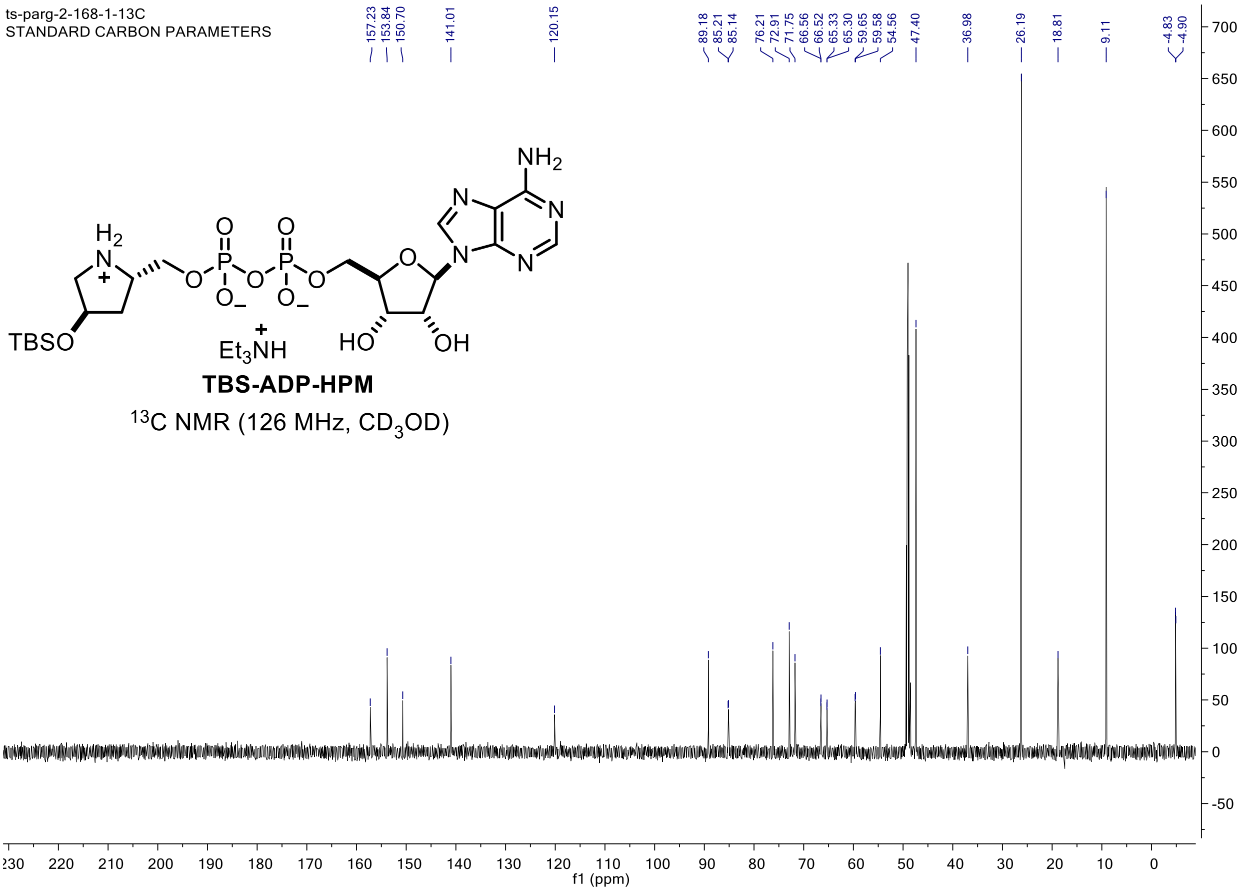
ts-parg-2-168-1-13C
STANDARD CARBON PARAMETERS
157.23
153.84
150.70
141.01
120.15
89.18
85.21
85.14
76.21
72.91
71.75
66.56
66.52
65.33
65.30
59.65
59.58
54.56
47.40
36.98
26.19
18.81
9.11
-4.83
-4.90
TBS-ADP-HPM
^{13}C NMR (126 MHz, CD_3OD)
f1 (ppm)

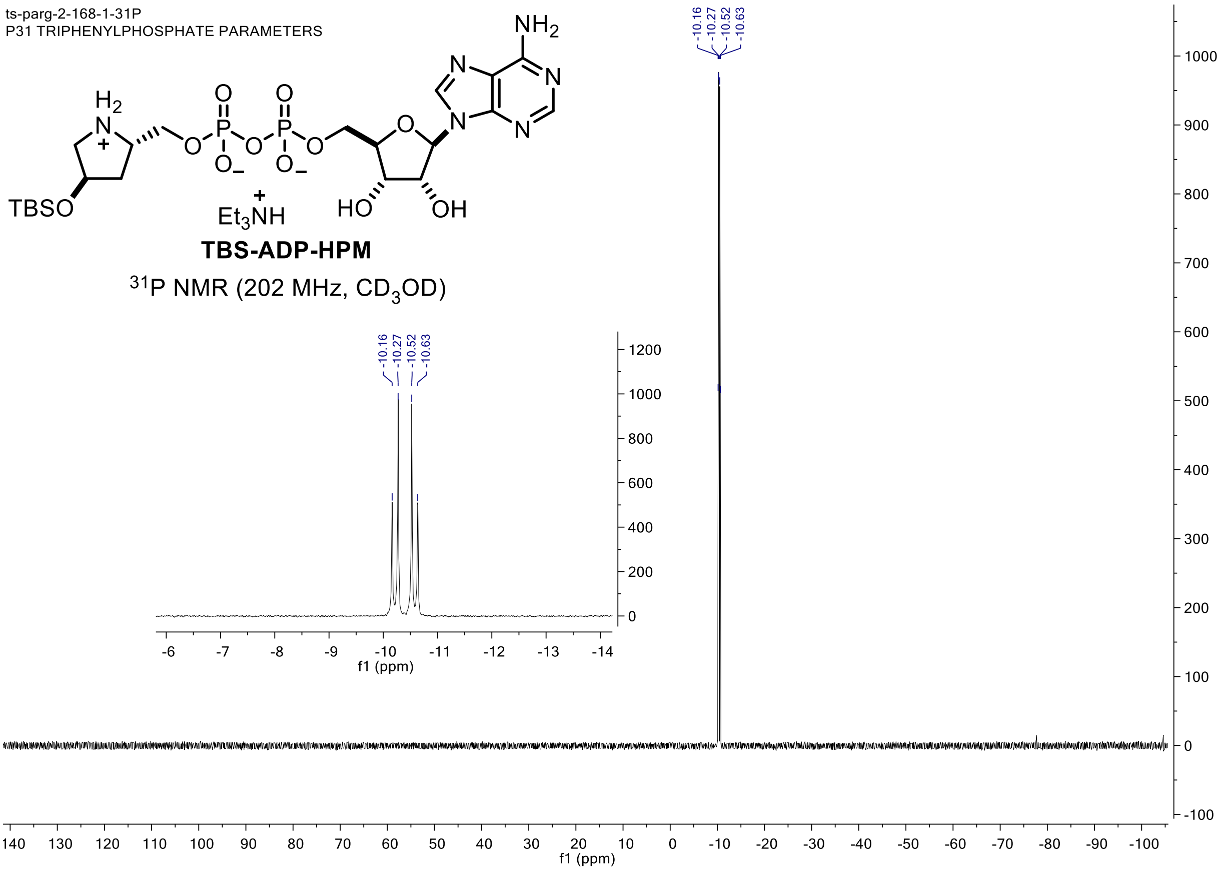
ts-parg-2-168-1-31P
P31 TRIPHENYLPHOSPHATE PARAMETERS
-10.16
-10.27
-10.52
-10.63
TBS-ADP-HPM
^{31}P NMR (202 MHz, CD_3OD)
f1 (ppm)

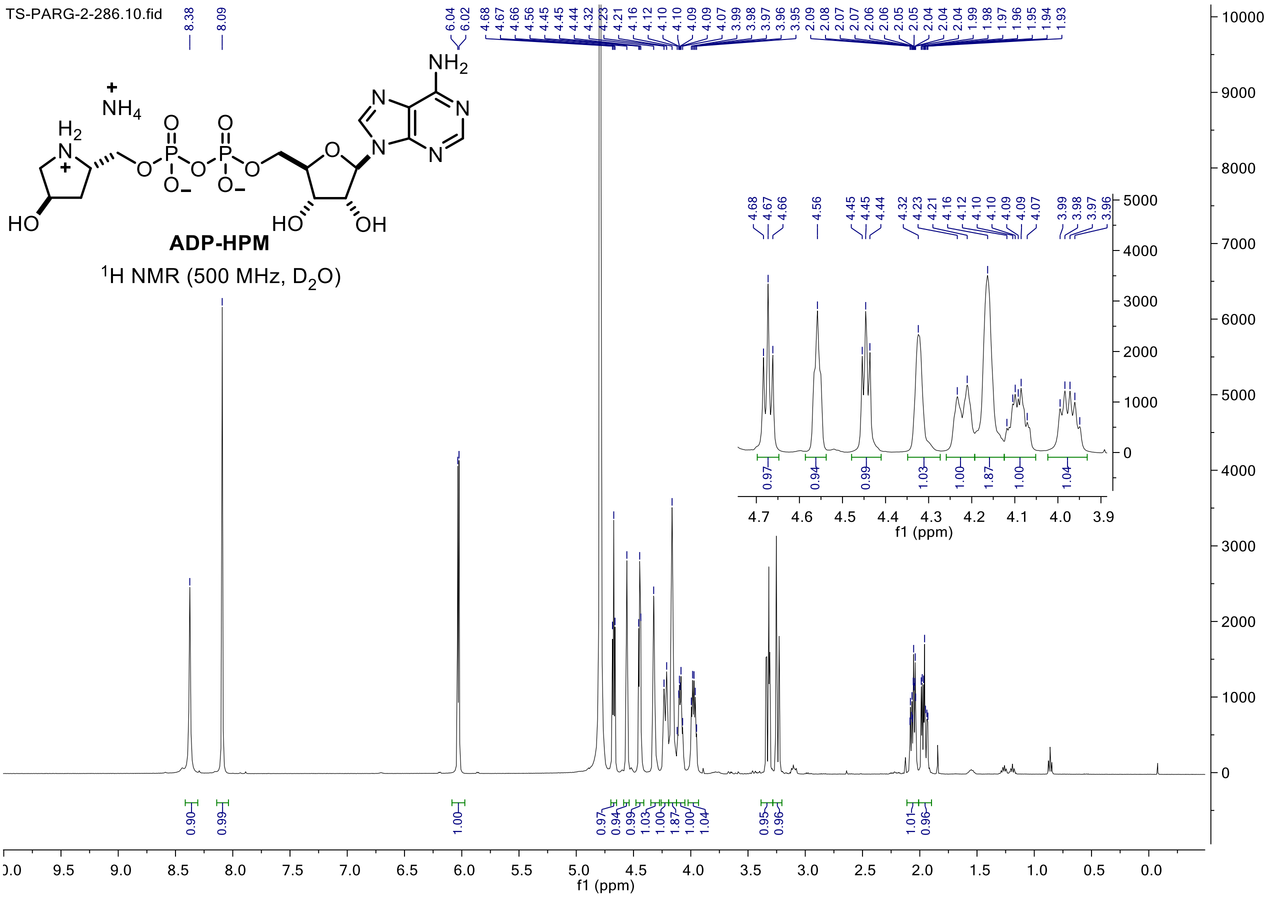
TS-PARG-2-286.10.fid
ADP-HPM
1H NMR (500 MHz, D2O)
f1 (ppm)

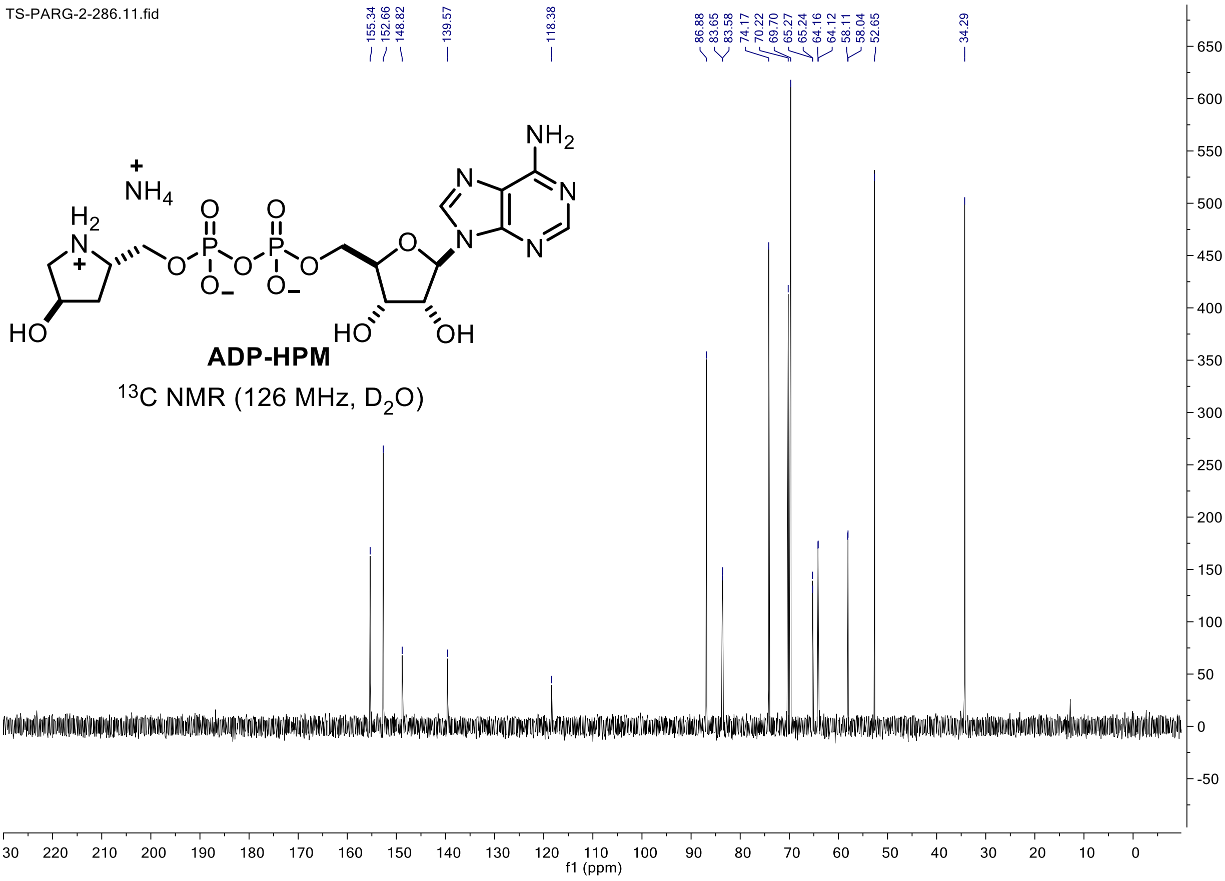
TS-PARG-2-286.11.fid
155.34
152.66
148.82
139.57
118.38
86.88
83.65
83.58
74.17
70.22
69.70
65.27
65.24
64.16
64.12
58.11
58.04
52.65
34.29
ADP-HPM
13C NMR (126 MHz, D2O)
f1 (ppm)

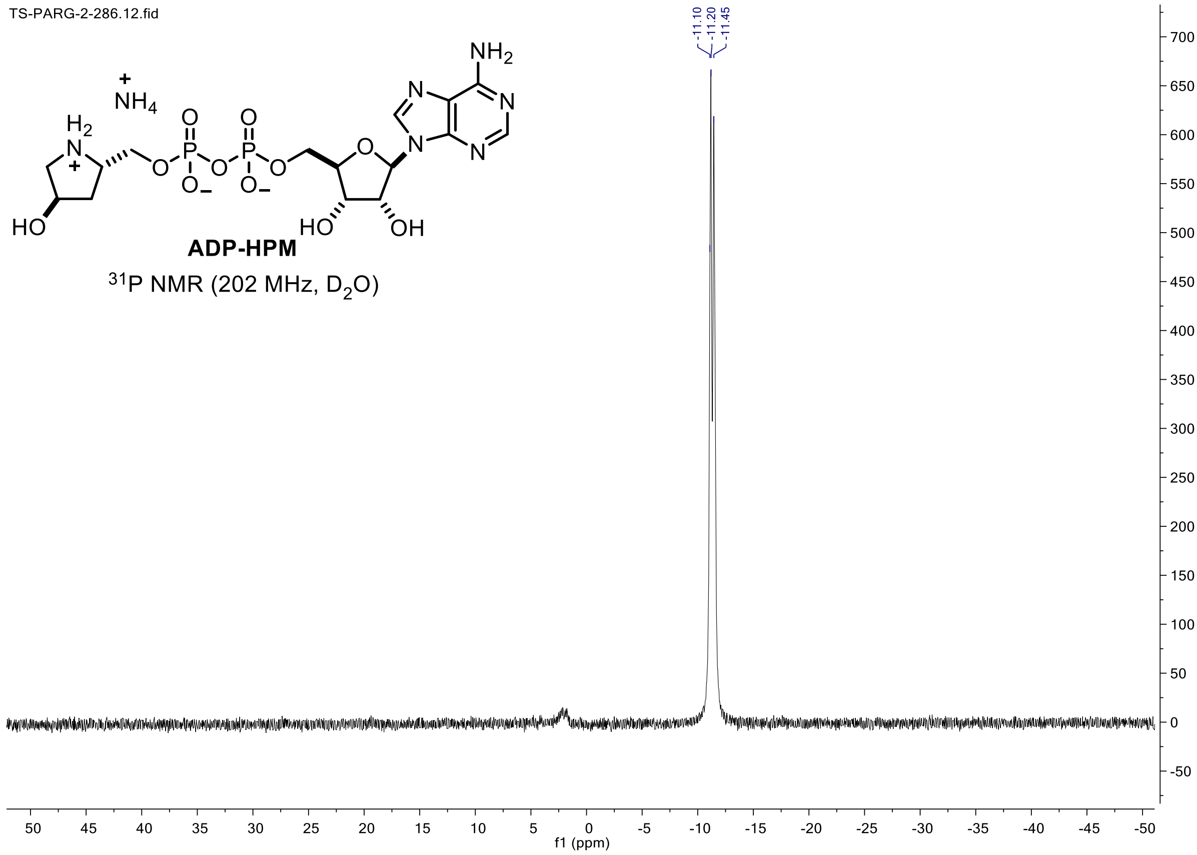

TS-PARG-2-286.12.fid
ADP-HPM
^{31}P NMR (202 MHz, D_2O)
-11.10
-11.20
-11.45
f1 (ppm)

NMR spectra concerning the synthesis of ADP-HPD

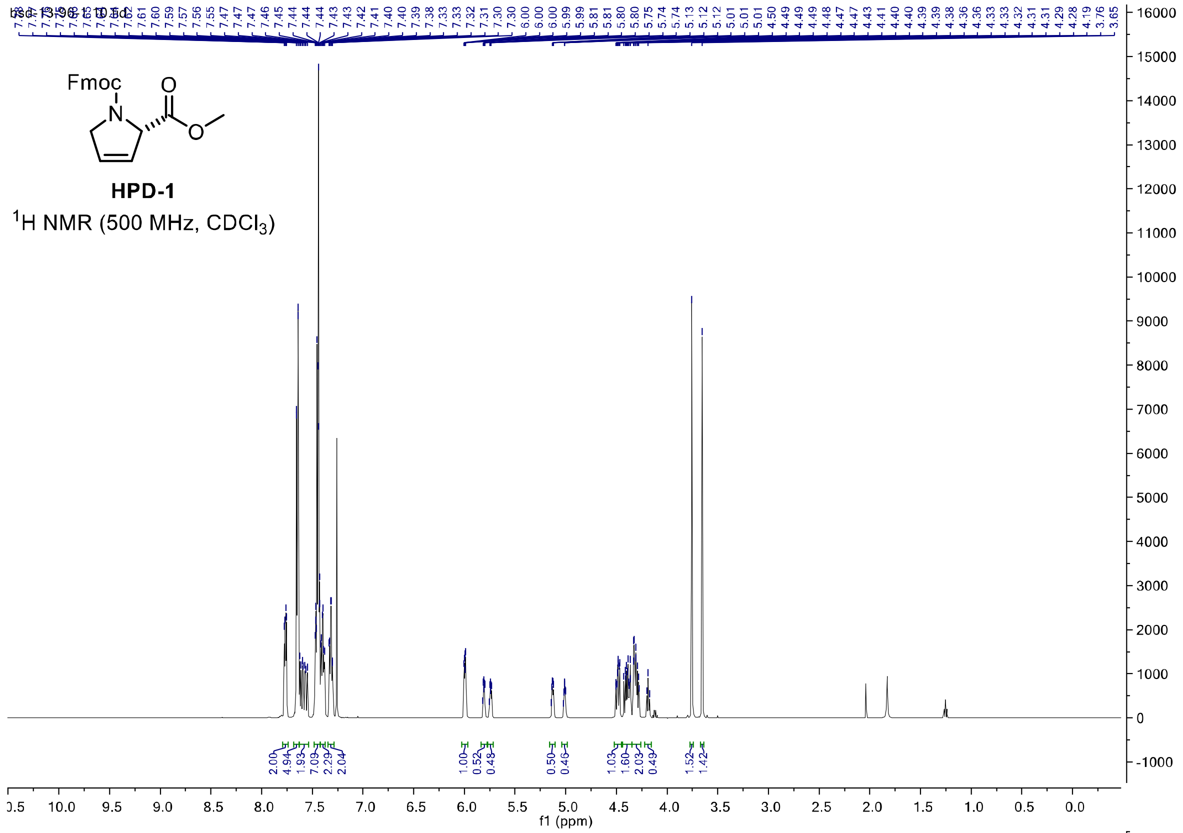

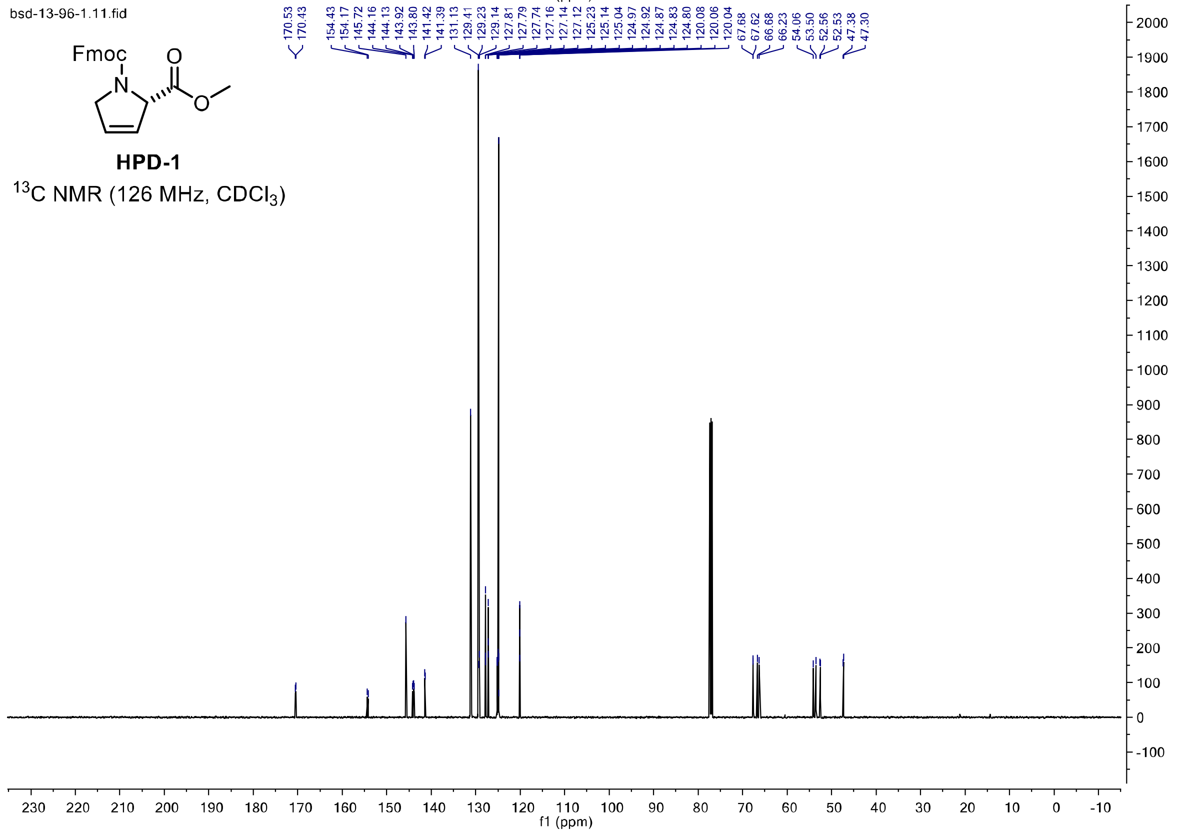

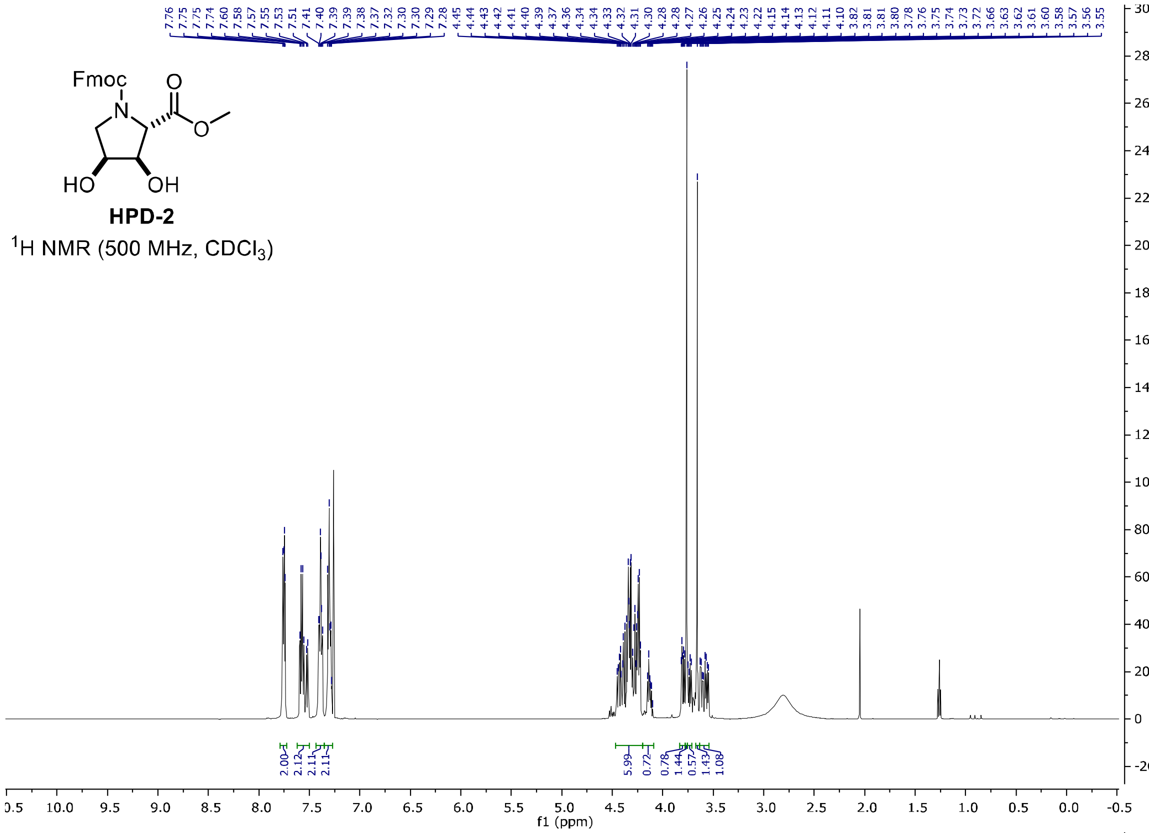
Fmoc
O
N
O
HO
OH
HPD-2
¹H NMR (500 MHz, CDCl₃)
7.76 7.75 7.75 7.74 7.60 7.58 7.57 7.55 7.53 7.51 7.41 7.40 7.39 7.39 7.38 7.37 7.32 7.30 7.30 7.29 7.28
4.45 4.44 4.43 4.42 4.41 4.40 4.39 4.37 4.36 4.34 4.34 4.33 4.32 4.31 4.30 4.28 4.28 4.27 4.26 4.25 4.24 4.23 4.22 4.15 4.14 4.13 4.12 4.11 4.10 3.82 3.81 3.81 3.80 3.78 3.76 3.75 3.74 3.73 3.72 3.66 3.63 3.62 3.61 3.60 3.58 3.57 3.56 3.55
2.00 2.12 2.11 2.11
5.99 0.72 0.78 1.44 0.57 1.43 1.08
f1 (ppm)

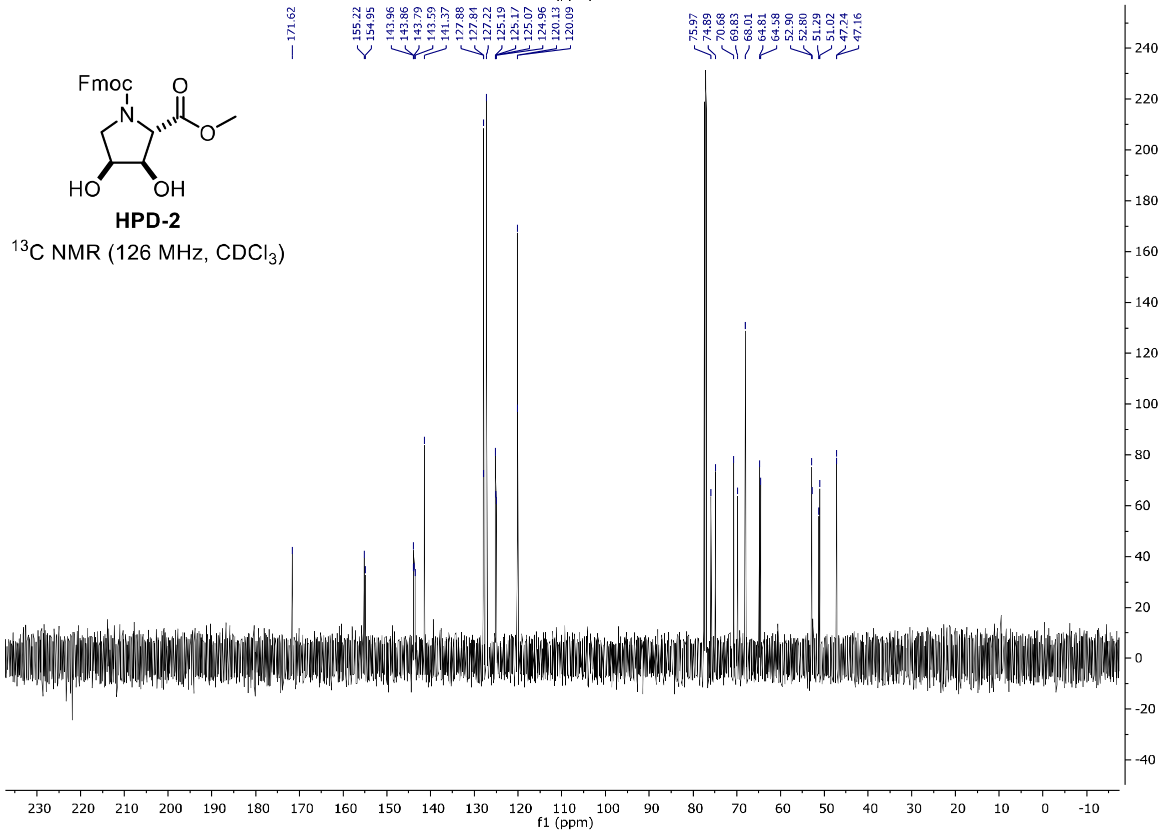
Fmoc
O
N
O
HO
OH
HPD-2
¹³C NMR (126 MHz, CDCl₃)
171.62
155.22 154.95 143.96 143.86 143.79 143.59 141.37 127.88 127.84 127.22 125.19 125.17 125.07 124.96 120.13 120.09
75.97 74.89 70.68 69.83 68.01 64.81 64.58 52.90 52.80 51.29 51.02 47.24 47.16
f1 (ppm)

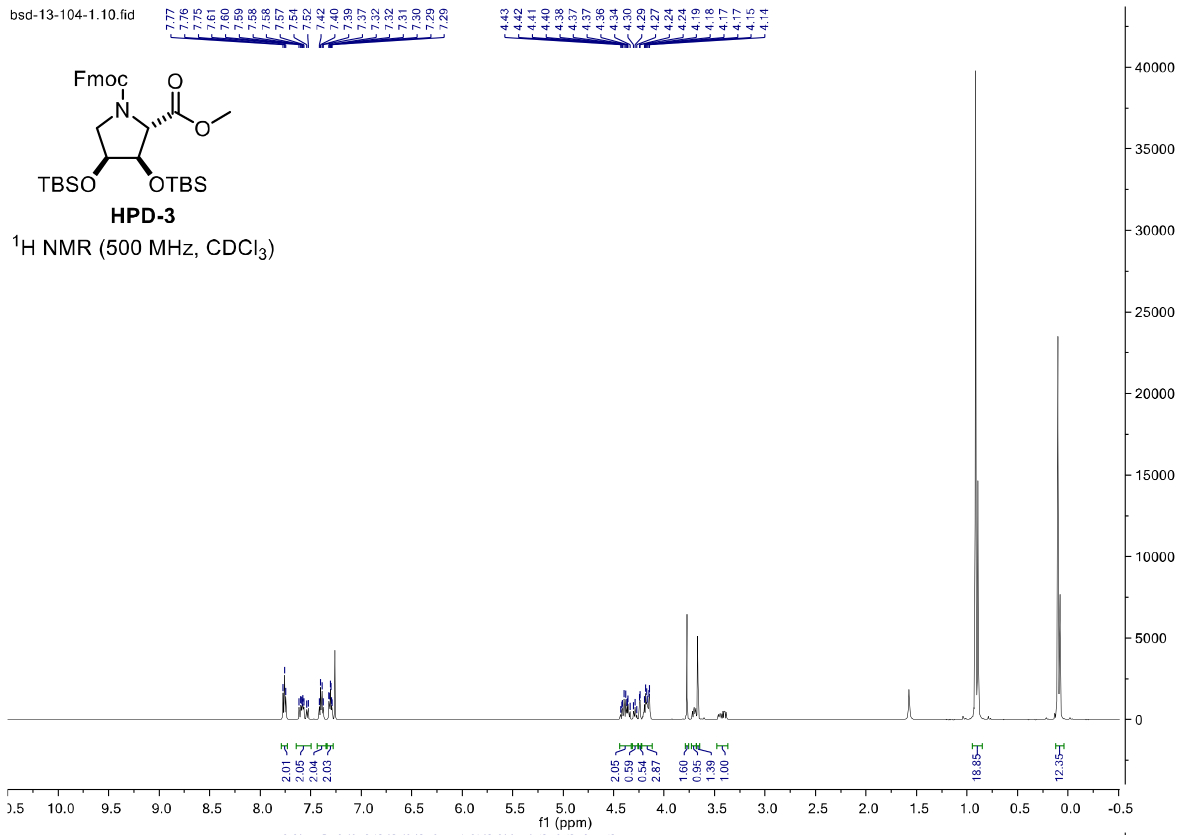
bsd-13-104-1.10.fid
Fmoc
O
N
O
TBSO
OTBS
HPD-3
1H NMR (500 MHz, CDCl3)
7.77 7.76 7.75 7.61 7.60 7.59 7.58 7.58 7.57 7.54 7.52 7.42 7.40 7.39 7.37 7.32 7.31 7.30 7.29 7.29
4.43 4.42 4.41 4.40 4.38 4.37 4.37 4.36 4.34 4.30 4.29 4.27 4.24 4.24 4.19 4.18 4.17 4.17 4.15 4.14
2.01 2.05 2.04 2.03
2.05 0.59 0.54 2.87
1.60 0.95 1.39 1.00
18.85
12.35
f1 (ppm)

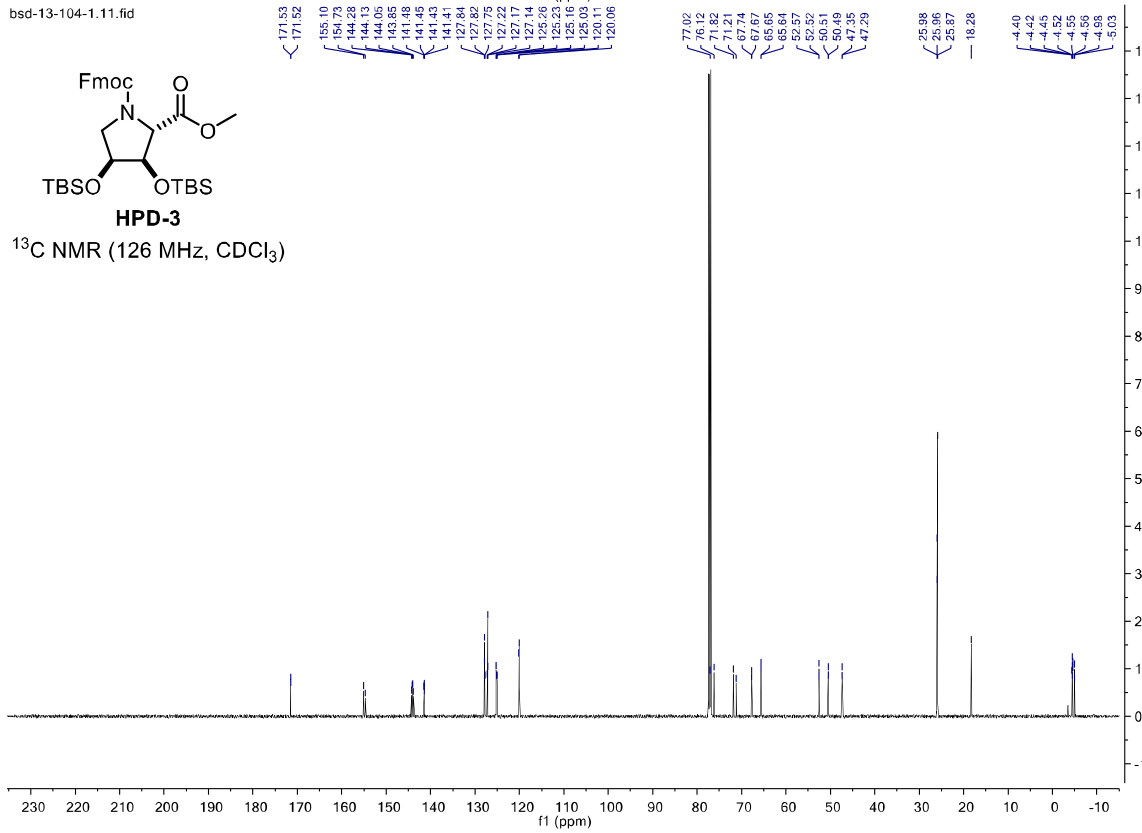
bsd-13-104-1.11.fid
Fmoc
O
N
O
TBSO
OTBS
HPD-3
13C NMR (126 MHz, CDCl3)
171.53 171.52
155.10 154.73 144.28 144.13 144.05 143.85 141.48 141.45 141.43 141.41 127.84 127.82 127.75 127.22 127.17 127.14 125.26 125.23 125.16 125.03 120.11 120.06
77.02 76.12 71.82 71.21 67.74 67.67 65.65 65.64 52.57 52.52 50.51 50.49 47.35 47.29
25.98 25.96 25.87
18.28
-4.40 -4.42 -4.45 -4.52 -4.55 -4.56 -4.98 -5.03
f1 (ppm)

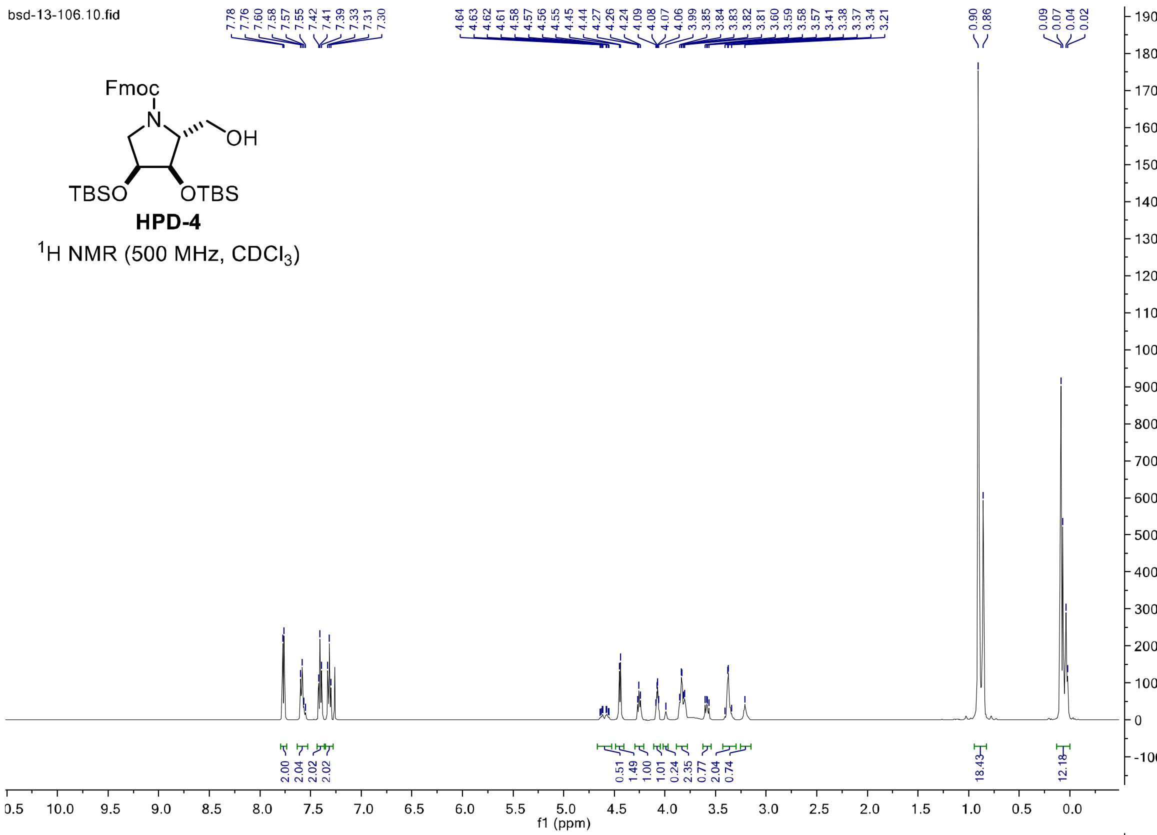
bsd-13-106.10.fid
Fmoc
N
OH
TBSO
OTBS
HPD-4
1H NMR (500 MHz, CDCl3)
7.78 7.76 7.60 7.58 7.57 7.55 7.42 7.41 7.39 7.33 7.31 7.30
4.64 4.63 4.62 4.61 4.58 4.57 4.56 4.55 4.45 4.44 4.27 4.26 4.24 4.09 4.08 4.07 4.06 3.99 3.85 3.84 3.83 3.82 3.81 3.60 3.59 3.58 3.57 3.41 3.38 3.37 3.34 3.21
0.90 0.86
0.09 0.07 0.04 0.02
2.00 2.04 2.02 2.02
0.51 1.49 1.00 1.01 0.24 2.35 0.77 2.04 0.74
18.43
12.18
f1 (ppm)

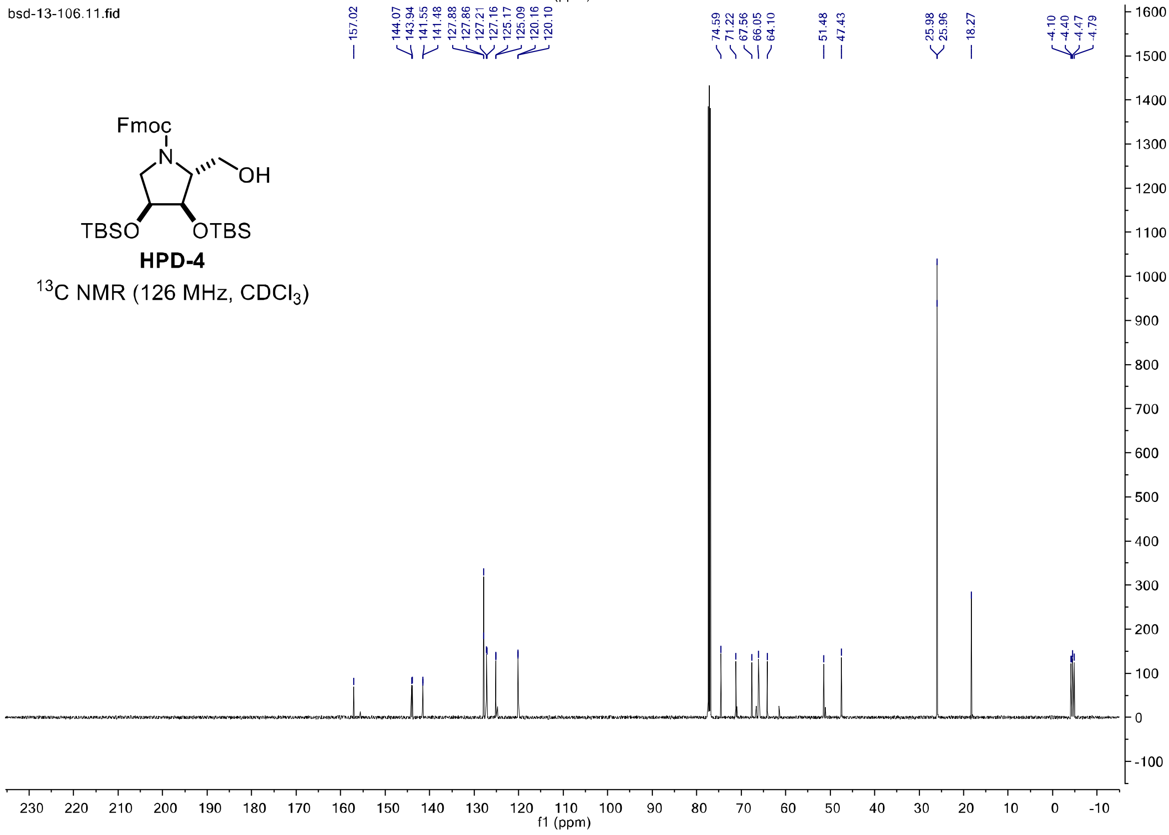
bsd-13-106.11.fid
Fmoc
N
OH
TBSO
OTBS
HPD-4
13C NMR (126 MHz, CDCl3)
157.02
144.07 143.94 141.55 141.48 127.86 127.21 127.16 125.17 125.09 120.16 120.10
74.59 71.22 67.56 66.05 64.10
51.48 47.43
25.98 25.96 18.27
-4.10 -4.40 -4.47 -4.79
f1 (ppm)

180529_bsd-13-80_PROTON

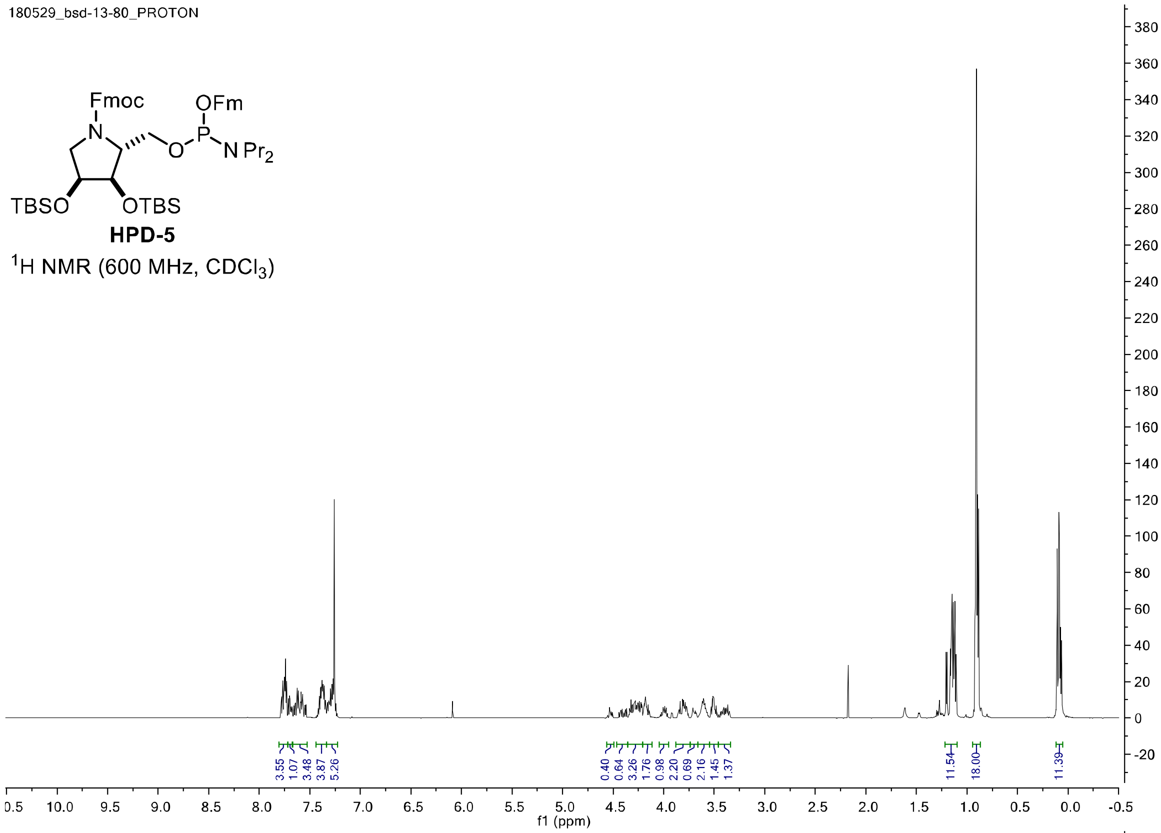

HPD-5

^{1}H NMR (600 MHz, $CDCl_3$)

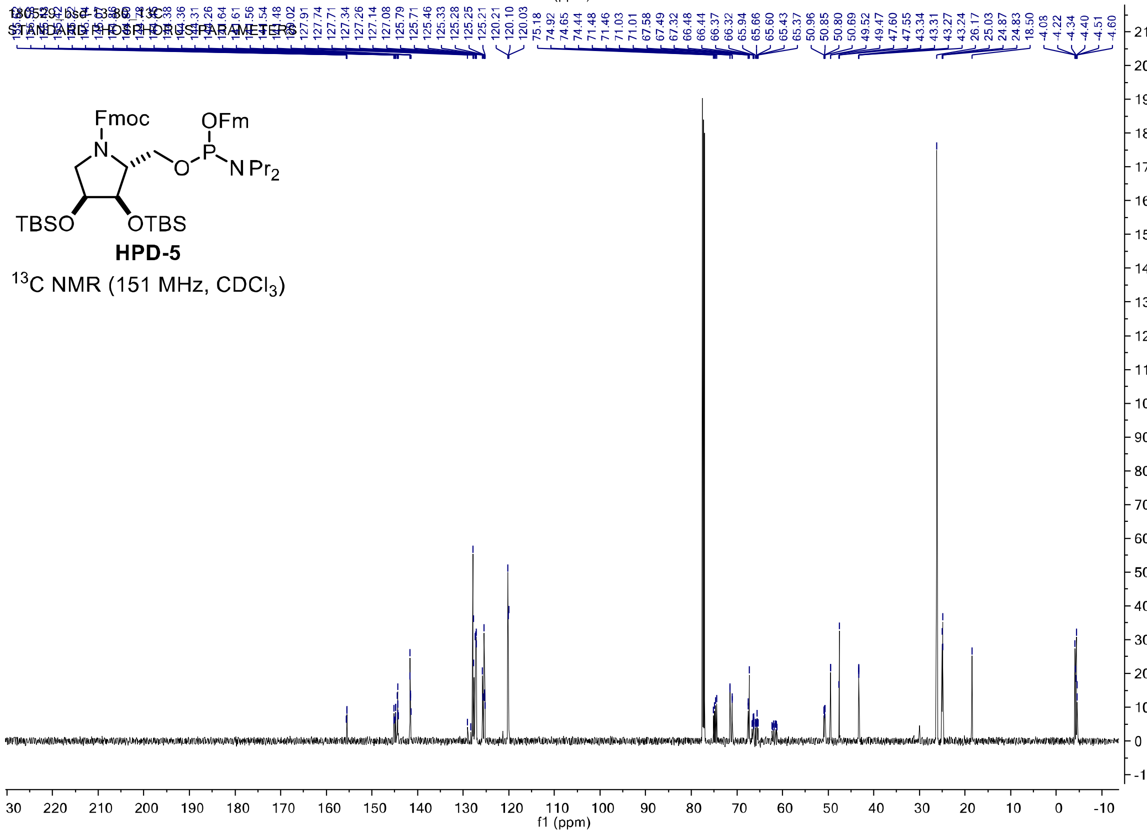

HPD-5

^{13}C NMR (151 MHz, $CDCl_3$)

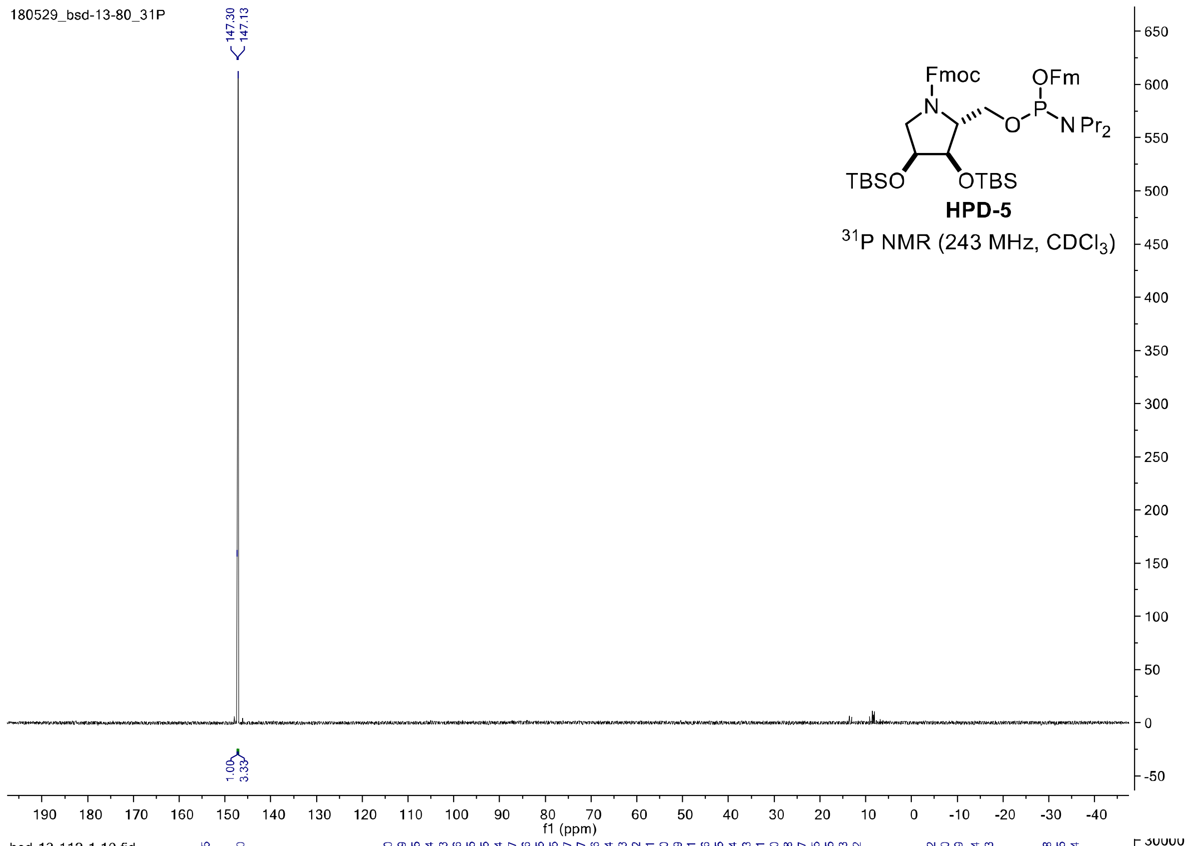
180529_bsd-13-80_31P
147.30
147.13
Fmoc
OFm
N
P
O
N Pr2
TBSO
OTBS
HPD-5
31P NMR (243 MHz, CDCl3)
1.00
3.33
f1 (ppm)

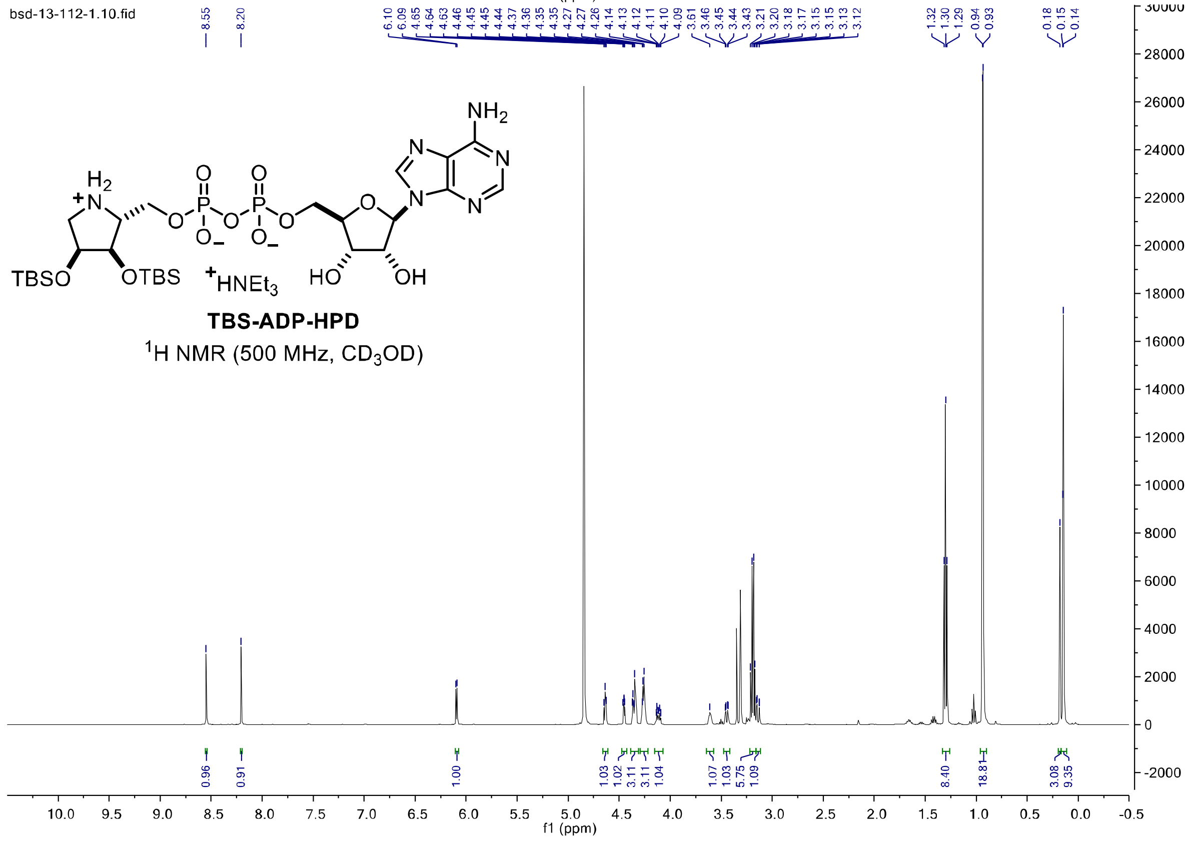
bsd-13-112-1.10.fid
8.55
8.20
NH2
H2
+N
HNEt3
TBSO
OTBS
HO
OH
TBS-ADP-HPD
1H NMR (500 MHz, CD3OD)
0.96
0.91
1.00
1.03
1.02
3.11
3.11
1.04
1.07
1.03
5.75
1.09
8.40
18.81
3.08
9.35
f1 (ppm)

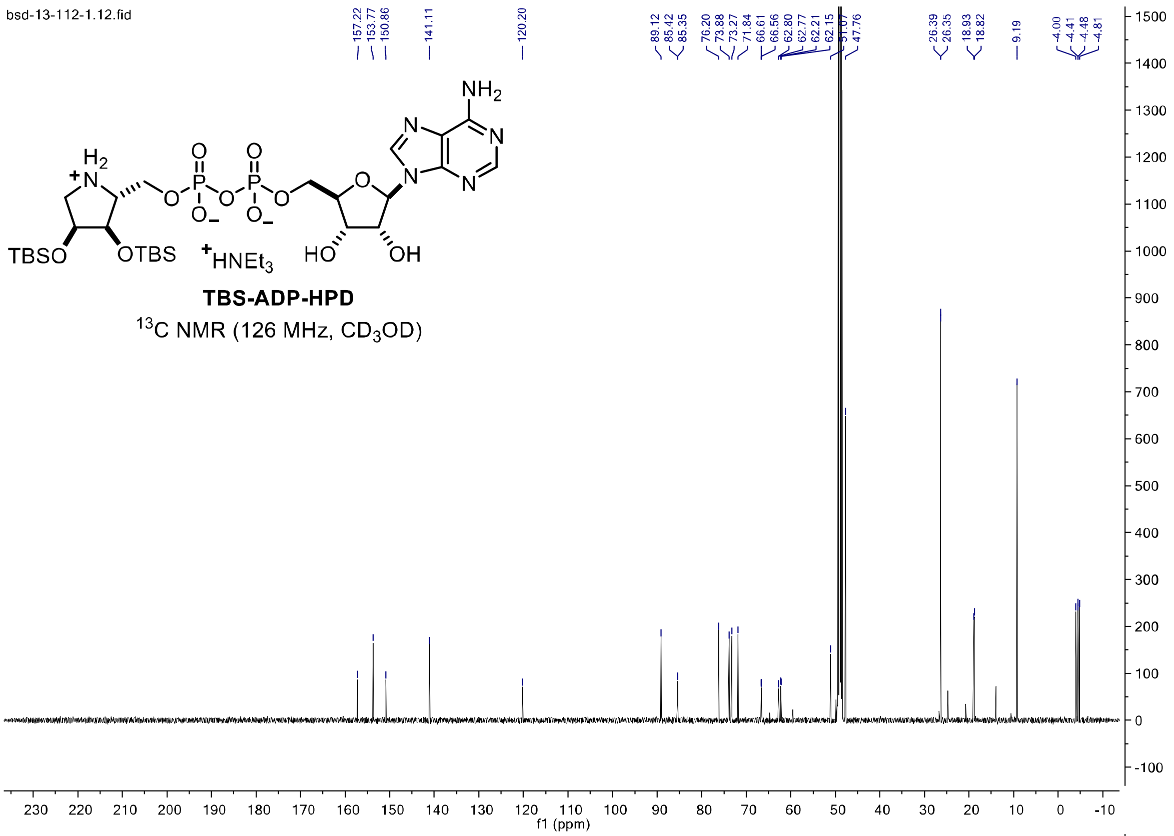
bsd-13-112-1.12.fid
157.22
153.77
150.86
141.11
120.20
89.12
85.42
85.35
76.20
73.88
73.27
71.84
66.61
66.56
62.80
62.77
62.21
62.15
51.07
47.76
26.39
26.35
18.93
18.82
9.19
-4.00
-4.41
-4.48
-4.81
NH2
H2
TBSO
OTBS
+HNEt3
HO
OH
TBS-ADP-HPD
13C NMR (126 MHz, CD3OD)
f1 (ppm)

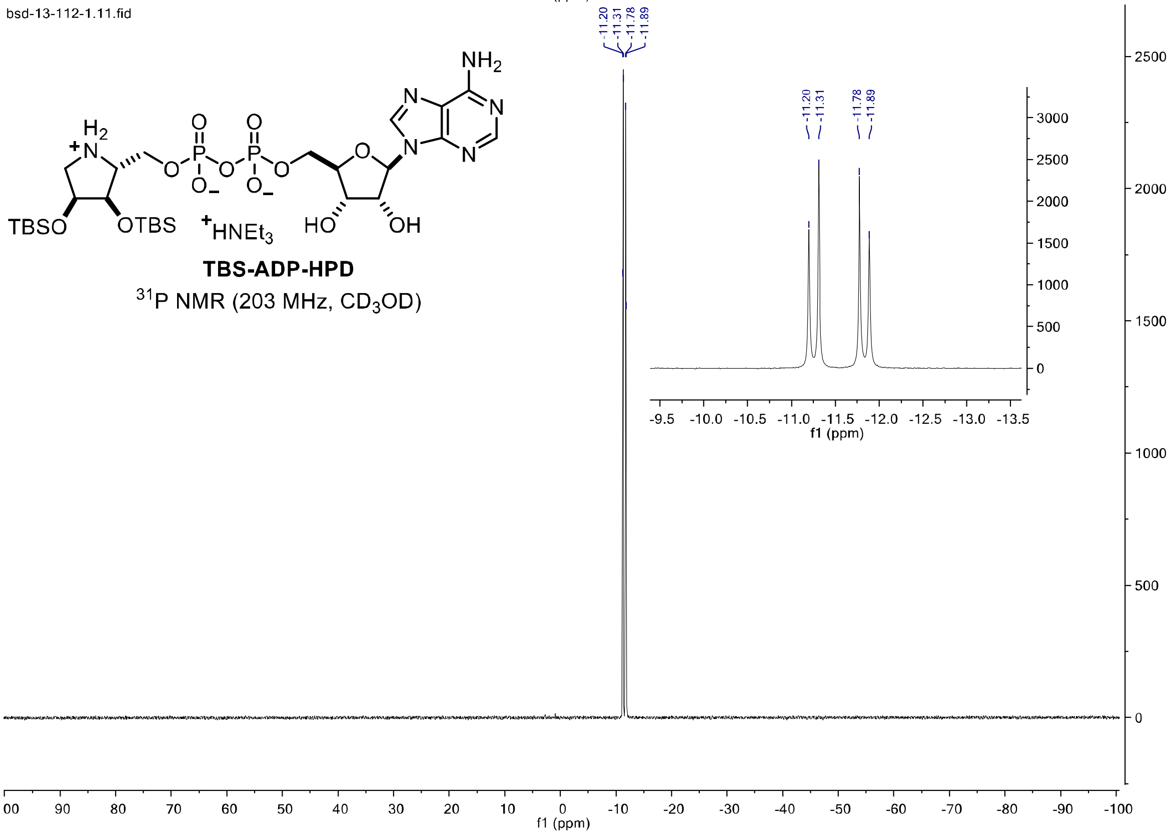
bsd-13-112-1.11.fid
-11.20
-11.31
-11.78
-11.89
NH2
H2
TBSO
OTBS
+HNEt3
HO
OH
TBS-ADP-HPD
31P NMR (203 MHz, CD3OD)
f1 (ppm)

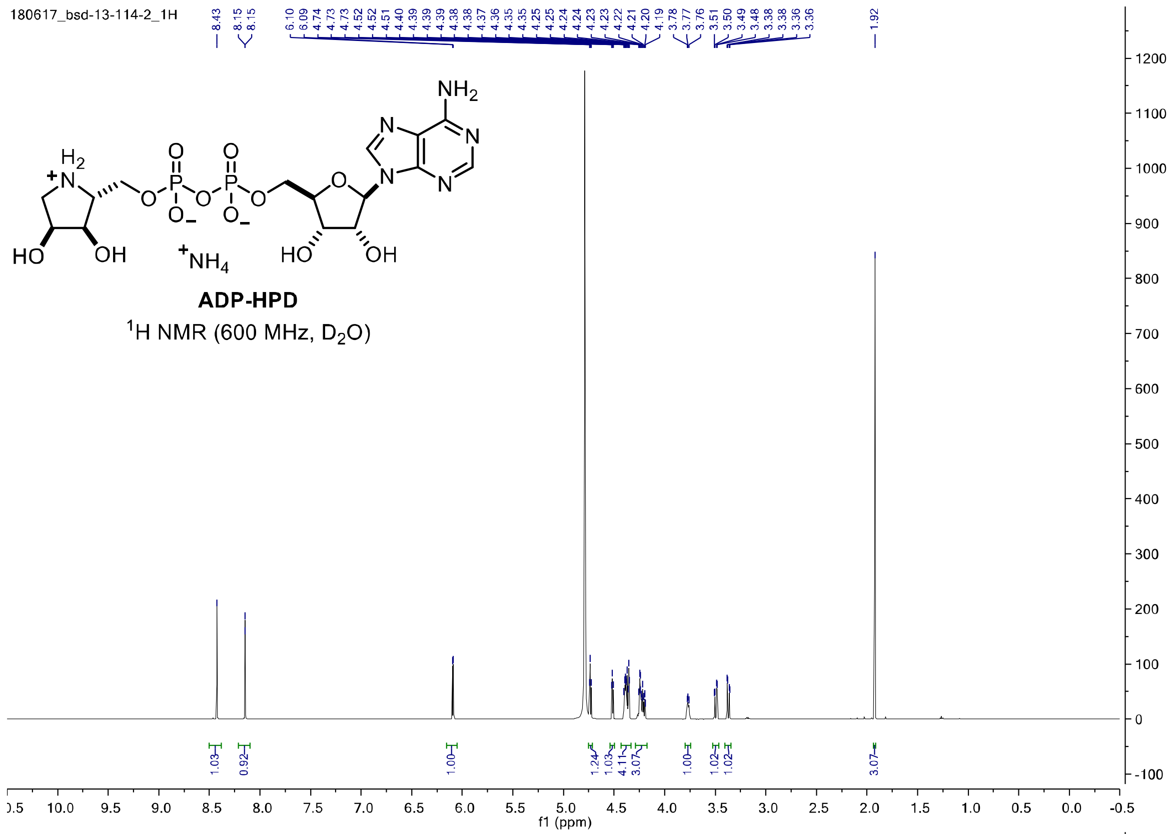
180617_bsd-13-114-2_1H
8.43
8.15
8.15
6.10
6.09
4.74
4.73
4.73
4.52
4.52
4.51
4.40
4.39
4.39
4.39
4.38
4.38
4.37
4.36
4.35
4.35
4.25
4.25
4.24
4.24
4.23
4.23
4.22
4.21
4.20
4.19
3.78
3.77
3.76
3.51
3.50
3.49
3.48
3.38
3.38
3.36
3.36
1.92
NH2
N
N
N
N
H2
+N
O
O
P
P
O
O
O
O
O
O–
O–
HO
OH
+NH4
HO
OH
ADP-HPD
1H NMR (600 MHz, D2O)
1.03
0.92
1.00
1.24
1.03
4.11
3.07
1.00
1.02
1.02
3.07
f1 (ppm)

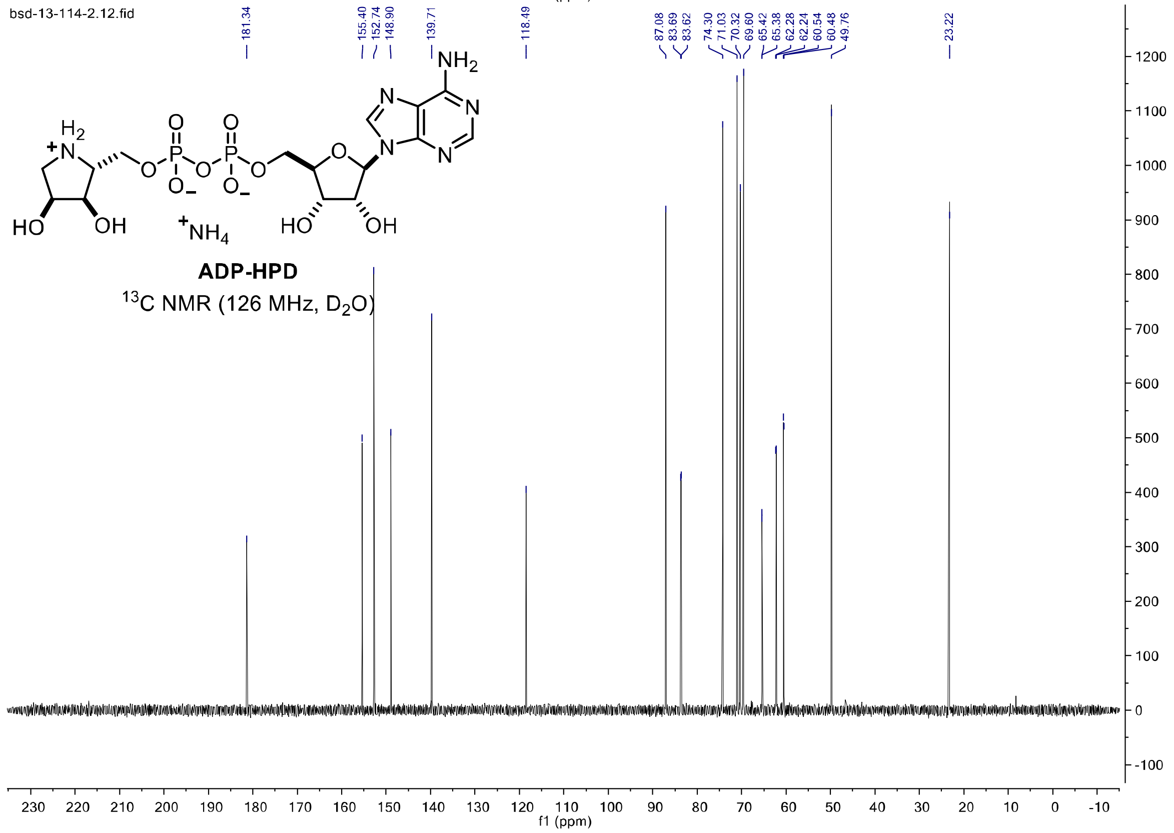
bsd-13-114-2.12.fid
181.34
155.40
152.74
148.90
139.71
118.49
87.08
83.69
83.62
74.30
71.03
70.32
69.60
65.42
65.38
62.28
62.24
60.54
60.48
49.76
23.22
NH2
N
N
N
N
H2
+N
O
O
P
P
O
O
O
O
O
O–
O–
HO
OH
+NH4
HO
OH
ADP-HPD
13C NMR (126 MHz, D2O)
f1 (ppm)

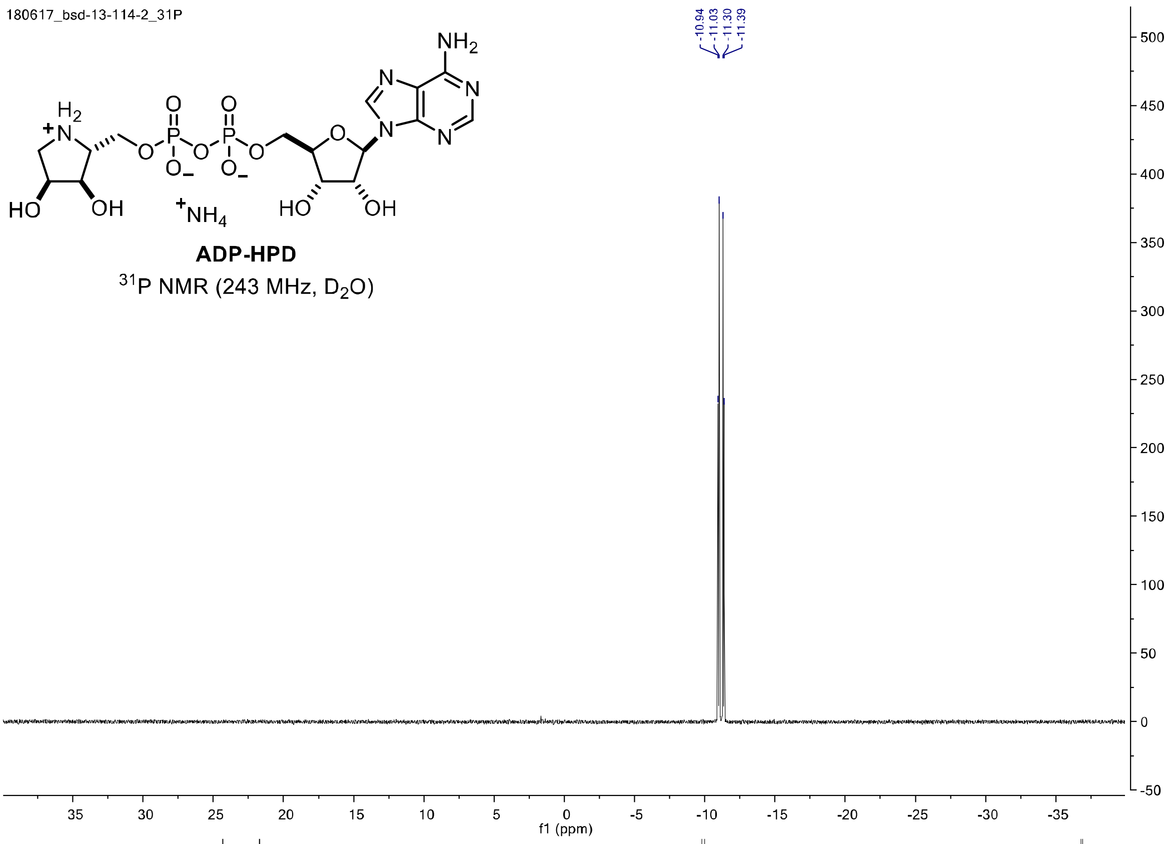
180617_bsd-13-114-2_31P
ADP-HPD
^{31}P NMR (243 MHz, D_2O)
-10.94
-11.03
-11.30
-11.39
f1 (ppm)

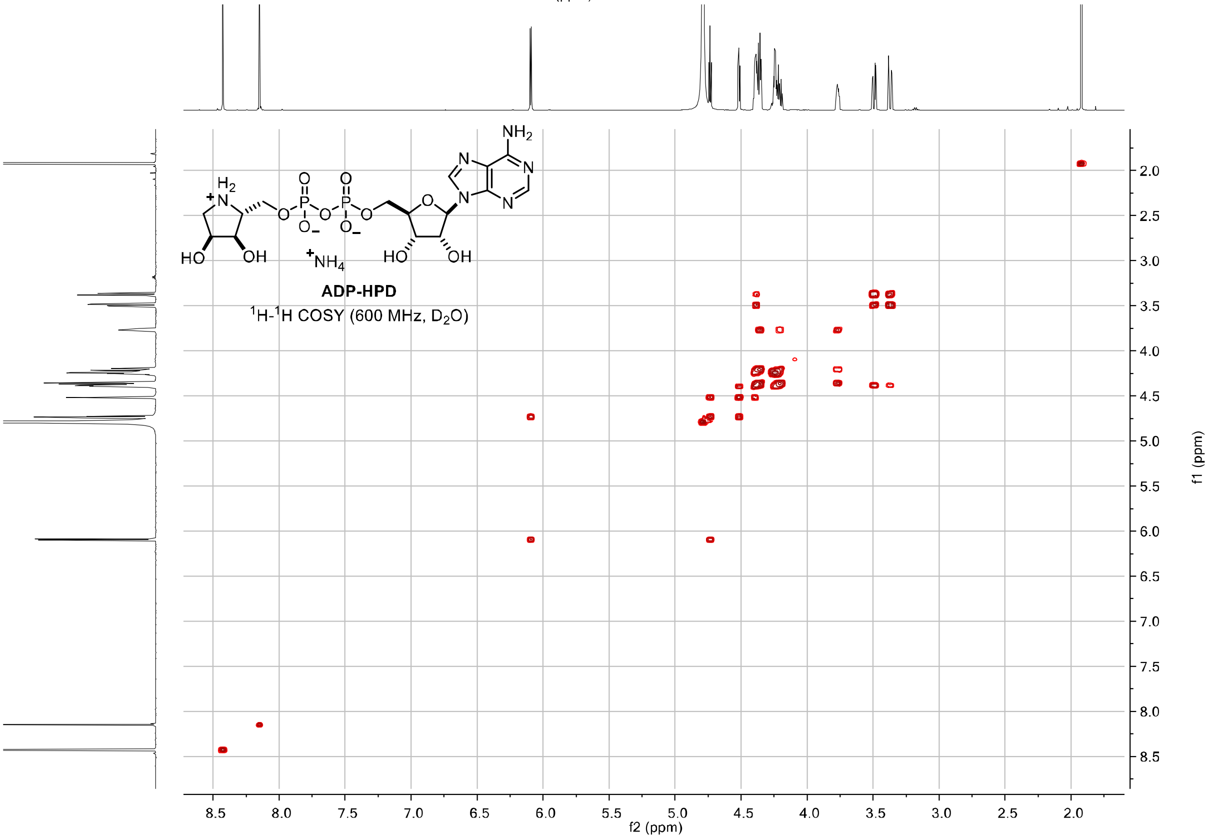
ADP-HPD
^{1}H-^{1}H COSY (600 MHz, D_2O)
f2 (ppm)
f1 (ppm)

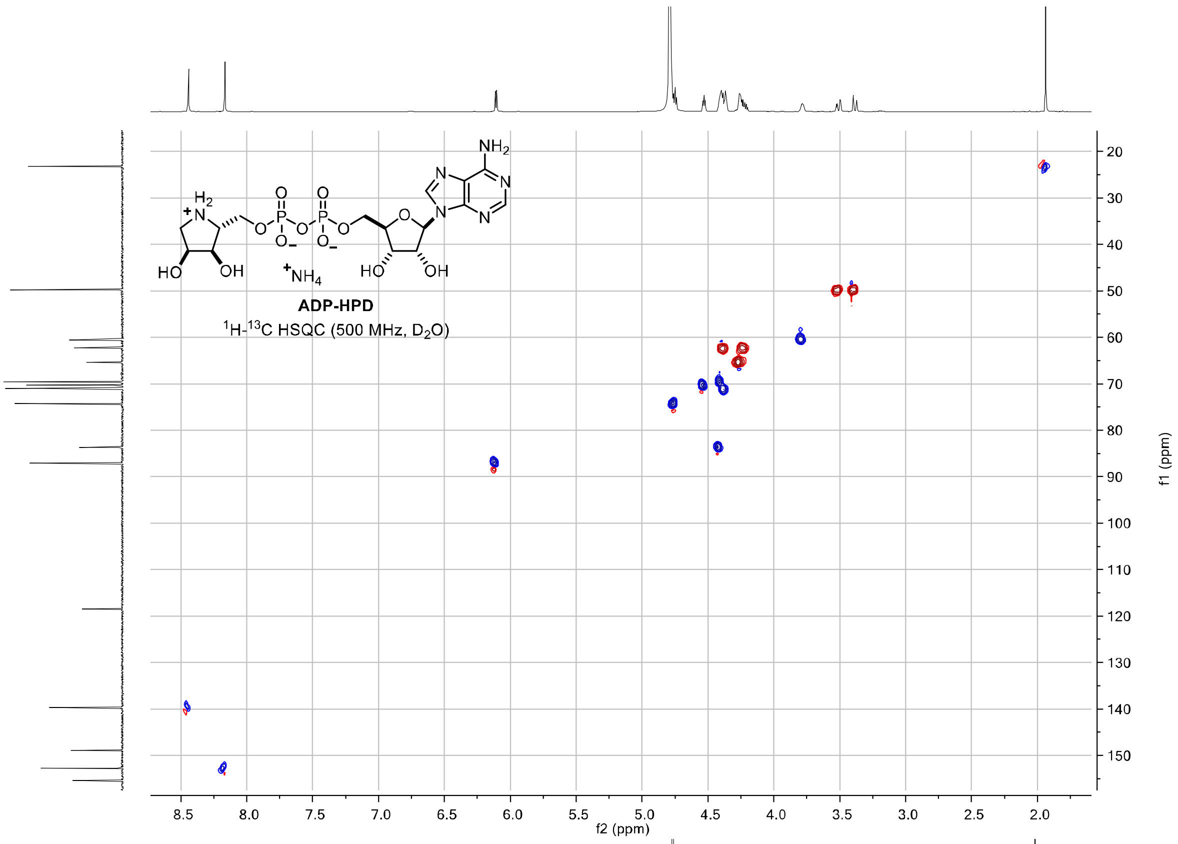
NH2
H2
HO
OH
+NH4
HO
OH
ADP-HPD
1H-13C HSQC (500 MHz, D2O)
f2 (ppm)
f1 (ppm)

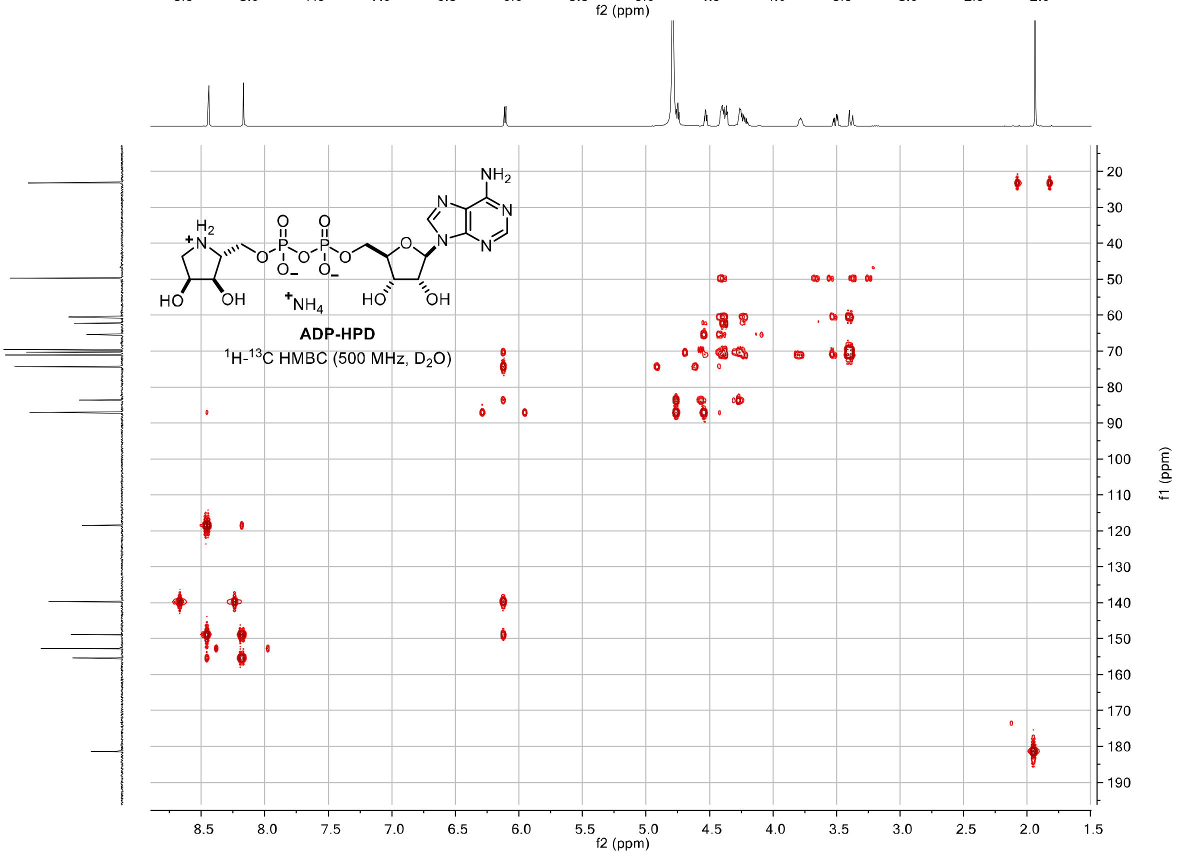
NH2
H2
HO
OH
+NH4
HO
OH
ADP-HPD
1H-13C HMBC (500 MHz, D2O)
f2 (ppm)
f1 (ppm)

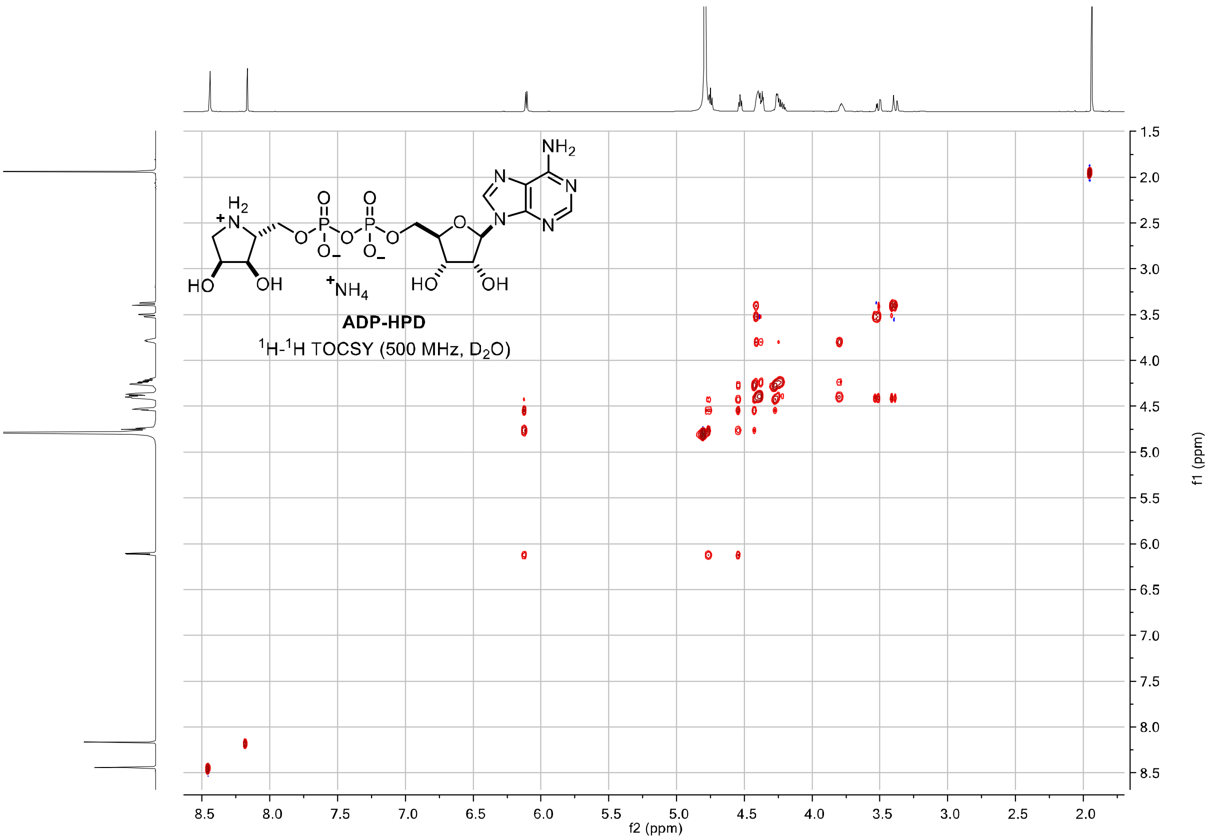
NH2
H2
N
O
P
O
O
P
O
O
O
N
N
N
N
HO
OH
+NH4
HO
OH
ADP-HPD
1H-1H TOCSY (500 MHz, D2O)
f1 (ppm)
f2 (ppm)
1.5
2.0
2.5
3.0
3.5
4.0
4.5
5.0
5.5
6.0
6.5
7.0
7.5
8.0
8.5
